# How to Assess and Predict Electrical Double Layer Properties. Implications for Electrocatalysis

**DOI:** 10.1021/acs.chemrev.3c00806

**Published:** 2024-11-11

**Authors:** Christian M. Schott, Peter M. Schneider, Kun-Ting Song, Haiting Yu, Rainer Götz, Felix Haimerl, Elena Gubanova, Jian Zhou, Thorsten O. Schmidt, Qiwei Zhang, Vitaly Alexandrov, Aliaksandr S. Bandarenka

**Affiliations:** † Physics of Energy Conversion and Storage, Department of Physics, Technical University of Munich, James-Franck-Straße 1, 85748 Garching bei München, Germany; ‡ BMW AG, Petuelring 130, 80809 München, Germany; § State Key Laboratory of Urban Water Resource and Environment, School of Environment, Harbin Institute of Technology, Harbin 150090, People’s Republic of China; ∥ Department of Chemical and Biomolecular Engineering and Nebraska Center for Materials and Nanoscience, University of Nebraska—Lincoln, Lincoln, Nebraska 68588, United States; ⊥ Catalysis Research Center, Technical University of Munich, Ernst-Otto-Fischer-Straße 1, 85748 Garching bei München, Germany

## Abstract

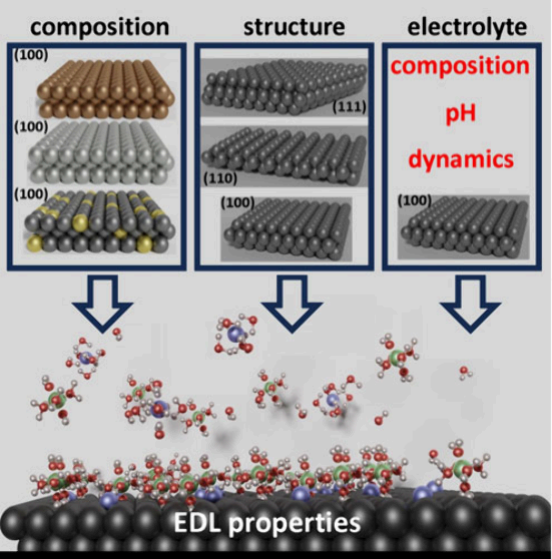

The electrical double layer (EDL) plays a central role in electrochemical energy systems, impacting charge transfer mechanisms and reaction rates. The fundamental importance of the EDL in interfacial electrochemistry has motivated researchers to develop theoretical and experimental approaches to assess EDL properties. In this contribution, we review recent progress in evaluating EDL characteristics such as the double-layer capacitance, highlighting some discrepancies between theory and experiment and discussing strategies for their reconciliation. We further discuss the merits and challenges of various experimental techniques and theoretical approaches having important implications for aqueous electrocatalysis. A strong emphasis is placed on the substantial impact of the electrode composition and structure and the electrolyte chemistry on the double-layer properties. In addition, we review the effects of temperature and pressure and compare solid–liquid interfaces to solid–solid interfaces.

## 1 Introduction

The electrical double layer (EDL) is an interfacial region between an electronic conductor (electrode) and an ionic conductor (electrolyte) that is an intrinsic part of any electrochemical system. The electrode potential is the main control parameter that affects the distribution of species and charges at the electrode/electrolyte interface. The EDL plays an essential role in dictating the mechanisms and rates of electrochemical reactions, and therefore, its deep understanding is critical for advancing electrochemical energy storage and conversion technologies. Helmholtz put forward the first notion of the EDL as early as in 1853.^1^ Since then, the initial EDL model has undergone several key modifications, with the major milestones being the models of Gouy (1909),^2^ Chapman (1913),^3^ Stern (1924),^4^ and Grahame (1947)^5^ that are commonly discussed in electrochemistry textbooks^6−8^ and review articles.^5,9−11^ Due to the complex nature of the electrode/electrolyte interfaces, there has been a historical inclination among scientists to treat the EDL primarily as a simple capacitive component, ignoring many microscopic details of the interface. These simplifications are common in scientific research since it is always a preference to initiate studies with idealized models, which avoid numerous complexities. As the investigations proceeded, models progressively became more complex since experimental results and newly developed theoretical/computational approaches led to a more complete picture. In electrochemistry, polarography has emerged as a key experimental technique facilitating the conceptualization of an electrode as ideally polarizable, i.e., behaving as an electrical capacitor with zero exchange current density. This was accomplished through the utilization of a mercury (Hg) drop as a working electrode in voltammetry as proposed by Jaroslav Heyrovsky in 1922,^12,13^ an achievement for which he was awarded the Nobel Prize in Chemistry (1959). Thus, historically, a dropping mercury electrode played a preeminent role in understanding basic electrochemical properties such as electrocapillarity and double-layer capacitance (*C*_dl_). However, even for such an ideally polarizable electrode as liquid mercury, **C**_dl_ measurements proved to be both complex and challenging. Grahame demonstrated substantial disparities between different *C*_dl_ measurements in the example of sodium fluoride (NaF) solutions at varying concentrations (0.1, 0.01, 0.001 and 0.66 M) in contact with mercury electrodes.^14^ It was hypothesized that the accumulation of anions from the electrolyte on the electrode surface and the reversible electroreduction of ions cause drastic increases in the measured *C*_dl_. Furthermore, the reduction of the dielectric constant of water at the interface can contribute to the deviation of experimentally measured capacitances from those predicted by the classical EDL models.

Theoretical quantification of how much the dielectric constant of water is reduced at various interfaces remains an active area of research.^15−19^ In recent years there have been a growing number of studies demonstrating that presumably inert redox-inactive species from the supporting electrolyte are often not innocent spectators but can significantly influence both reaction rates and mechanisms of electrochemical processes.^20−31^ In a recent investigation^32^ it was suggested based on a combination of electrochemical and first-principles modeling data that the CO_2_ reduction reaction (CO_2_RR) cannot even occur on certain catalysts like Cu, Ag, and Au without the presence of alkali metal cations in the solution. This is quite a remarkable shift in our fundamental understanding of electrocatalytic processes from a negligible or minor influence of electrolyte species in the EDL to a major contribution.

Nowadays, we fully acknowledge the critical role of electrode structure, composition, and electrolyte effects (solution pH, chemistry) and how they manifest in electrochemical phenomena. In electrocatalysis, these properties are routinely exploited to enhance the rates of electrocatalytic processes. Unfortunately, it appears that the fundamental understanding of EDL properties such as *C*_dl_ and how they are related to electrocatalytic behavior are far from comprehensive, severely limiting our advances in electrochemical energy storage and conversion applications.

From an experimental perspective, the question arises of how to optimally and reliably investigate the EDL to gain a deeper fundamental understanding of its characteristics. Generally, two categories of experimental methods are applied: those that microscopically examine the EDL structure by analyzing interfacial water, reaction intermediates, and specifically adsorbed ions or molecules and those that primarily quantify the macroscopic EDL characteristics through parameters such as the potential of zero charge (pzc), differential capacitance (*C*_dl_), and potential of maximum entropy (PME).

The first category includes scanning tunneling microscopy (STM) based approaches, which have been pivotal in investigating the adsorption of H_2_O^33^ or, more specifically, the wetting layer of ice on single crystals to facilitate the observation of superstructure periodicities at low temperatures.^34,35^ We highlight pioneering studies, for instance, from Jacob and co-workers in 2007,^36^ who combined STM with density functional theory calculations, in order to develop an atomistic view of metal–electrolyte interfaces for the very first time. Furthermore, Hugelmann and Schindler^37^ explored the tunneling probability and discovered an exponential decay of the tunneling current with the gap width with an oscillation period of 0.35 nm, consistent with the spacing of water molecules in the Helmholtz layer of the solid/liquid interface from the theoretical prediction. Similar considerations were extended by Simeone et al.,^38^ who detected the nonoscillatory behavior of the effective barrier height at the potential of zero charge. More recently, video-STM has been used to study the surface dynamics of adsorbates^39^ and the variation of adsorption sites,^40,41^ which are critical for the electrochemical processes in the EDL. Interfacial water and adsorbates have also been extensively investigated using various diffraction- and scattering-based techniques. We acknowledge the seminal work of Toney et al.^42^ from the early 1990s, who explored the water density distribution perpendicular to Ag(111) surfaces under potential control using X-ray scattering.^43^ Besides X-ray scattering or diffraction-based techniques,^44−50^ numerous spectroscopic techniques have emerged in recent years with countless different configurations, including X-ray absorption spectroscopy (XAS),^51,52^ electrochemical infrared spectroscopy (EC-IR),^53,54^ electrical transport spectroscopy (ETS),^55^ sum frequency generation (SFG) spectroscopy,^56−59^ and Raman based techniques.^60−62^ All of those techniques allow for the study of the EDL’s structure and composition and dynamic EDL processes related to both adsorbing and nonadsorbing species.

The second category of techniques employed to investigate the EDL involves experimental approaches that do not provide direct visualization or elucidation of the electrode–electrolyte interface structure or adsorbing species, but rather macroscopically quantify the overall EDL through key parameters such as the pzc, *C*_dl_, and PME. Those approaches predominantly rely on electrochemical testing, such as in the case of cyclic voltammetry (CV),^63,64^ linear sweep voltammetry (LSV),^65^ galvanostatic charge/discharge (GCD),^66^ electrochemical impedance spectroscopy (EIS),^67,68^ and CO-displacement approaches.^69,70^ However, the *C*_dl_ and pzc seem to be significantly influenced by variations in the experimental conditions, leading to notable differences in the respective values obtained through different methods, which requires further investigations.^71,72^ Additionally, scanning electrochemical microscopy techniques^73−75^ have been gaining increasing interest in investigating either *C*_dl_ or pzc. A complex setup needs to be employed to investigate the entropy of a catalytic system in order to detect the PME. So far, the prevailing state-of-the-art methods are based on laser-induced current transient (LICT) approaches,^76^ while additional methods to determine the PME are scarce.

The progress of theoretical methods can be traced to the 1960s when Stillinger and Kirkwood^77^ introduced liquid-state theories to illustrate molecular excluded-volume effects on ion distribution in the diffusion layer, followed by theoretical studies based on integral equation theory and deformations of the Poisson–Boltzmann (PB) equation. Evans and Sluckin^78^ in 1980 first used classical density functional theory (cDFT) to describe the interfacial properties of charged fluids becoming an important theoretical tool to predict the structure, capacitance, and dynamic behavior of the EDL.^79−81^ With the advent of quantum mechanical based density functional theory (DFT) and an increase in computational power, the application of first-principles approaches to electrochemical problems has become commonplace and proved to be extremely successful. Nevertheless, from a quantum chemistry perspective, electrochemical interfaces still represent some of the most outstanding challenges. For example, in the case of the oxygen evolution reaction (OER), an electrochemical system involves three phases: solid (electrode), liquid (electrolyte), and gas (reaction product, O_2_). This requires the use of a quantum chemical method that provides a reasonable description of electronic structure properties for all three distinct phases. However, DFT functionals already struggle with O_2_, significantly overestimating the molecule binding energy and thus requiring an experimental correction. The electrode surface might also change its structural and chemical properties during electrochemical processes, e.g., transitioning from a metallic to a semiconducting phase, because of surface oxidation under the OER conditions. The presence of a liquid phase, in turn, affects interfacial charge transfer processes, modifying both enthalpic and entropic contributions to the overall reaction free energy. In addition, even electrochemically nonactive ions from the supporting electrolyte may directly (e.g., by coadsorption) or indirectly (e.g., electrostatically) affect interfacial electrochemical phenomena. This may lead to changes in both the reaction mechanisms and kinetics in a nontrivial manner. Moreover, the statistical nature of a liquid electrolyte requires performing proper statistical averaging over sufficiently long simulation times. In addition, these simulations ideally should be carried out under electrode potential control and account for long-range electrostatic effects to be consistent with experimental conditions. This enormous complexity of electrochemical interfaces requires a deliberate choice of approximations when building a theoretical model and careful comparison with experimental data.

The computational hydrogen electrode (CHE) approach introduced in 2004^82^ has become one of the most fruitful theoretical approaches in computational electrochemistry. It enables the analysis of electrochemical reactions involving coupled interfacial H^+^–e^–^ transfers at the DFT level, avoiding the need to compute the energies of charged species. However, this method ignores the effects of the explicit electrode potential and the formed EDL. To partially remedy this limitation, implicit solvent models capable of capturing the capacitive charging behavior of the interfaces are currently being applied and implemented in a variety of codes. However, these methods also face challenges, such as the inability to account for the effects of dynamically changing H-bonding networks and the effects brought about by electrolyte ions. To address some of these shortcomings and account for the statistical nature of the EDL, theoretical methods such as classical, ab initio, and deep potential molecular dynamics involving explicit or hybrid (implicit/explicit) solvents can be particularly useful. We refer the reader to several recent reviews on the application of theoretical modeling to study basic properties of metal/aqueous interfaces.^11,83−88^ We also note that extensive theoretical literature is now available on the role of the EDL in electrocatalysis.^81,89−94^

To develop a more comprehensive theory beyond the foundational aspects of the classical EDL theory, significant modifications are necessary. The classical model for solid/liquid interfaces is based on a dilute-solution approximation and thus avoids the consideration of strong Coulombic interactions, which are commonly discussed in studies of ionic liquids.^95−99^ The dilute-solution approximation emerges as a problem since, even in the case of dilute electrolytes, the ion concentration at the electrode/electrolyte interface can significantly exceed the concentration in the bulk, sometimes approaching or exceeding the classical solubility limit of ions due to the influence of strong electric fields. To understand this, we need to explore the parameters influencing the EDL properties from a microscopic point of view. We highlight the complexity of these interfaces and their distinct properties arising from multiple factors, such as the effect of the employed electrolyte and the electrode composition and structure. This complexity of the solid/liquid interface has limited the theoretical progress in this area. As a result, except for the classical plots by Grahame for a Hg electrode in NaF electrolytes, there is a lack of quantitative comparisons between theoretical predictions and experimentally measured double-layer capacitances in the existing literature.

Based on these considerations, in this review, we underscore the significance of the electrode composition and structure, as well as the employed electrolyte, in influencing the properties of the EDL. We point out that the impact of these factors on EDL properties pervades beyond aqueous electrolytes, extending to ionic liquids, and also offers insights into solid electrolytes. Furthermore, we highlight the effect of temperatures on EDL properties. Our goal is to provide an overview of experimental and theoretical investigations toward accurate, consistent, and reliable predictions of EDL properties. Therefore, we focus on recent scientific work and provide a historical perspective on various measurements and theoretical approaches. All of the work that was conducted underscores the dependency of the EDL on multiple key factors such as electrode composition, structure, and electrolytes. Our discussion of computational results primarily focuses on first-principles-based simulations. The understanding and determination of EDL properties are essential for improving the field of electrocatalysis since the EDL strongly affects the activity of electrochemical interfaces, as will be discussed in this review. Although some correlations were found between basic EDL properties and electrocatalytic activity, it remains to be understood how exactly EDL characteristics are related to the electrocatalytic behavior of different aqueous electrode/electrolyte interfaces under varying reaction conditions. We hope this review will facilitate further research and contributions to this area of electrochemistry.

## 2 Theory of the Electrochemical Double Layer

### 2.1 EDL at Solid–Liquid Interfaces

#### 2.1.1 Aqueous Electrolytes

Helmholtz introduced the concept of the EDL as an interfacial region comprised of two layers of opposite charges separated by a small distance.^1^ These charges arise from the electrode surface and the counterions in the electrolyte. Since the solvation effects of ions were not included, the separation between the charged electrode surface and the adsorbed electrolyte ions was determined by the radius of the ion. This simple model represents the EDL as a plate capacitor that should lead to a linear voltage drop between the charged layers. While this simple model provides some estimates for double-layer capacitances, it does not capture the detailed microscopic behavior of electrode–electrolyte interfaces.^100^ Furthermore, it does not explain the correlation between the differential capacitance, called the Helmholtz capacitance *C*_H_, and the applied electrode potential. To provide a more accurate description of the solid–liquid interfaces, Gouy^2^ and Chapman^3^ proposed a model based on statistical mechanics and using the Poisson–Boltzmann equation to allow for the spatial distribution of ions in the EDL. This model considers ion movement due to the influence of temperature, leading to a nonuniform ion distribution in a diffuse layer region. The concentration of ions in this layer is influenced by the resulting electrostatic forces leading to specific ion concentration profiles in the vicinity of the electrode.^6^

**Figure 1:**
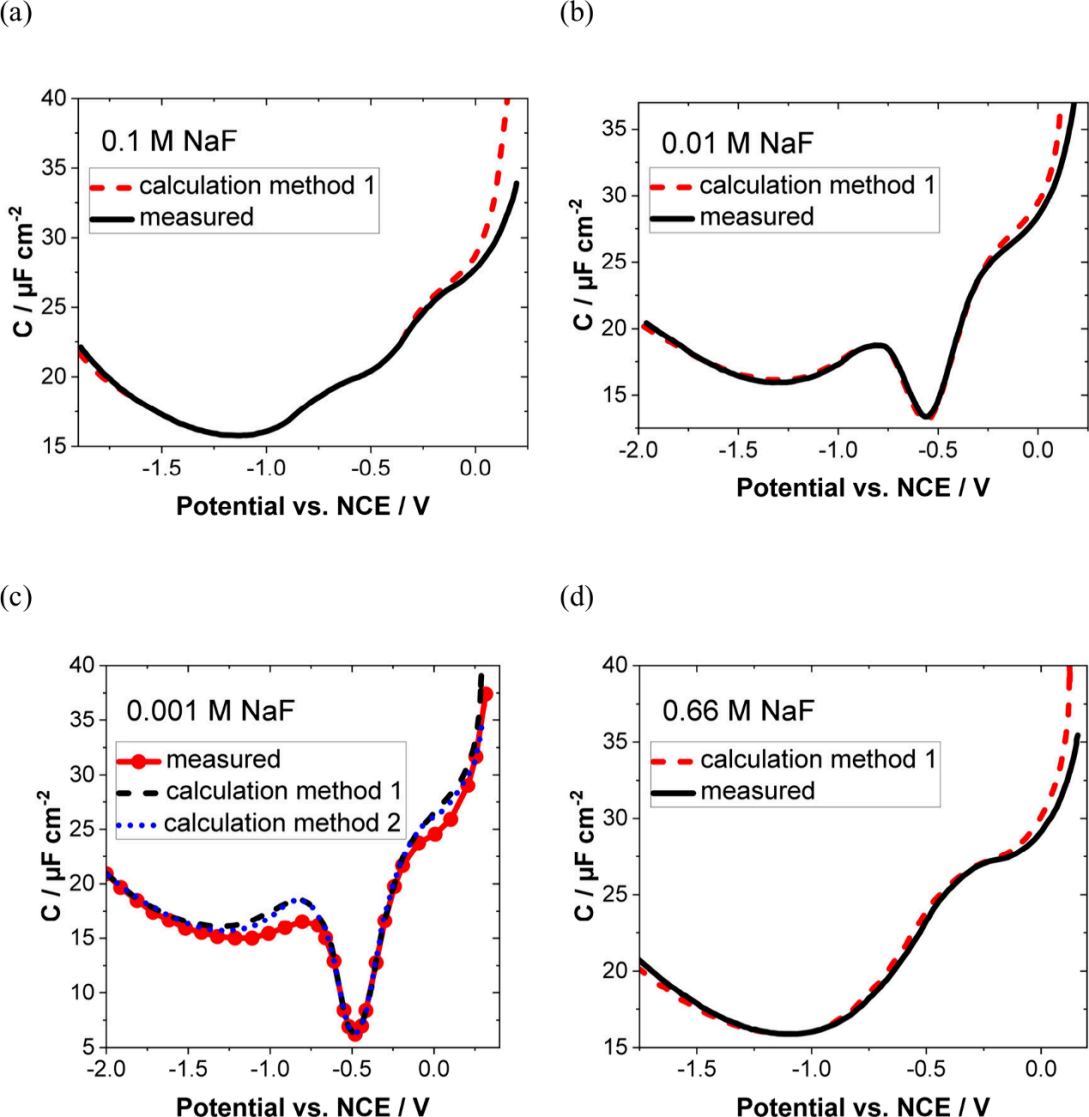
(a–d) Experimental dependencies of double-layer capacitances on the electrode potentials of a Hg electrode in NaF electrolytes of different concentrations together with the calculated curves according to classical theory. Adapted from ref (14). Copyright 1954 American Chemical Society.

However, according to the Poisson equation, the ions are treated as point charges, allowing them to approach the electrode surface unrealistically close, especially at large ion concentrations or large applied electrode potentials. This model leads to the prediction of enormous double-layer capacitances that cannot be observed experimentally. Stern^4^ addressed this issue by introducing a layer at a distance δ from the electrode that represents the minimum separation distance between the electrode and ions, similar to the concept of the Helmholtz plane. For the region beyond δ, the description of the diffuse layer according to the Gouy–Chapman model remains relevant. The modification conducted by Stern provides a more accurate representation of the solid–liquid interface, especially at elevated ion concentrations and under significantly larger applied electrode potentials. However, due to its inherent simplifications and assumptions, the Gouy–Chapman–Stern model typically fails to predict real-world scenarios quantitatively. For instance, it does not account for all physical and chemical processes occurring during specific adsorption of ions. Consequently, it only provides reasonable predictions for a limited number of electrolytes, such as NaF. Again, we highlight the pioneering work by Grahame^14^ (Figure 1), who demonstrated a reasonable agreement between the calculated *C*_dl_ curves using the classical EDL theory and the experimental *C*_dl_ data for a mercury electrode in contact with 0.1, 0.01, 0.001, and 0.66 M NaF solutions. The reason for the good agreement in this case can be attributed to the fact that the hydration shells are strongly attached to the ions and prevent them from dehydrating near the electrode surface.^101^ Conversely, larger anions, such as Cl^–^ or Br^–^, exhibit a significantly weaker ion–solvent interaction and thus enhance specific adsorption at the electrode.^102^

Additionally, cations typically possess more rigid solvation shells than anions due to their smaller sizes,^7^ making anions more prone to adsorb on the electrode specifically. For this reason, the Gouy–Chapman–Stern model was further developed by Grahame,^5,103^ Devanathan,^104^ and Bockris,^105^ for instance, by refining the rigid layer introduced by Stern into inner and outer Helmholtz planes (IHP and OHP) and involving the impact of solvent dipoles.^106^ This modification allowed for a clearer differentiation between solvated and bare ions, particularly highlighting specific adsorption processes in the IHP region. It is essential to point out that specific adsorption of ions at the electrode and accounting for solvent dipole interactions lead to a significantly higher level of complexity of the theoretical model for solid–liquid interfaces. We avoid delving into these details since it will detract from the primary emphasis of our review. Instead, we provide only foundational knowledge about the EDL and focus on the simplified Gouy–Chapman–Stern model. The mathematical framework of the Gouy–Chapman–Stern model, which will be presented in the following, is based on the comprehensive description of this classical model by Bard and Faulkner.^6^

The first region of the Gouy–Chapman–Stern model corresponds to the Helmholtz region that Stern defined as the rigid ion layer with distance δ from the electrode surface, called the Stern layer. The variable *x* describes the current distance from the electrode. Therefore, in the range 0 < *x* < δ, the charge density σ and the associated differential Helmholtz capacitance *C*_H_ can be defined by eqs 1 and 2, respectively. The additional parameters in this context, ε and ε_0_, denote the dielectric constants of an electrolyte medium and vacuum, respectively. The voltage drop is represented by *V*.
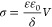
1
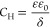
2

**Figure 2:**
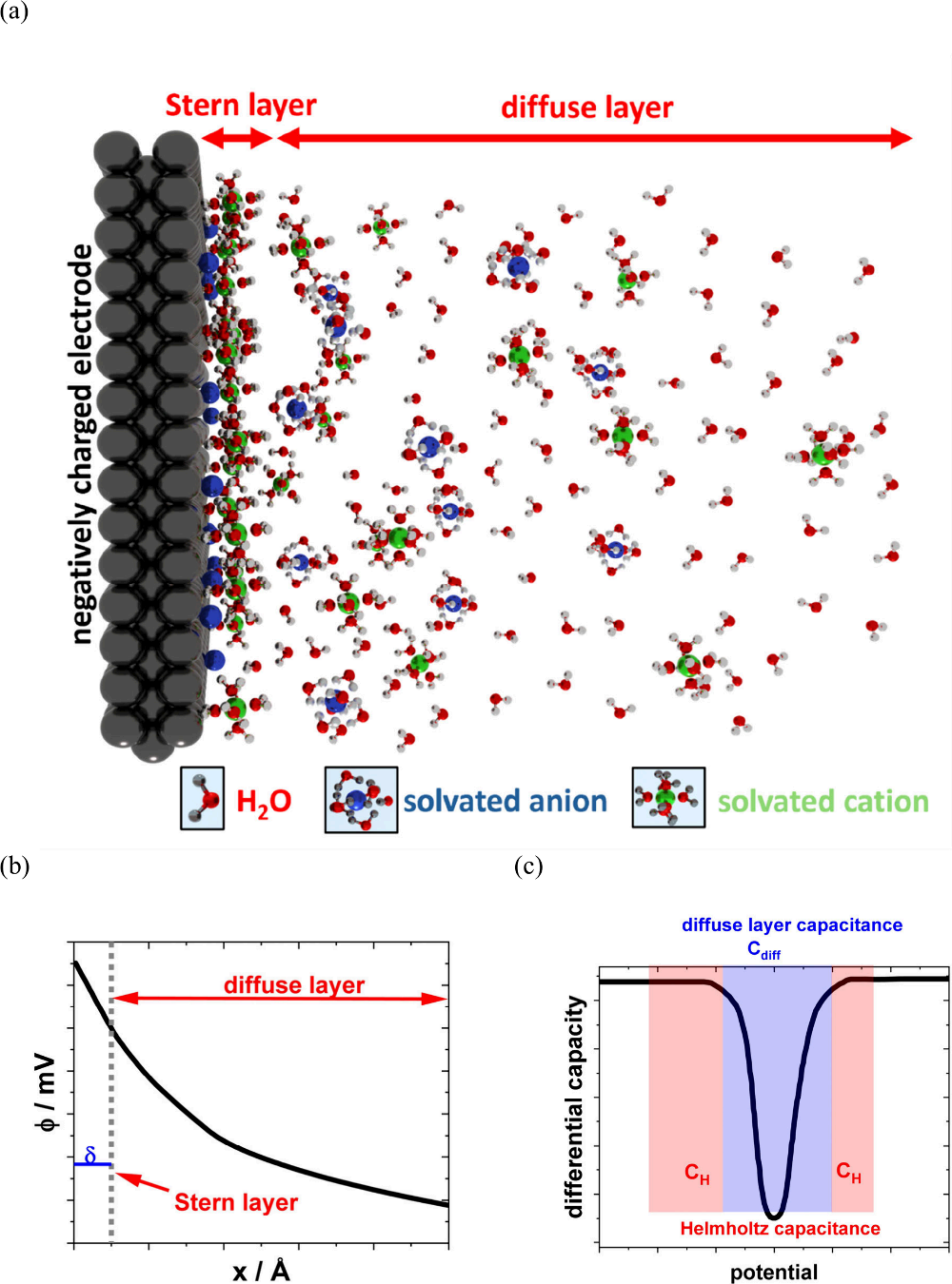
(a) Schematic representation of the EDL present for a negatively charged electrode. The graphic considers the specific adsorption of anions, the Stern layer with adsorbed, solvated cations, and the diffuse layer. (b) Scheme of the potential profile and (c) differential capacitances obtained from the Gouy–Chapman–Stern model in the case of a low electrolyte concentration. (b, c) Adapted with permission from ref (6). Copyright 2001 John Wiley & Sons.

The region for *x* > δ is identified as the diffuse region. Certain simplifications are made when formulating the expressions for the potential profile and differential capacitance within this model. One simplification corresponds to considering only those electrolytes where the cation and anion have matching charges. The potential, ϕ, relative to the bulk solution, can be defined using eq 3. In eqs 3–5, *e* represents the charge of an electron, *T* stands for the temperature, ϕ_δ_ denotes the potential at position δ relative to the bulk solution, *k* is the Boltzmann constant, *z* signifies the charge number, and *n*^0^ is the ion concentration. Furthermore, the parameter κ is described in eq 4. The differential capacitance *C*_d_ is described by eq 5. A schematic illustration of the EDL structure is displayed in Figure 2a for a negatively charged electrode in the case of low-concentration electrolyte solutions. In this schematic, specific adsorption of anions is considered next to the Stern and diffuse layers. However, Figure 2b,c displays schematic representations of the potential profile and differential capacitance profile obtained from the Gouy–Chapman–Stern model.
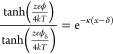
3
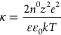
4
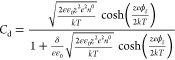
5

Another important parameter related to the theory of solid–liquid interfaces is the potential of zero charge (pzc) at the interface. Frumkin et al. first introduced the concept of pzc of different metal electrodes in 1928.^107^ The electrode surface charge depends on the applied potential. The pzc is the potential at which no excess charge is present on the electrode surface. The charge density of the metal surface is described by eq 6:
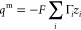
6

In this equation, Γ_*i*_ represents the excess charge of the ionic species *i* at the surface and *z*_*i*_ stands for the charge with the sign of *i*. The quantity of the metal surface charge (*q*^m^) is equivalent to the quantity of the solution side charge (*q*^s^) but with the opposite sign (eq 7).
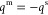
7

At a specific temperature and pressure, the excess charge density on the metal can be expressed as illustrated in eq 8:
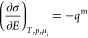
8

Here σ signifies the interfacial tension and is associated with the electrode potential. The pzc is located at a potential at which the derivative  equals 0. For the transition metals, the pzc also depends on the metal work function, which is given by eq 9:
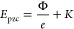
9

In this equation, Φ represents the metal work function, *e* denotes the electron charge, and *K* corresponds to a constant, which depends on the effect of the solvent on the *E*_pzc_ of the electrode. However, it is crucial to note that the correlation between the pzc and the work function, as presented in eq 9, is derived from empirical data analyzed by Trasatti^108^ and, thus, is not entirely fundamental.

For some electrodes, such as Pt, charge transfer occurs through the electrode–electrolyte interface due to the presence of specific adsorption species. Therefore, two types of the pzc need to be distinguished: the potential of zero free charge (pzfc) and the potential of zero total charge (pztc). The pzfc is the potential where the true surface excess charge density equals zero. At the same time, the pztc represents the potential at which the sum of the free, electronic net charge density and the charge density transferred during reversible adsorption processes becomes zero.^109−112^ The interfacial electric field can be governed by the free charge of the electrode surface. The total charge of the electrode surface exhibits a pH dependence due to the adsorption of hydrogen or oxygen on the metal surface.

Furthermore, the properties of solvent molecules are of great importance for the accurate treatment of electrode/electrolyte interfaces. Especially for aqueous electrolytes, the interfacial water structure can directly influence interfacial charge transfer processes, thereby affecting electrocatalytic reactions.^113−115^ To get a deeper understanding of reaction mechanisms, it is essential to investigate the net orientation and the role of structural reorganization of water molecules at the interface. In this regard, the potential of maximum entropy (PME), which is defined as the potential at which the disordering of water structures at the electrode–electrolyte interface reaches the maximum, can be considered a useful experimental descriptor.^109,116−119^ Specifically, the offset between the applied potential and the PME can serve as a measure of the stiffness of the interfacial water layer that can be associated with the energy barrier required to reorganize interfacial water to accommodate charge transfer. At potentials close to the PME, the double-layer region exhibits a higher degree of disorder, making it easier for water to reorient during the interfacial charge transfer. Therefore, electrocatalytic reactions will occur faster due to promoted electron and mass transfer processes. Conversely, when potentials deviate significantly from the PME, the electric field within the double-layer region becomes pronounced. This leads to a more rigid water layer, and the energy barrier for its reorganization steadily increases, thus slowing down the reaction process. As a result, the position of the PME relative to the thermodynamic equilibrium potential of an electrochemical reaction is directly proportional to the reaction kinetics, implying that closer potentials to the PME should lead to larger activity. The PME concept to quantify the rigidity of the EDL is reminiscent of the Marcus theory of electron transfer in which the solvent reorganization energy plays a central role in defining the Gibbs free energy of activation.

**Figure 3:**
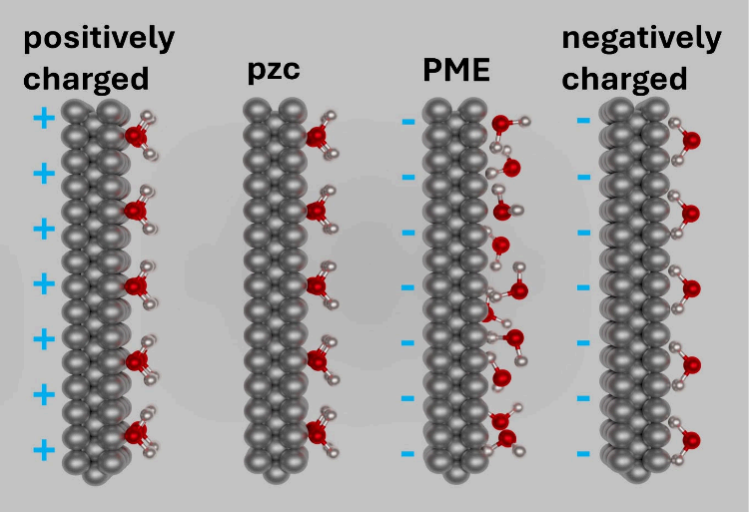
Schematic of water molecules and their orientation according to their dipole characteristic depending on the potential and the surface charge. pzc stands for the potential of zero charge, and PME stands for the potential of maximum entropy. Adapted with permission from ref (131). Copyright 2024 Elsevier.

In most cases, the PME is estimated to be close to the pzc, typically having a slightly more negative value compared to the pzc.^110,120−124^ The difference of both values results from specific interactions between the uncharged electrode surface and the oxygen atom of interfacial water molecules. In this situation, a strong directional bond is formed between the occupied 2p orbitals of oxygen atoms and unoccupied d orbitals at the metal surface.^125^ Therefore, a slightly negative potential is needed to compensate for these specific interactions. A schematic illustration displaying the difference between the pzc and the PME at the microscopic level is displayed in Figure 3, which shows the water molecules and their orientation according to their dipole moment characteristic depending on the potential and the surface charge. From an experimental point of view, the location of the PME can be easily and finely tuned by the modification of the electrode surface^115^ and the alteration of the electrolyte composition or concentration.^126,127^ The PME concept has recently attracted increasing attention since it appears to be directly related to electrocatalytic activity for some systems.^128−130^ For example, Auer et al. established a correlation between the PME and hydrogen evolution reaction (HER) with their findings further suggesting the general validity of the interfacial water reorganization as an activity descriptor for the HER.^114^

#### 2.1.2 Ionic Liquids

Ionic liquids (ILs) are typically compounds composed entirely of ions with a melting point below 100 °C. A unique aspect of ILs that sets them apart from the interfacial structure of aqueous electrolytes is the scarcity of solvent molecules.

Consequently, the ion concentration is above the applicable regime for a dilute solution approximation since treating them as point charges like in the classic Poisson–Boltzmann equation leads, in theory, to absurd concentrations bare of any physical meaning.^132^ By using an approach based on density functional theory and a finite ion volume, Kornyshev proposed a more general equation for the ionic density of cations and anions of the same size.^95,133^
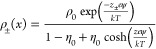
10where η_0_ can be either understood in terms of an ionic density ρ_0_, limited to a maximum (η_0_ = 2ρ_0_/ρ_max_) or as a packing parameter η_0_ = ρ_0_*a*^3^ with the ion volume *a*^3^. Moreover, charge neutrality holds in the bulk (ρ_+_ = ρ_–_ = ρ_0_). The Poisson–Boltzmann behavior can be retrieved from eq 10 for η_0_ → 0, which means that either the ionic concentration approaches a dilute solution or the ion size vanishes.

Furthermore, in contrast to the Gouy–Chapman theory, the concentration of ions near the surface has a saturation maximum that cannot be exceeded, which eq 10 reveals by evaluating certain limits. Even if the polarization is increased (ψ → ±∞), we see that the concentration can only approach ρ_±_(ψ → ±∞) = 2ρ_0_/η_0_ = ρ_max_. Thus, in the case of η_0_ = 1, that is, when the composition at the charged interface consists solely of oppositely charged ions, the maximal ion concentration can only double compared to the bulk, which has been termed the steric effect.^134^

Assuming that the electrode potential is the equivalent to the surface potential φ_s_, the model is also able to calculate the differential capacitance (eq 11) using Gauss’s law:^11,135^
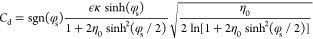
11While the Gouy–Chapman–Stern model predicts monotonically rising capacity for increasing potentials symmetric with respect to the pzc, here the capacitance is either bell- or camel-shaped for highly concentrated solutions, depending on the value of η_0_.^136^

**Figure 4:**
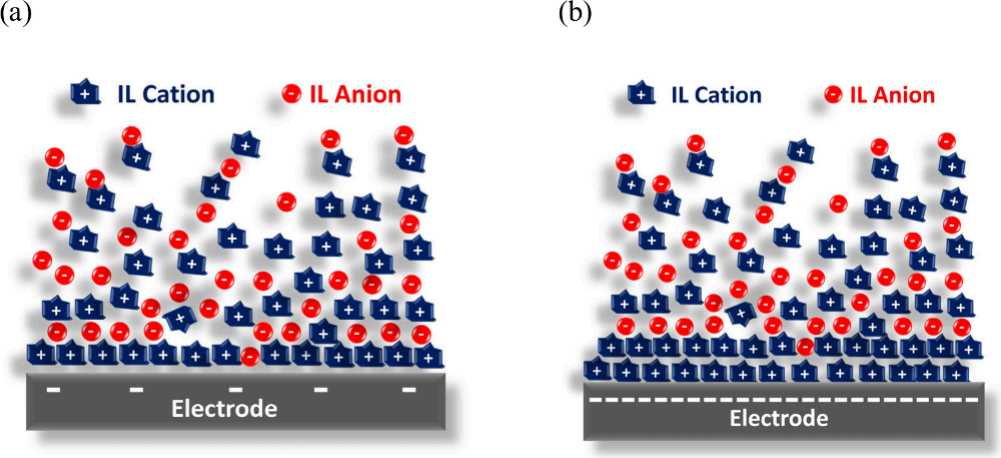
Simplified scheme of the double-layer structure emerging in the case of IL with short alkyl chain at (a) low and (b) high surface charge densities. Adapted from ref (141). Copyright 2021 American Chemical Society.

On a bigger scale, the interfacial structure of an IL can be categorized into three distinct regions: the boundary layer, where ions directly adsorb to the surface; the transition zone, comprised of several transition layers and extending a few nanometers in thickness; and the bulk phase.^96,137−140^ A simplified representation of this complete interfacial structure was reported by Silvester et al.,^141^ who focused on ILs containing a short alkyl chain and homogeneous bulk structure. This representation allows for the differentiation of IL structures based on low or high surface charge density on the electrode. Figure 4a illustrates the case with a low-charge electrode, where a single layer of ions forms to compensate for the electrode’s charge. In contrast, this rigid counterion layer may expand for a highly charged electrode (Figure 4b) due to the larger ion sizes and their delocalized charge. Following this rigid layer in both scenarios, a diffuse region emerges containing different types of ions.

The chemical structures of ILs are also much more diverse due to the presence of various functional groups, which may include both polar and nonpolar side chains. Therefore, a comprehensive and adaptable model for the EDL that can accurately represent the structure of all ILs is still a challenge that is currently the subject of extensive research. For instance, a more sophisticated model, referred to as the primitive model, has been proposed. The primitive model offers a more detailed representation of the EDL by accounting for electrostatic interactions and steric effects arising from the finite size of ions. This model is particularly suitable for incorporation into DFT calculations, providing a more accurate and holistic understanding of the EDL in ILs.^11,142,143^ For instance, in the study of 1-ethyl-3-methylimidazolium tetrafluoroborate (EMImBF_4_) ionic liquid on the Au(111) surface, experiments have demonstrated a decrease in EDL capacity as the potential increases.^144^ The integral capacity is derived in four different ways (from integral free energy, work function, ionic charges, or interfacial dipole moment) and based on DFT data. Still, it hardly changes with respect to the potential. This is the case if the model assumes that there is only a single ion layer at the interface. However, molecular dynamics simulations with a multilayer approach lead then to a dependence on the potential, following the behavior of the experimental data. This shows that the treatment of the double-layer capacitance of ILs as Helmholtz-like leads to an incorrect representation of the structure near the interface.^145^

### 2.2 EDL at Solid–Solid Interfaces

So far, the electrical double layer has been discussed under the assumption that the electrolyte is liquid. Although a few theoretical principles still hold when we compare liquid electrolytes to solid electrolytes, some peculiarities of solid electrolytes deserve a separate brief discussion.

**Figure 5:**
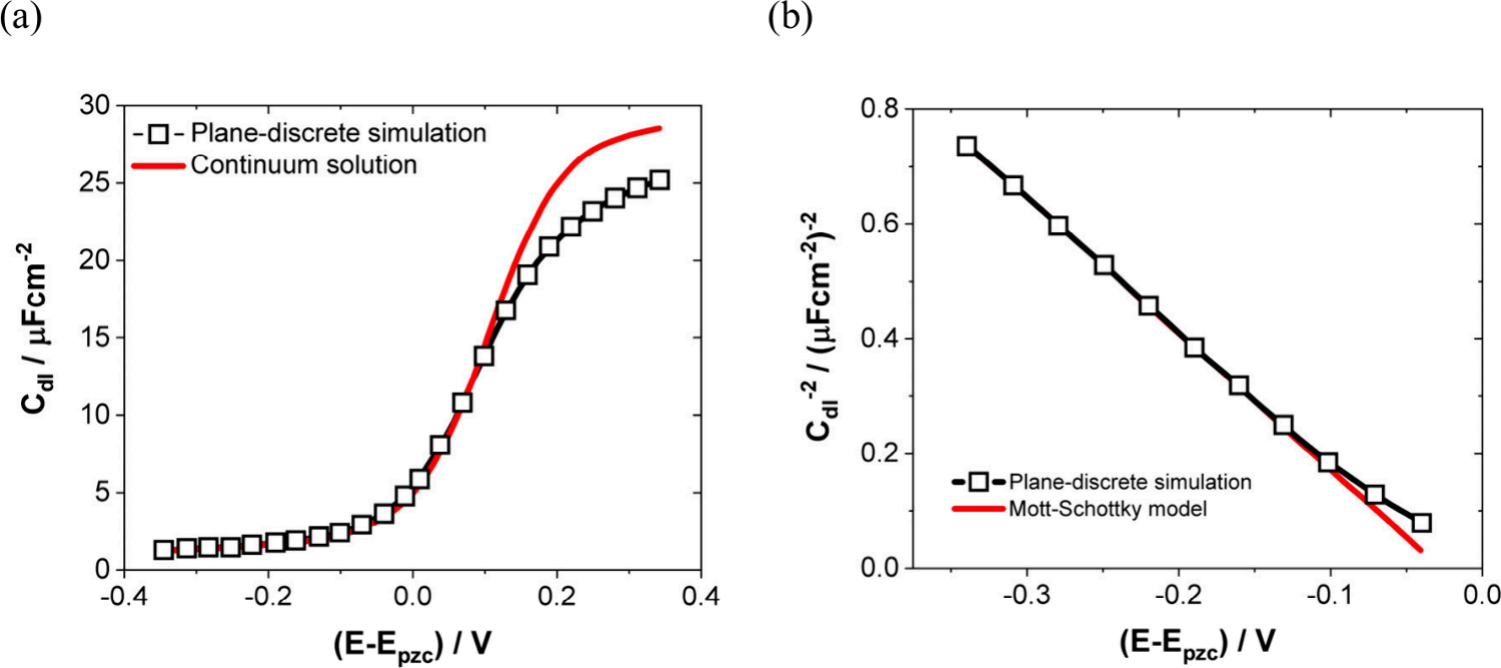
(a) Theoretical double layer capacity at a metal–solid interface as a function of the electrode potential relative to the potential of zero charge (pzc). Calculated under the assumption of a continuous^147^ (red) or plane-discretized electrolyte with a fixed lattice parameter (black). (b) Comparison between the Mott–Schottky model and the potentials from (a) which correspond to a depleted layer. Adapted with permission from ref (146). Copyright 1997 from Elsevier.

In 1997, Armstrong et al.^146^ identified four fundamental distinctions between metal–solid electrolyte and metal–liquid electrolyte interfaces. First, solid electrolytes are composed of at least one static background lattice restricting ions from occupying arbitrary positions. Second, a high charge concentration results in a Debye length comparable to the lattice constant. Third is the absence of solvent molecules. Fourth, the electrolyte adjacent to the metal interface exhibits unconventional properties. Accordingly, the modeling of solid electrolytes at the solid–solid interface needs adaptation to accommodate these distinct characteristics. As a first step, it can be assumed that solid atoms are restricted to planes parallel to the metal surface. This simple restraint to planes illustrates a deviation in the resulting double-layer capacity compared to the Gouy–Chapman–Stern model applied to a continuous solid-state material (Figure 5a). Furthermore, at depletion potentials, the plane-discrete model exhibits behavior closely aligned with the Mott–Schottky model (Figure 5b).

Hence, a discretized treatment of an electrolyte already leads to an improved picture of a solid–metal interface. More recently, Swift et al. highlighted that such a solid-state electrical double layer is governed by point effect concentrations along with the relevant potentials at the interface, e.g., ionic electrochemical potential and electronic structure.^148^ While DFT can predict the defect concentration due to its associations with the solid electrolyte’s electronic structure,^149^ assuming low potentials necessary for the Poisson–Boltzmann equation is not practical during the operation of batteries or fuel cells. Conversely, the Mott–Schottky model is applicable for high potentials as almost all defect sites are either completely vacant or occupied. Two corresponding Boltzmann distributions are necessary to consolidate the two extreme cases within a singular theory. These distributions characterize both the high space charge concentration near the interface and a diffuse part extending into the bulk electrolyte, culminating in the formulation of the Poisson–Fermi–Dirac equation (eq 12).
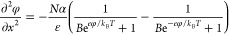
12

In this equation, φ denotes the electrostatic potential, *N*α stands for the saturated defect concentration at the interface, ε represents the dielectric constant, and *B* = α exp[*E*^f^(φ = 0)/*k*_B_*T*] corresponds to a pre-exponential constant stemming from the defect formation energy *E*^f^ at zero potential. To understand the unique ionic structure at solid–solid contacts, we must first understand how the mechanism of ionic conduction influences the formation of the extended double-layer region within the electrolyte near the electrode.

Foremost, ionic conduction in solid-state materials significantly differs from the conduction in liquid electrolytes since, in the latter case, the potential energy profile of mobile ions can be considered flat. In contrast, vacancy diffusion is the most common ionic conduction mechanism in solid electrolytes. The ions need to pass through periodic crystal structures to hop from one vacancy or defect to another, which represents an energetic barrier that separates local minima. The height of those energy barriers strongly influences ion mobility, which in turn determines the ionic conductivity of the solid electrolyte and plays a critical role in the behavior of the EDL at the solid/solid interface.

When a solid-state electrolyte (SSE) and another solid with a different electrochemical potential come into contact, the mobile charge carriers will migrate at the interface so that the potential in the SSE adjusts itself within a certain region to the equilibrium value. The external potential is, hence, effectively screened by a space charge layer (SCL) (depletion layer) at one electrode/electrolyte interface and an oppositely charged SCL (accumulation layer) on the other side. In the liquid case, more and more charges can accumulate at the interface with increasing applied potential. However, similar to ionic liquids, where steric repulsion limits the maximum ion concentration, solid electrolytes also face constraints on charge accumulation. As mentioned, solid electrolytes possess lattice defects that enable ion conduction. Either mobile charge carriers can occupy these defect sites or the sites remain vacant. This implies that exceeding a (local) limit for both the possible maximal and minimal mobile ion concentrations would destabilize the lattice structure.

Apart from the SCLs, narrow, charged layers form directly at the interface, usually with a thickness ranging from 0.3 nm to a few nanometers.^150−152^ This thin double layer can be distinguished from SCLs by its respective dependence on the applied potential. The nanometer-thick double layer adjacent to the interface exhibits characteristic EDL behavior: An increase in potential leads to a proportional increase in double-layer capacitance due to the diminishing distance between the interface and adsorbed species. Compared to that, space charge layers display a contrary trend. Since mobile charge carriers can only occupy defect sites, their concentration cannot exceed the defect concentration. Consequently, when the sites close to the surface are already occupied, with increasing applied bias, the SCL grows laterally into the bulk material, and the associated capacitance decreases due to an increasing SCL thickness.^153−155^

To compare the typically wider SCLs with the diffuse layer for liquid electrolytes, one could characterize it using the Debye length. However, again, the foundational Debye–Hückel theory and the characteristic Debye length only apply to dilute electrolytes with low ion concentrations.^156^ Hence, approaches toward modeling SCLs must be based on other grounds.

To that matter, experimental investigations can be complemented by computational modeling of solid/solid interfaces to better understand various interfacial properties such as stability and ionic conductivity.^157−161^ One key challenge for computational studies is the need to simulate amorphous solid/solid interfaces since inducing structural disorder has emerged as an efficient strategy to enhance ionic conduction. This requires the use of larger simulation cells and longer simulation times. As a result, many computational studies were carried out employing classical force-field molecular dynamics simulations. Those, however, also have some challenges as it is nontrivial to develop reliable interatomic potentials for highly complex amorphous interfaces. In recent years, machine learning techniques have attracted increasing attention as a way to circumvent the limitations of both quantum and classical simulations.^162,163^

## 3 Electrochemical Techniques to Analyze the Electrical Double Layer

Several experimental approaches have emerged to investigate the EDL and fundamentally quantify its characteristics. These methodologies can be divided into two categories. The first category contains techniques aimed at examining the EDL from a microscopic point of view. These methods analyze the EDL structure by studying interfacial water, reaction intermediates, specifically adsorbed ions or molecules, and dynamic EDL processes related to both adsorbing and nonadsorbing species. The second category consists of techniques that explore the EDL from a macroscopic perspective and quantify overall EDL characteristics through global parameters such as the pzc, *C*_dl_, or PME.

We emphasize that this section is intended to offer a general overview of the various techniques and does not delve into detailed theory or experimental procedures. We refer to several key references cited throughout this review for more in-depth information.

### 3.1 Experimental Techniques to Analyze the Microstructure of the Electrical Double Layer

#### 3.1.1 Diffraction/Scattering-Based Techniques

The rapid advancement of diffraction/scattering-based techniques, including X-ray reflectometry (XRR), surface X-ray scattering (SXS), X-ray standing wave (XSW), and surface X-ray diffraction (SXRD), has enabled the characterization of the EDL structure at the electrode/electrolyte interface. XRR probes the detailed EDL structure by measuring reflected X-rays, which are sensitive to electron density changes caused by adsorbed ions in the Stern layer and mobile ions in the diffuse layer.^164,165^ The X-rays used in SXS can penetrate deeply into aqueous solutions facilitating the detection of typical surface reconstructions in electrocatalytic systems, as well as adsorbed ions and atomic positions at surfaces.^166,167^ However, working with samples containing low atomic number (low *Z*) elements poses some challenges, and accurate analysis requires a significant degree of long-range surface order (approximately 5 nm).^168^ Furthermore, specular SXS measurements can be employed to characterize the correlation between surface disorder and the interaction between the electrode surface and the first layer of water molecules, due to its high sensitivity to both the electrolyte and the electrode surface along the surface normal.^42,169^ XSW serves as a practical and straightforward approach for investigating the geometry and positions of adsorbed ions within the EDL structure by determining the bond length distance between a chemically adsorbed/surface atom layer.^170^ Nevertheless, the requirement for nearly perfect single-crystal substrates limits the application of the XSW method. SXRD is particularly sensitive to the top few atomic layers of well-defined crystals and the adsorbed species upon them within the EDL, while transmission surface X-ray diffraction (TSXRD) with high energy X-rays can be employed to investigate surface reconstructions or faceting at the near-surface region.^48,171^ Advanced in situ, real-time XRR can also be employed to explore the potential and time-dependent changes in the EDL where certain adsorption processes occur during electrochemical reactions under operando conditions.

#### 3.1.2 Spectroscopy-Based Techniques

##### 3.1.2.1 Raman Spectroscopy

Raman spectroscopy provides detailed information about the chemical structure, phase, morphology, crystallinity, and molecular interactions of a given sample based on its interaction with light.^172^ The frequency difference between scattered and incident photons, termed the Raman shift (cm^–1^), provides vibrational information about the molecules, including chemical bonds, the electronic environment, and the symmetry of molecules.^173^ Thus, it can be used as a valuable tool to probe the surface reconstruction and the key reaction intermediates at the electrode/electrolyte interface. The advancement of surface-enhanced Raman spectroscopy (SERS) with enhanced sensitivity provides the potential to study the EDL structure of catalysts under in situ conditions by adjusting parameters such as potential or time, particularly on electrode surfaces with rough surfaces and intricate structures.^174^ Shell-isolated nanoparticle-enhanced Raman spectroscopy (SHINERS) also provides enhanced surface sensitivity, allowing for the detailed analysis of well-defined flat single crystals without disturbing the sample surface, which is crucial for precise investigations of surface structures and interactions.^61^

##### 3.1.2.2 X-ray Absorption Spectroscopy (XAS) Based Techniques

X-ray absorption spectroscopy is a technique that probes the local atomic structure and electronic properties of materials through the interaction of X-rays with matter.^175,176^ In other words, the XAS experiment involves recording the absorption coefficient of X-rays, which varies with the energy of the incident X-ray photons.^177^ Generally, XAS is a term that combines various spectroscopic techniques, including extended X-ray absorption fine structure (EXAFS) and X-ray absorption near edge structure (XANES), also known as near edge X-ray absorption fine structure (NEXAFS).^175−177^ The XANES spectrum can be acquired in the interval from a few tens of electronvolts before and after the edge, while the EXAFS spectrum can be measured at higher energies. Besides, XANES focuses on identifying the oxidation state and d-band occupancy, while the higher energy regions of the spectrum (EXAFS) provide information about the local chemical environment surrounding the absorber atom, including coordination number, distance, Debye–Waller factor, and inner potential correction.^176^ Therefore, investigating the EDL, XAS techniques can provide valuable information about adsorbing and nonadsorbing species at the interface.^51,52,178^

##### 3.1.2.3 X-ray Emission Spectroscopy (XES)

XES is an element-specific method that allows for the collection and analysis of specific information regarding the environment coordination structure of a sample, including oxidation states, symmetry, energies, interactions of occupied orbitals, and metal–ligand covalency. Implementing in situ XES within specially designed liquid cells enables the detection of changes in the EDL structure, including the adsorption/deposition of key species, surface morphology alterations on the electrode, and potential-dependent distributions of interfacial molecules.^179^

##### 3.1.2.4 X-ray Photoelectron Spectroscopy (XPS)

XPS allows for the quantitative detection of the surface chemical composition, atom concentrations, and bonding conditions, as well as the detailed electronic structure of a material.^180^ The surface sensitivity emerges due to the small photoelectron escape depth (<10 nm) due to the short path of the emitted photoelectron, making XPS beneficial for probing the EDL structure at the electrode/electrolyte interface, where the extension of the overall EDL in aqueous solution is generally less than 30 nm and the Stern layer is less than 5 nm.^181^

##### 3.1.2.5 Electrochemical Infrared Spectroscopy (EC-IR)

EC-IR, also referred to as electrochemical in situ Fourier transform infrared spectroscopy (EC-FTIR), is a highly sensitive technique that captures in situ vibrational spectra of species adsorbed on metal electrodes.^54^ In the scope of double-layer research, EC-IR facilitates the investigation of charge separation structures through the analysis of surface adsorbates. EC-IR is often combined with other methods to estimate kinetic parameters and prevent misinterpretation of reaction mechanisms.^182^ Fundamental challenges are related to the IR absorption by solution electrolyte species and the partial loss of IR energy during reflection at the electrode surface. In addition to utilizing highly reflective electrodes, enhancing the signal-to-noise ratio in spectra can be achieved by employing weak signal determination methods such as potential or polarization modulation.^183,184^ Two approaches are distinguished in cell design to mitigate the strong absorption by electrolyte species: internal and external reflection configurations. In the external reflection configuration, the electrode sample is positioned close to a light-guiding prism, with a thin electrolyte layer (1–10 μm) ensuring a short path length through the liquid and maximal IR illumination of the sample.^184^ In the internal reflection mode utilizing attenuated total reflection (ATR), a thin metal film deposited on an IR transparent prism serves as the working electrode. The IR beam is focused on the interface from the back of the electrode and is not directed through the solution. Therefore, a thicker liquid layer can be utilized, facilitating free mass transport.^185^

##### 3.1.2.6 Electrical Transport Spectroscopy (ETS)

ETS is used to investigate the electronic properties of materials, particularly in the context of carrier transport. In the scope of double-layer research, the electrical conductivity of ultrafine metallic structures provides information on surface adsorbates. The scattering of conduction electrons induced by surface adsorbates results in a change in resistance, modifying electrical conductivity.^186,187^ By employing a nanodevice based on platinum nanowires and simultaneously conducting cyclic voltammetry, Ding et al.^188^ could monitor dynamic electrochemical interface characteristics between the metallic nanostructures and the electrolyte under varying electrochemical conditions. The ETS approach allows for real-time investigation of surface adsorbates on active catalytic surfaces and thereby provides insights into the structure of the electrochemical double layer.

##### 3.1.2.7 Sum Frequency Generation (SFG)

Sum frequency generation has emerged as a powerful surface-specified method to probe electrochemical interfaces and has been noted for its low detection limits and high interface sensitivity.^189^ At the investigated electrode interface, an incident visible photon and broadband or tunable infrared photons interact with the molecular vibrations of the materials. This interaction constitutes a typical second-order nonlinear process. During vibrational SFG (VSFG) measurements, incident photons can combine their frequencies, emitting a sum frequency photon at the interface with a symmetry-broken structure. The generated sum frequency signal is closely linked to molecular vibrations that resonate with the combined frequency,^189^ allowing for detailed investigations of various molecular orientations and structural details at the electrode/electrolyte interface by adjusting the incident angles or polarization states of the laser beams. In situ VSFG, facilitated by controlling the overpotential, provides the capability to examine the structure of the EDL dynamically.

#### 3.1.3 Scanning Probe Techniques

##### 3.1.3.1 Scanning Tunneling Microscopy (STM)

STM relies on measuring the tunneling current between a sharp metallic tip and a conductive, smooth, and well-ordered sample surface. High-resolution topographic mapping can be obtained by moving an atomically sharp probe either in constant potential mode or in constant height mode across a substrate. Additionally, STM imaging can reveal electronic differences in the sample.^190,191^ For instance, the approach has been promising in investigating the wetting layer of ice on single crystals, revealing molecular structures and periodicities of superstructures at low temperatures.^34,35^

Electrochemical (EC) STM is a configuration that combines STM and a miniaturized electrochemical cell containing a counter, reference, and two working electrodes (STM tip and sample), along with an electrolyte. This setup combination enables the investigation of electrified solid/liquid interfaces under potential control.^192^ Other combinations of STM with techniques such as X-ray photoelectron spectroscopy and Raman spectroscopy allow providing information about the structural, chemical, and electronic properties of materials.^190^ Additionally, classical STM methods enable surface scanning at relatively slow rates, making them suitable for imaging stable systems. However, in the case of dynamic systems where modifications occur over short time intervals, for instance, the diffusion of reactants on the electrode surface, electrodeposition, surface reconstruction, and metal dissolution, STM techniques with faster speeds (20–200 frames per second) have been developed.^193^ Such modified setups are often referred to as video-STM.^191,193^

### 3.2 Experimental Techniques to Macroscopically Quantify the Electrical Double Layer

#### 3.2.1 Cyclic Voltammetry (CV) and Galvanostatic Charge/Discharge (GCD)

CV and GCD methods have been widely employed to investigate electrical properties at solid/liquid interfaces such as the *C*_dl_. For CV-based approaches, the relationship between the capacitance (*C*) and scan rate can be described by eq 13,^66^ where *C* represents the derived capacitance, Δ*V* is the half-cycle potential window, and d*V*/d*t* is the scan rate. Similarly, the average capacitance determined using the GCD method is determined according to eq 14, with *I*_cc_ representing the constant current and d*V*/d*t* the slope of the time–voltage curve.^66,194^
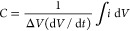
13
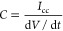
14

It should be noted that the derived capacitances from both equations are only applicable for ideally behaving systems with capacitances remaining constant across different scan rates and charging/discharging speeds.^66^

**Figure 6:**
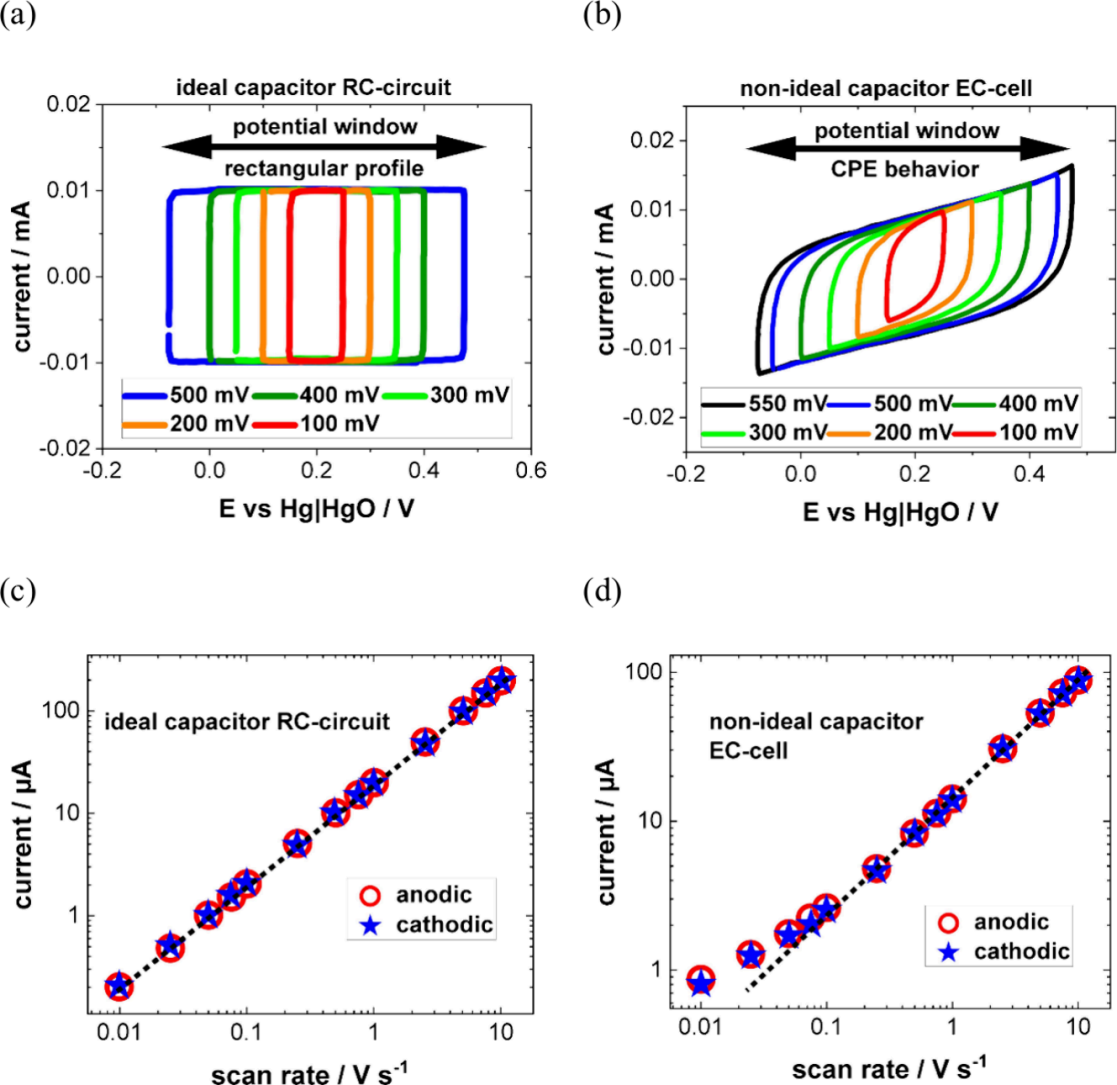
Typical cyclic voltammograms collected for the (a) RC circuit and (b) EC cell with different potential windows. Extracted currents from an anodic scan and a cathodic scan with different scan rates for the (c) RC circuit and (d) EC cell, respectively. (a–d) Adapted with permission from ref (195). Copyright 2021 IOP Publishing Ltd.

For an ideal capacitor, represented by the RC circuit in Figure 6a, the resulting current exhibits a characteristic rectangular profile independently of the selected potential windows and scan rates.^195^ However, in practical systems using electrochemical cells (EC cell), nonideal capacitive behavior (Figure 6b)^195^ arises from irreversible charging/discharging,^196,197^ cell ohmic resistance, solution conductivity, wiring connections, etc.^196,198−200^ As a consequence, the experimentally recorded currents in dependence of the scan rate are higher than the theoretical calculation, as depicted in Figure 6, parts c and d, respectively.^195^ Therefore, it is necessary to select the proper scan rate and potential range for different measuring conditions to more accurately record the derived *C*_dl_.

#### 3.2.2 Electrochemical Impedance Spectroscopy (EIS)

EIS relies on perturbing an electrochemical system at equilibrium or steady state by applying a sinusoidal AC input signal across a broad frequency range and measuring the corresponding AC output.^201,202^ As a frequency domain technique, EIS precisely differentiates and deconvolutes processes at electrode/electrolyte interfaces by their unique time constants.^203,204^ To examine interface processes or properties, a valid impedance spectrum needs to follow requirements such as linearity, causality, and stability, which can be verified by Lissajous plots^205,206^ and Kramers–Kronig relations.^207,208^

Furthermore, an appropriate physical model is required to interpret impedance spectra and access interfacial properties. For EIS measurements within the double-layer potential region of nonideal electrochemical systems, an R-CPE electrical equivalent circuit (EEC) is employed. This EEC employs a constant phase element (CPE) to model the double layer’s response in series with the bulk solution resistance (*R*_s_). The impedance of a CPE element, depicted in eq 15,^209^ includes *C*_dl_^′^, which is proportional to *C*_dl_, and φ, the so-called CPE exponent. The exponent ranges from 0 (purely resistive) to 1 (ideal capacitive behavior), reflecting the frequency dispersion in nonideal capacitors.

Several explanations of the origin of the CPE behavior in double-layer responses have been proposed, including electrolyte concentration, the presence of contaminations,^210,211^ electrode roughness,^211−214^ interfering electrochemical (Faradaic) reactions, and inhomogeneous and fractal properties of electrodes.^211−213,215^ Additionally, the role of 2D and 3D structuring effects within the EDL region, attributed to adsorption processes at the interfaces, has been discussed.^209,216−218^
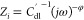
15

**Figure 7:**
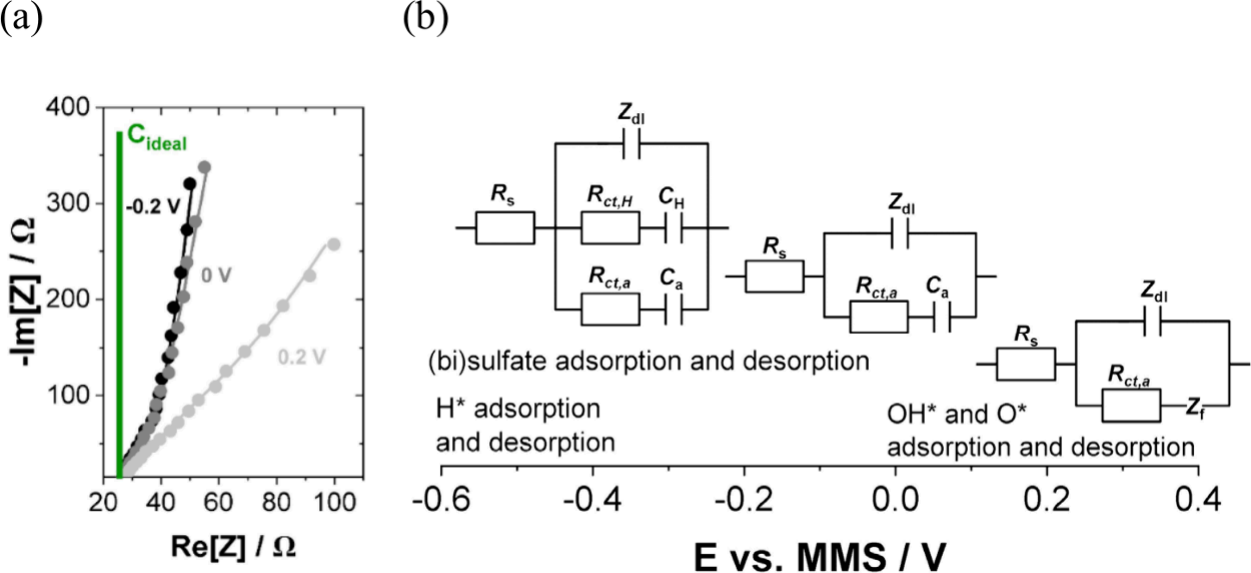
(a) Typical Nyquist plots at the potentials −0.2, 0, and 0.2 V for Pt electrode. (b) Physical models of the EEC for Pt electrode in sulfate media with a wide range of potential windows reported in the literature. Adapted from ref (204). Copyright 2012 American Chemical Society.

For more complex electrochemical processes, the Dolin–Ershler–Randles approach demonstrates EEC models, wherein the double-layer impedance is parallel with additional impedances from electrochemical reactions affected by interface kinetics or mass transport.^209,219−221^ For instance, Figure 7a shows Nyquist plots measured at varying potentials, revealing deviations from the ideal capacitor’s straight spectrum.^204^ Furthermore, Figure 7b demonstrates several physical EEC models for a Pt electrode in sulfate media across a broad range of potentials.^204^

Another intriguing method is dynamic EIS (dEIS), or potentiodynamic impedance spectroscopy (PDEIS),^222,223^ which combines cyclic voltammetry with EIS and involves applying a small alternating potential amplitude during cyclic voltammetry across a selected potential range. However, dEIS may exhibit altered *C*_dl_ current responses and increased charge transfer resistance, yielding results similar to classical EIS at low frequencies.^224−226^ Furthermore, alternating current cyclic voltammetry (AC-CV) provides a similar combined approach that primarily applies a linear potential sweep while simultaneously superimposing a sinusoidal waveform of a fixed frequency to analyze both kinetic and capacitive processes within the electrochemical system.^227,228^ While the combined approach allows for efficient simultaneous acquisition of capacitance information across different frequencies, the data analysis becomes increasingly complex when the system exhibits nonlinear behavior, such as CPE properties.^227,229^

#### 3.2.3 CO Displacement

The CO charge displacement approach has been utilized to determine the pzc^230^ or, to be more specific, the pztc for single-crystal, platinum group metal electrodes.^69,70,111,118,231−234^ Electrochemical CO adsorption at the electrode surface is facilitated through the application of a constant potential, allowing the charge of CO displacement to be approximated as the total charge of the electrode surface. To identify the pztc, the method should be repeated at various potentials or determined from a single potential measurement by integrating the voltammetric current. The pztc is identified as the intercept potential on the charge versus potential curve, where the total charge equals zero. However, the method approaches limitations in distinguishing local contributions to the pztc due to the terrace, step, and kink sites.^232^ The surface sensitivity to the local pztc can be probed by the N_2_O reduction method.^232,235,236^

#### 3.2.4 Scanning Electrochemical Probe Microscopy (SEPM)

SEPM techniques, including scanning electrochemical microscopy (SECM), scanning electrochemical cell microscopy (SECCM), and scanning ion conductance microscopy (SICM), employ an electrochemical tip or probe to investigate localized properties of interfaces.^237−240^

SECM typically employs a metallic disk-shaped electrode encased in an insulating body, allowing resolutions in the micrometer to nanometer range. The current at the SECM tip arises from either a Faradaic or non-Faradaic process occurring at the tip surface, dependent on the distance between the tip and the sample. Consequently, one limitation of SECM is to deconvolute the contributions from topography and reactivity.^241−243^ For double-layer research, applying a sinusoidal potential to the SECM tip enables localized electrochemical impedance spectroscopy (LEIS). Besides the electrolyte contribution and the contribution of the probing tip, the AC response encompasses the local interfacial impedance (usually modeled by a double-layer capacitance or a CPE) and the Faradaic impedance of the surface.^73,244^

SECCM employs a small droplet positioned at the tip of a pipet to enable high-resolution functional imaging and nanoscale electrochemical measurements. In SECCM, the electron transfer reaction occurs directly on the surface under investigation. A single-barrel or double-barrel pipet is filled with electrolytes, and a counter electrode is inserted into each channel. The SECCM tip droplet serves as the confinement for the electrochemical cell. In the double-barrel configuration, a potential is applied between the two counter electrodes to establish the set point, while the sample potential is applied between the sample and the counter electrodes.^240,245,246^ By measuring the charging current when the droplet comes into contact with the surface, SECCM can be employed to determine the pzc.^247^ The procedure for determining the pzc using the microcapillaries in SECCM is based on the immersion method. In this method, a fully discharged electrode is immersed in an electrolyte and the current transient is recorded at various potentials to identify the pzc.^248,249^

SICM employs an electrolyte-filled nanopipet as a scanning probe, utilizing the ionic current flow between an electrode inside the nanopipet and another in the bulk solution or electrochemical cell. A DC or AC potential is applied between the electrodes to generate a surface-sensitive feedback signal. Topographical details of the sample are acquired by scanning the interface in the *x*- and *y*-directions, with fluctuations in the ionic current flow indicating the tip’s position relative to the substrate surface. Similar to AC-SECM, AC-SICM can be utilized to measure the local double-layer capacitance.^75,250^

#### 3.2.5 Laser-Induced Current Transient Method (LICT)

The PME can be experimentally determined by the LICT method or, more broadly, the laser-induced temperature jump (LITJ) technique.^76,251,252^ To ensure reliable measurements at the desired potential value, it is important to hold the potential for a certain time to reach an equilibrium in the system before illuminating the working electrode surface with a laser^253^ or following the procedure as described elsewhere.^110,254^ Essentially, laser pulses of 5–8 ns illuminate the electrode and locally increase the temperature by 15–40 K,^110,128,254−256^ disrupting the interfacial system out of its equilibrium by disordering the water layer for a short time. After this perturbation, a rapid relaxation occurs, and the temperature will decrease.

Since the LICT is conducted potentiostatically, the system response is manifested through sharp current transients. Suppose the electrode surface is negatively charged at the applied potential. In that case, water molecules orient themselves with their hydrogen ends toward the electrode surface, resulting in a negative current transient as the system responds. Conversely, if the electrode surface is positively charged, the oxygen ends of the water molecules align toward the electrode, leading to a positive peak in the current transient.^109,116^ Notably, the polarity of the current transient undergoes a transition, and exactly this transition point corresponds to the PME. It is important to note that specific ion adsorption can affect the results of LITJ/LICT techniques. The influence of sulfate adsorption on interfacial water restructuring has been reported for Pt(111), Au(111), and Ir(111) electrodes.^110,118,256^ For instance, in the case of Ir(111), it was shown that the change in the sign of the transients occurred at much lower potentials in sulfuric electrolytes compared to perchlorate or fluoride ones.^118^ Besides, the potential sweep direction during LITJ/LICT experiments plays an important role in the identification of the PME. For instance, investigations of the interface between room temperature ionic liquids (RTILs) and a Pt(111) surface have shown that solvent restructuring dynamics depend on the applied potential, the potential sweep direction, and the specific cation of the RTIL. Such dependence was demonstrated by potential hysteresis response of the interface on the laser illumination toward potential sweep directions.^257,258^

## 4 Interfacial Water and EDL Structure Investigation

The role of water in aqueous electrolytes and its influence on electrocatalysis has been increasingly recognized in recent years.^259,260^ At the electrode/electrolyte interface, interactions between water and the electrode surface can dramatically influence charge transfer processes, affecting the electrocatalytic reaction activity.^261−263^ To understand how the water structure modifies mechanisms of electrochemical reactions, a comprehensive understanding of its complex structure is mandatory, including knowledge about the net orientation of water molecules, water binding energy, hydrogen bonding network, and its interaction with cations and anions.^264−267^ From a theoretical point of view, the studies of interfacial water structure face serious challenges since it can be affected by a multitude of interdependent factors such as the nature of electrode surfaces, electrolyte composition and concentration, electrode potentials, and temperature.^127,203,268−271^ To discuss this complexity, we next guide the reader through some published experimental, theoretical, and combined theory–experiment studies and highlight the main milestones in interfacial water research.

### 4.1 Experimental Studies of Water/Metal Interfaces

The interfacial water structure is known to depend strongly on the applied electrode potential. Therefore, examining interfacial properties at the molecular level is critical as a function of external potential. In this regard, several experimental techniques have proved to be extremely helpful, including X-ray spectroscopy,^52,272^ X-ray reflectivity and diffraction,^44,45^ and vibration spectroscopies like infrared absorption spectroscopy,^273,274^ Raman spectroscopy,^60,61,275^ and sum frequency generation spectroscopy.^56,58,189^

Studies in the 1990s focused on the OH stretch vibrations of water molecules with different bulk pHs using infrared visible sum frequency generation at fused quartz/water interfaces.^57^ The structure of interfacial water and the net orientation of water molecules strongly depend on the electrostatic interaction and hydrogen bonding of molecules with the electrode surface. In solutions with different pH values, the entropic changes of the water structure were observed through a transformation in water orientation, particularly at high pH values. Under these conditions, the electrode surface can be strongly electrified, forcing water to form highly ordered structures. In 1994, Toney et al. used surface X-ray scattering to explore the water density distribution perpendicular to the Ag(111)/0.1M NaF interface at +0.52 and −0.23 V versus the pzc of the electrode.^42^ The results suggest that the degree of disorder and the distance between the electrode surface and the first layer of water molecules are potential-dependent. For negative/positive potential, water has an oxygen-up/oxygen-down average molecular orientation. Surprisingly, in contrast to the bulk water, the first water layer at the interface contains a markedly increased number of water molecules. Such a significant increase in the water molecule amount leads to disrupted hydrogen bonding. In later years, further studies of the EDL structure were conducted using different techniques.^276−278^ The location of ions within the Helmholtz layer and the in situ partitioning between the Helmholtz and diffuse layers at the rutile (110)/water interface were investigated through small-period XSW.^50^ The comparison of ex situ and in situ measurements provided a significantly more detailed picture of the double-layer structure. Besides, scanning tunneling microscopy measurements to investigate the EDL^36^ were also carried out by several groups. For example, by using STM, Hiesgen et al. separated the influence of the tunneling barrier from that of the Schottky barrier at the semiconductor/electrolyte interface. The immersion of the tip into the electrochemical double layer allows one to explore the effect of the tip potential on the tunneling barrier height in the semiconductor, which directly facilitates our understanding of the EDL structure.^279,280^ Hugelmann et al.^37^ also found that the tunneling current exponentially decays with the gap width of an oscillation period of 0.35 nm. This period can be traced back to the spacing of water molecules in the Helmholtz layer of the solid/liquid interface, revealed by theoretical predictions. Simeone et al.^38^ further investigated the tunneling probability and detected a nonoscillatory behavior of the effective barrier height near the pzc.

Recently, Ali et al. investigated dissociated water and estimated the quantity of OH^–^ anions to predict the length of the electric double layer.^281^ Through X-ray spectroscopy analysis of the OH^–^, they concluded that thick electric double layers are formed, consisting of negatively charged diffuse layers over 0.44 nm thick and approximately 0.35 nm wide charge-balanced Stern layers. In 2023, Piontek et al.^282^ probed the orientation and hydrogen bonding strength of water in the interfacial region by employing surface-specific vibrational SFG spectroscopy. All these studies underscore the progress in spectroscopic characterization of interfacial structures.

**Figure 8:**
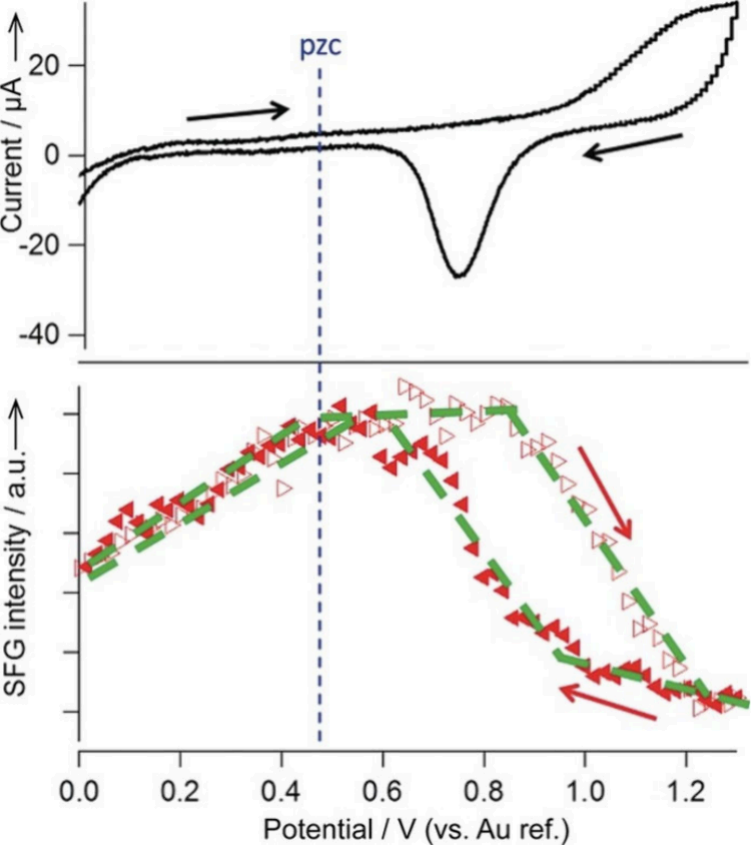
Au electrode CV in 1 M HClO_4_ solution with a scan rate of 5 mV s^–1^ VSF intensity of the free-OH peak as a function of potential during the same scan. Red arrows indicate the scan direction (positive or negative); the dashed blue line at 0.5 V is the pzc. Reprinted with permission from ref (58). Copyright 2017 Wiley-VCH-Verlag GmbH & Co. KGaA.

More advanced techniques and a more accurate understanding of local water structure at the electrode/electrolyte interface with changing potential are required to quantitatively describe reaction mechanisms. An outstanding achievement was reported by Tong et al.,^58^ who investigated the potential-dependent structure and net orientation of interfacial water using interface-specific vibrational sum frequency (VSF) spectroscopy in a thin film electrochemical cell. The in situ CV curve and the integrated intensity of the narrow VSF feature are depicted in Figure 8. The pzc of the Au electrode is located near 0.5 V. An increase in VSF intensity from 0.0 to 0.5 V in the positive scan direction can be seen. In contrast, a plateau from 0.5 to 0.9 V and a significant decrease with the onset of oxidation at potentials more positive than 0.9 V are observed. In the negative scan direction, with dissolving the Au oxide, a significant increase in intensity at potentials positive of the pzc is observed. At potentials negative of the pzc (0–0.5 V), the change of intensity is similar to that in the positive scan direction. These results indicate that interfacial water molecules on the Au electrode surface tend to orient themselves, pointing away from/toward the electrode surface owing to the interaction between water’s permanent dipole and the surface field. Both OH groups would not be perpendicular to the surface at the negatively charged electrode surface. As the potential increases toward the pzc, a free OH perpendicular to the surface is expected to occur, and the VSF intensity will be enhanced. Similarly, at the potential positive of the pzc, the VSF signal decreases again. In addition, Tong et al. claimed that the density of the interfacial water can be increased when the electrode surface charge changes from negative to positive. The plateau between 0.5 and 0.9 V indicates the compensation of orientation and density effects. Therefore, it is clear that the interfacial water structure strongly depends on the nature of the electrode.

In conclusion, with the help of various advanced techniques, not only the actual structure of the interfacial water layer at the solid/liquid interface can be directly probed but also the spacing of the interfacial water layers can be predicted—this opens a new avenue for the study and understanding of electrocatalysis.

### 4.2 Ab Initio Simulations of Water/Metal Interfaces

Properties of water/solid interfaces have also been thoroughly addressed using DFT calculations. Prior computational investigations of interactions between water and flat metal substrates have served as a great source of fundamental atomic-level insights.^83,86,283−285^ It is important to point out that the strengths of water–water and water–metal interactions are comparable.^286,287^ This leads to the delicate balance between these competing interactions, even at seemingly simple flat surfaces, that needs to be resolved in simulations. As a first step, first-principles studies have focused on elucidating the structure of adsorbed single water molecules, small clusters, and ice-like water layers primarily utilizing static DFT calculations.^86,284^ Despite obvious limitations of such zero-temperature calculations, they provided valuable insights into various interfacial properties such as the atomic structure, the number and strength of H-bonds, the modes of water adsorption (associative versus dissociative), and the redistribution of electron density. DFT simulations were also instrumental in helping to rationalize high-resolution STM images of water/solid interfaces.^288−291^ For example, the ubiquitously used bilayer model^292^ was seriously questioned as DFT results^293^ for Pt(111) indicated that the formation of mixed OH and water species at the interface is energetically more favorable than any undissociated bilayer structure.

Later, a growing number of investigations started employing ab initio molecular dynamics (AIMD) simulations to model interfacial liquid water at ambient temperatures.^83,294−299^ Such AIMD-based simulations enable insights into the dynamic nature and entropic effects of interfacial water. These simulations, however, require sampling of long AIMD trajectories to obtain reliable statistical averages. In addition, modeling results may strongly depend on the exchange–correlation functional employed in DFT calculations that require careful comparison with experiments.^300^ For example, the commonly used PBE functional leads to overstructured water due to the lack of dispersive interactions in GGA functionals.^294,300,301^ To partially remedy this situation, approaches such as semiempirical dispersion corrections (e.g., PBE-D3)^302,303^ and van der Waals functionals (e.g., vdW-DF)^304,305^ were introduced.

The next step toward a more realistic understanding of electrochemical interfaces is to scrutinize their behavior as a function of electrode potential.^83,306−310^ When the charge on the electrode is modified, EDL properties can change drastically due to the restructuring of water layers, migration and adsorption of electrolytic ions, and ongoing electrochemical reactions that add substantial complexity to modeling studies. Theoretically, investigations of even pure liquid water at electrified interfaces represent significant challenges as there is no generally accepted approach to control electrode potential in periodic DFT simulations. One method to vary electrode potential in simulations is to insert atoms into the water region that will donate/accept electron density from the electrode, subsequently becoming either cations (e.g., alkali metals) or anions (e.g., halogens), respectively. This charge redistribution will thus modify the Fermi energy of the electrode, affecting the electrode potential, which can be estimated by monitoring the electrode’s work function. One exemplary study following this method is the AIMD simulations of the Au(111)/water interface.^311^ In this work, the electrode potential was modified (negatively charged) by varying the number of Na atoms (between zero and five) in the water region. As can be seen from Figure 10d, as the applied potential decreases relative to the pzc, water molecules reorient from a parallel (region I) to an H-down (region II) and then even to a two-H-down configuration (region III) at very low potentials. As a result of this process, the number of H-bond donors decreases being correlated with the experimentally measured Raman OH frequencies.

An alternative way to modify the electrode potential is to add or remove electrons from the system directly. In periodic simulations, the extra charge must be compensated by the corresponding countercharge, e.g., via introducing a neutralizing background. An AIMD study of the same Au(111)/water employing this approach has resulted in qualitatively similar insights into water reorientation as a function of applied potential.^409^ To control the applied potential, several approaches were proposed. In 2001, Lozovoi et al. introduced a theoretical framework for ab initio supercell simulations of electrochemical interfaces under a fixed preset chemical potential.^310^ Within this computational scheme, the electroneutrality of the system is restored by allowing an exchange of electrons between the periodic slab and a reference electrode. A few years later, an alternative scheme was proposed by Otani et al., allowing one to model electrolyte/electrode interfaces with fixed excess charge.^312^ Subsequently, in the same research group, first-principles molecular dynamics simulations of the metal/electrolyte interface were carried out at a constant electrode potential.^307^ In the proposed potentiostat scheme, the system was allowed to exchange electrons with an external reservoir at a fixed potential, decoupling the charge dynamics from the electronic structure calculations. In another theoretical study, an approach based on constant Fermi-level molecular dynamics referencing the electrode potential to the valence band maximum of liquid water to align the potential to the SHE scale was put forward.^306^ Computational studies, however, also need to fully account for the dependence of *C*_dl_ on electrode structure and composition, as well as electrolyte chemistry, as alluded to below.

It should also be mentioned that, in recent years, theoretical techniques based on machine learning (ML) interatomic potentials have become widely available as an alternative to AIMD simulations.^313−317^ While DFT-based molecular dynamics simulations provide rather accurate descriptions of electrochemical interfaces, they suffer from two major limitations: limited length and time scales. ML techniques offer several advantages, including accuracy comparable to DFT calculations on which the training was performed, fast evaluation times, and linear scaling with the number of atoms (while DFT scales cubically).

In 2007, Behler and Parrinello proposed^318^ a generalized neural network representation of high-dimensional potential energy surfaces as an alternative to computationally demanding DFT calculations. The method provides the energy and forces as a function of all atomic positions and is several orders of magnitude faster than DFT. In this approach, the atomic coordinates are first transformed into feature vectors, known as atomic fingerprints or symmetry functions. These atomic fingerprints distinguish the local environment around each atom due to its neighboring atoms. A neural network for each atom type takes the descriptor as the input and gives the atomic energy as the output. The total energy of the system is calculated as the sum of all atomic energies. In this approach, the energy is a unique function of atomic positions.

Previously, Behler–Parrinello neural network potentials (NNPs) were successfully employed to study water/metal interfaces. For example, the atomic structure of the water/Pt(111) interface was examined using NNPs at room temperature.^319^ It was demonstrated that the system exhibits a dynamically changing, semiordered interfacial structure, where the water molecules in the primary adsorption layer are distributed homogeneously across the interface, forming frequent hydrogen bonds to water molecules in the secondary adsorption layer. It is important to note that the conclusions obtained are beyond the time scales that are accessible to AIMD. In another study, a variety of structural and dynamic properties of interfacial water at the Cu(111), Cu(110), and Cu(100) surfaces were examined using NNPs.^317^ In this work, a very high precision was achieved with an overall root-mean-square error (RMSE) of only 0.9 meV/atom for the energies and 66.5 meV/bohr for the force components enabling simulations with essentially first-principles accuracy.

Overall, well-developed NNPs can be far superior to conventional AIMD simulations of electrochemical interfaces in system size and simulation time while simultaneously providing comparable accuracy. However, it remains to be seen how such ML approaches can be adapted to perform simulations under electrode potential control.

### 4.3 Combination of Experimental and Theoretical Methods

In the previous two sections, we discussed some important experimental and theoretical studies of water/electrode interfaces separately. In this section, we aim to bring to the fore a few representative investigations in which experiments and simulations were combined to rationalize the obtained data. Most such studies have been conducted on well-defined noble-metal-based systems such as gold and platinum, which is the emphasis of this section.

#### 4.3.1 Gold

**Figure 9:**
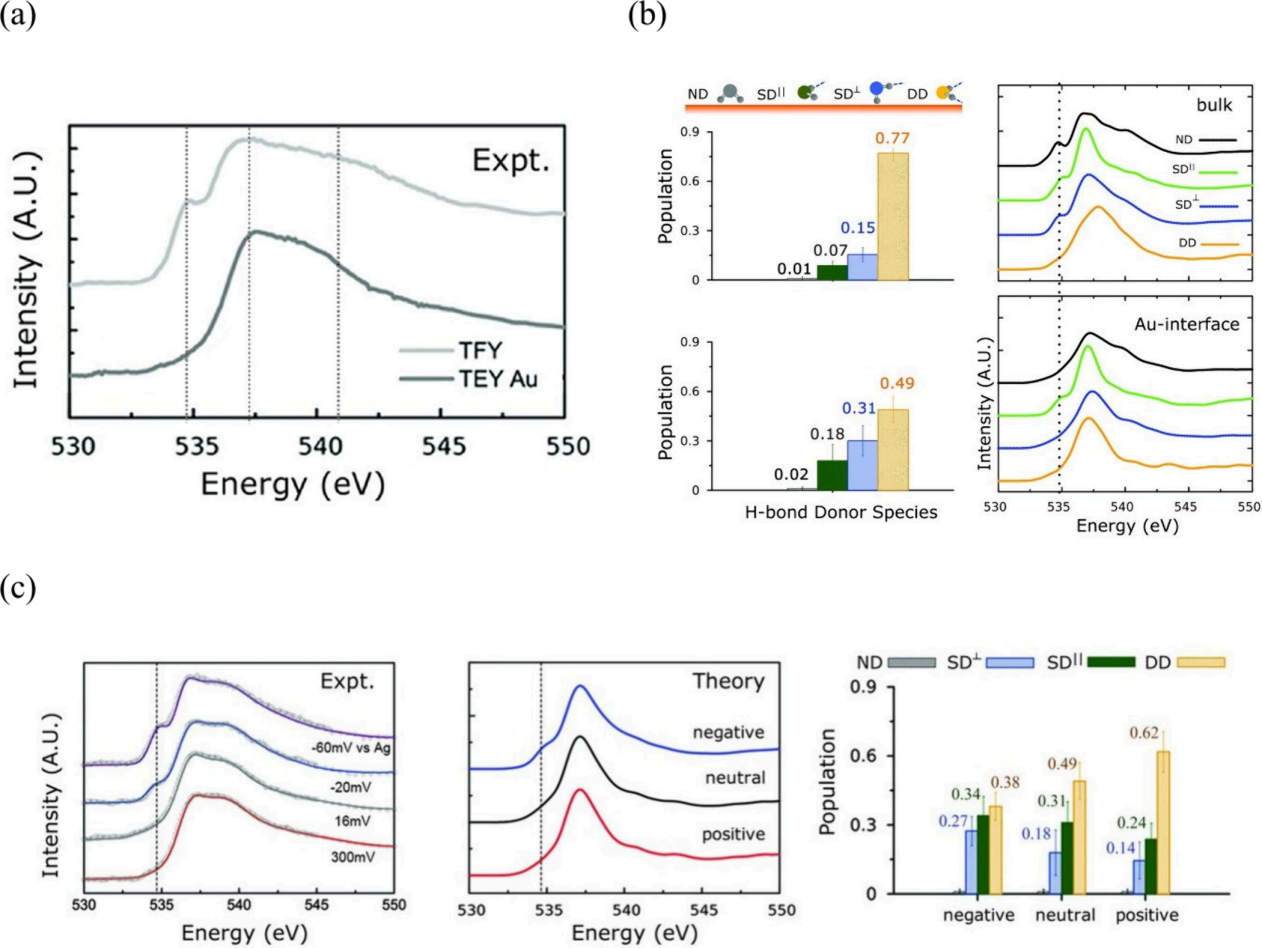
(a) O K-edge spectra obtained by XAS with the two different TFY and TEY detection modes, clearly highlighting three distinct edges. (b) Water molecule population in the bulk electrolyte and at the water/Au interface determined through AIMD. The right subfigure presents the theoretically calculated XAS spectra. Differently oriented water molecules are considered, including nondonor (ND), double donor (DD), and single donor (SD) in parallel (SD^∥^) and perpendicular (SD^⊥^) orientations to the surface. The left subfigure displays the populations of the differently oriented water molecules, respectively. (c) Comparison of experimentally and theoretically XAS spectra for various potentials. The experimentally provided potentials are related to an Ag quasi-reference electrode. The third subfigure in (c) highlights, similar to the left subfigures in (b), the population of H-bonded water molecules. Reprinted with permission from ref (320). Copyright 2014 AAAS.

We start this section by highlighting a combined theory–experiment investigation aimed at exploring the molecular structure of interfacial water on gold electrodes.^320^ The water (10 μm NaCl)/Au interface was examined using both XAS and AIMD simulations. XAS experiments revealed differences between the total fluorescence yield (TFY) and total electron yield (TEY) detection modes (Figure 9a), indicating distinct bulk and interfacial properties of water. That information can be gained from the three categorizations of the O K-edge spectra, including pre-edge (∼535 eV), main-edge (∼537 eV), and post-edge (∼540 eV), which provide information on the nature of the respective hydrogen bonds. The absence of the pre-edge feature in TEY mode suggested a low concentration of unsaturated donor hydrogen bonds at the interfacial region.^52,272,321^ However, AIMD simulations using strained-occupancy DFT within the excited electron and CORE-HOLE (XCH)^321−326^ have contradicted those results, indicating an increased concentration of unsaturated hydrogen bonds at the interface. As seen in Figure 9b, AIMD allows for the differentiation of water orientations and hydrogen bonding types, including nondonor (ND), double donor (DD), and single donor (SD) in parallel (SD^∥^) and perpendicular (SD^⊥^) orientations to the surface. Calculated XAS spectra highlight similarities between SD^∥^ species at the interface and SD molecules in bulk water, whereas interfacial SD^⊥^ species lack the pre-edge feature. Additional experimental and theoretical considerations confirmed that the general pre-edge suppression arises due to an electronic effect from the interaction between the core-excited state and the gold’s electronic structure. The calculated population of differently oriented water molecules in bulk and at the interface reveals an increase of 22% of broken H-bond molecules at the interface. The study further explored the effect of electric fields on interfacial water structure, highlighting alterations in hydrogen bonding dependent on the potential (Figure 9c). As the potential decreases relative to the pzc, most water species transition from DD to SD molecules, particularly in parallel orientation, which is indicated by the emerging pre-edge feature.

This study by Velasco-Velez et al.^320^ provided critical molecular insights into the structural and electronic properties of interfacial water at Au electrodes through a combination of experimental and theoretical tools.

**Figure 10:**
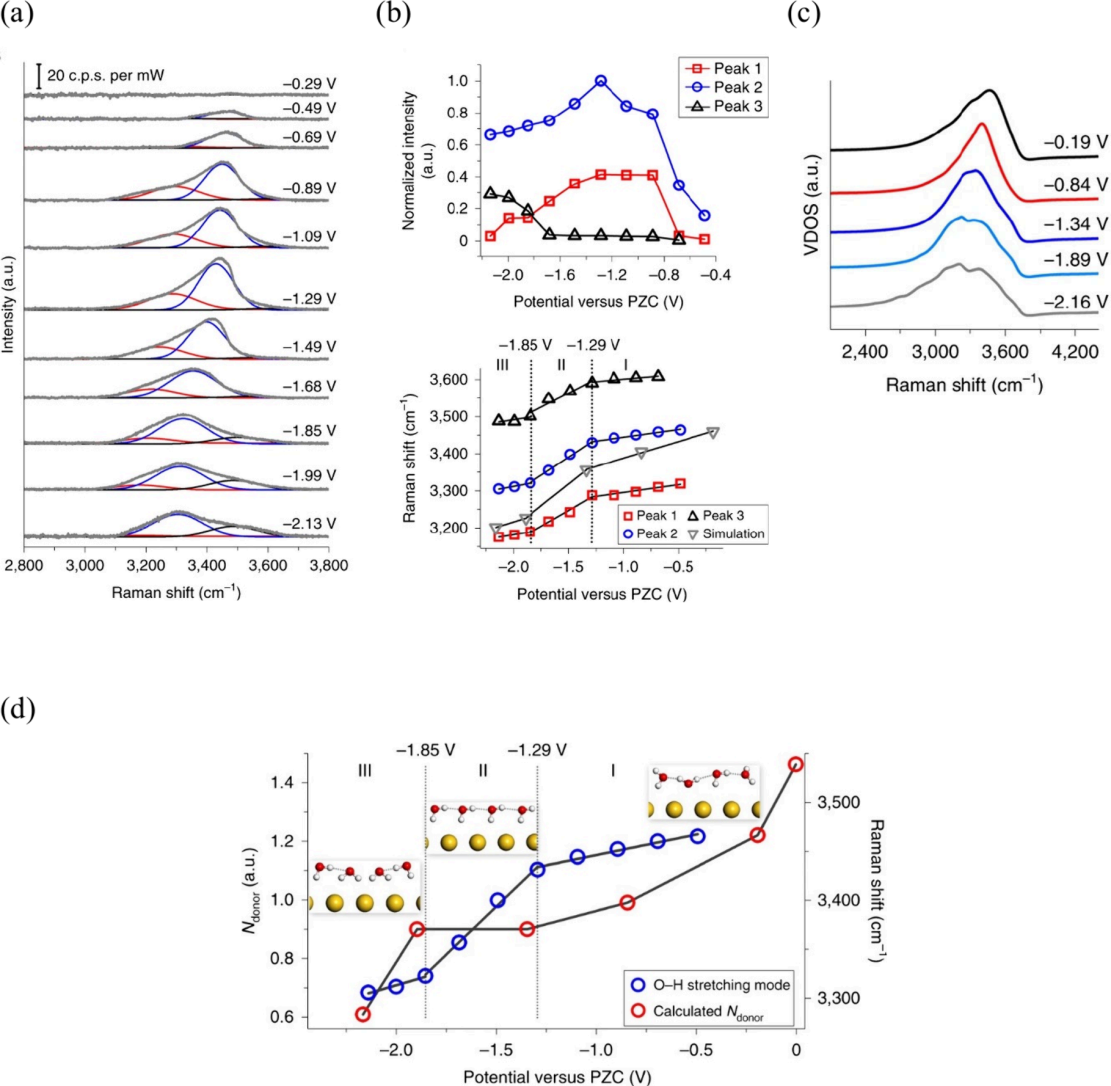
(a) O–H stretching mode of the EC-Raman spectra measured for Au(111) in 0.1 M Na_2_SO_4_ with three fitted Gaussians in blue, red, and black. (b) The intensities of the fitted Gaussians in (a) and their dependence on the potential are shown in the top subfigure. The bottom subfigure displays the experimental and calculated frequencies of the OH stretching mode. (c) Results of the VDOS calculation of different potentials. (d) Number of H-bond donors (red circles) derived from AIMD simulations of the Au(111)/water interface and experimental Raman O–H frequencies (blue circles) as a function of applied potential relative to the pzc. Reprinted with permission from ref (311). Copyright 2019 Springer Nature.

Next, we highlight the work by Li et al.,^311^ who identified potential-dependent changes in the O–H stretching mode at the water (0.1 M Na_2_SO_4_)/Au(111) interface (Figure 10a), distinguishing three distinct types of water configuration (dangling O–H bonds, trihedral, and tetrahedral). While the normalized intensities of the respective peaks (Figure 10b) suggest trihedrally coordinated water as the predominant water structure along different potentials, the potential dependency of the O–H stretching mode frequency reveals a shift toward more anodic potentials and shows two transition points at −1.85 and −1.29 V. To provide insights into these transition points, complementary AIMD simulations were undertaken. First, velocity density of states (VDOS) calculations at different potentials (Figure 10c) were performed confirming the validity of the employed model. Second, simulations enabled the study of water molecule orientation as a function of the electrode potential by calculating hydrogen bond donors (*N*_donor_). Figure 10d displays the *N*_donor_ trend alongside the observed experimental frequencies of the O–H stretching mode as a function of applied potential bias. As the electrode potential is lowered, the decrease in *N*_donor_ (region I) signifies a transition from a parallel water structure to a one-H-down configuration, where the O–H plane aligns parallel to the surface, enhancing hydrogen bond network formation among neighboring water molecules. The observation of trihedrally coordinated water is consistent with the outcomes of experimental results. At more cathodic potentials (region II), the value of *N*_donor_ remains relatively stable, suggesting consistent interfacial water structure. In region III, a partial transition of the aforementioned “one-H-down” toward a “two-H-down” structure occurs. The potential −2.16 V is highlighted, as the *N*_donor_ value suggests that both configurations coexist.

**Figure 11:**
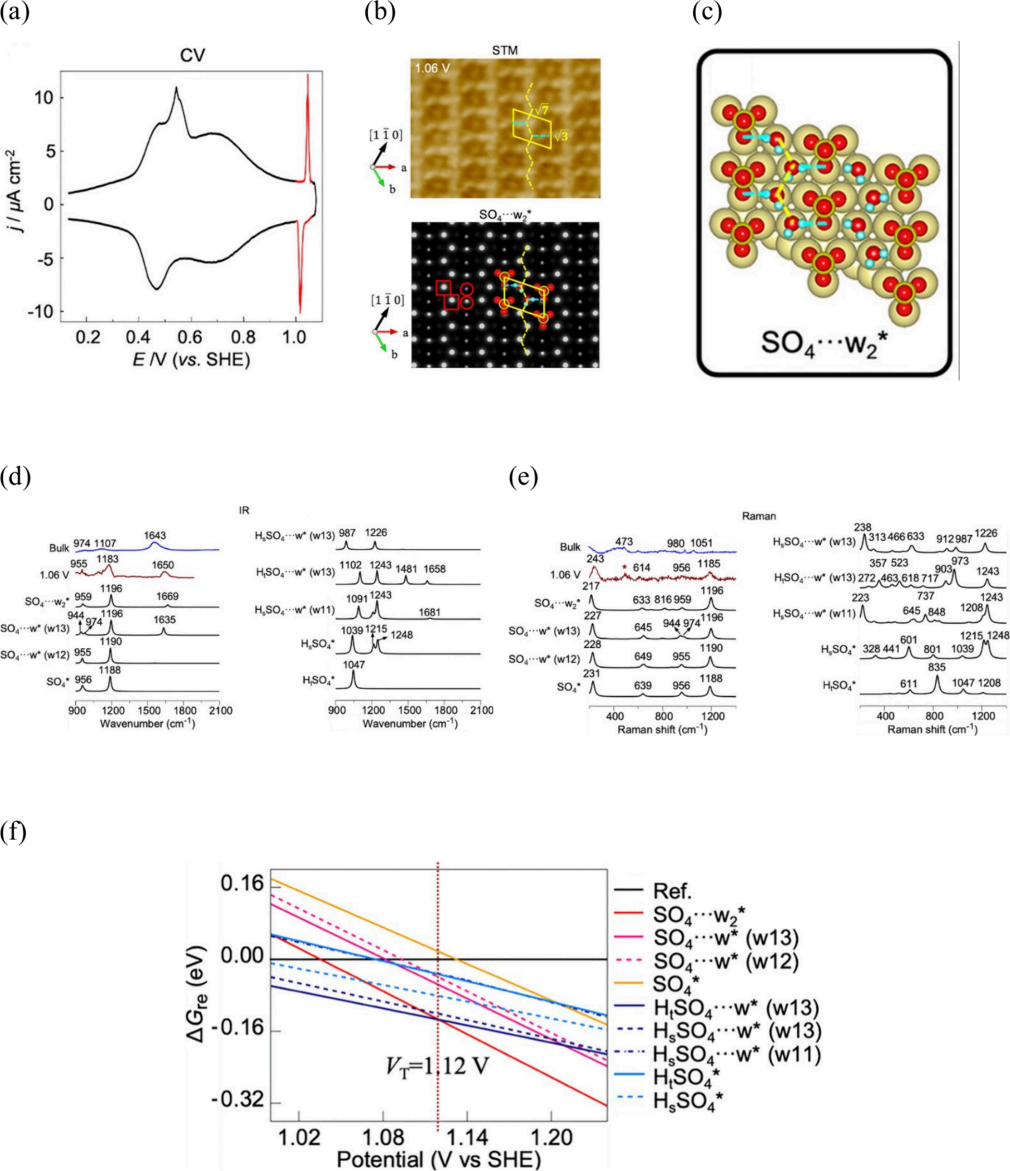
(a) CV of Au(111) measured in 0.1 M H_2_SO_4_ with a scan rate of 20 mV/s. The current spikes at ∼1.06 V vs SHE are highlighted in red. (b) HR-STM image at 1.06 V vs SHE and calculated STM image including the Au(111)(√3 × √7)-(SO_4_···w2) configuration. (c) Schematic of the Au(111)(√3 × √7)-(SO_4_···w2) configuration. Experimental (red and blue) and calculated (black) (d) EC-IR and (e) EC-Raman spectra. Experimental IR spectra were originally published by Ataka et al.;^327^ however, Fang et al.^62^ reproduced the data with their permission. Experimental spectra were recorded for bulk electrolytes and at the interface at 1.06 V. The calculated spectra include different configurations of sulfate and bisulfate anions. (f) Free energy dependence on the potential ranging from 1 to 1.24 V vs SHE for different Au(111)(√3 × √7) configurations. The black reference line corresponds to the (HSO_4_^–^(aq) + 3H_2_O(aq) + ∗) state. Reprinted with permission from ref (62). Copyright 2020 American Chemical Society.

Both studies highlighted above were conducted under circumneutral pH conditions employing either 10 μm NaCl or 0.1 M Na_2_SO_4_. However, as will be shown in the following, the water structure at the Au(111) interface can change drastically in an acidic environment. The work from Fang et al.^62^ reports the results of CV measurements under such acidic conditions (0.1 M H_2_SO_4_, see Figure 11a), providing insights into the adsorption configuration and atomistic structure responsible for the emerging current spikes at 1.06 V vs SHE. In this study, Fang et al. utilized a combination of techniques such as high resolution (HR) EC-STM, EC-IR, EC-Raman spectroscopy, and DFT simulations. Figure 11b displays HR-STM recorded at the respective 1.06 V potential and DFT-simulated EC-STM images, both revealing an Au(111)(√3 × √7)-(SO_4_···w2) adstructure. Figure 11c shows that each unit cell is comprised of a sulfate ion (SO_4_*) and two water molecules (w2*), forming a symmetric hydrogen bonding network with configurations of water–sulfate–water [w···SO_4_···w]_*n*_ and water–water [w···w···]_*n*_ chains. For the EC-IR analysis, the authors refer to the experimental work from Ataka et al.,^327^ with Figure 11d showcasing the EC-IR spectra in bulk electrolytes and at the interface (1.06 V) depicted by blue and red curves, respectively. These spectra reveal three main bands corresponding to different vibrational modes of sulfate ions and water molecules. Despite the experimental data, the adstructure could not be conclusively determined. Therefore, DFT simulations of the EC-IR spectra for various proposed configurations were carried out. As can be seen in Figure 11d, the Au(111)(√3 × √7)-(SO_4_···w2) structure aligns closely with the experimental results. Similarly (Figure 11e), the authors analyzed the Raman spectroscopy data, comparing differences between bulk and interfacial spectra (1.06 V) with the calculated Raman spectra. Drastic differences occur between the bulk and interface-specific spectra, revealing distinct vibrational bands and suggesting the presence of sulfate ions (SO_4_*) at the interface. The authors concluded that both the EC-Raman and EC-IR analyses support the identification of the Au(111)(√3 × √7)-(SO_4_···w2) structure as the predominant adstructure at high potentials. These findings were further validated by computing the reaction free energies for various proposed configurations as a function of the electrode potential, as shown in Figure 11f. Indeed, above the transition potential at 1.12 V, the previously identified Au(111)(√3 × √7)-(SO_4_···w2) configuration emerges as the most stable interfacial structure. Below the transition point, the H_t_SO_4_···w* (w13) structure seems to be most stable, while experimental results (not shown in this section) identified a H_s_SO_4_···w* (w13) structure. However, from the reaction free energy diagram, the H_s_SO_4_···w* (w13) configuration exhibits only marginally smaller stability than H_t_SO_4_···w* (w13). Furthermore, the transition potential seems to depend on the ionic strength, once again highlighting the electrolyte effect on EDL properties. The authors clarified the nature of this transition and attributed it to the deprotonation reaction displayed in eq 16. At lower potentials, configurations involving bisulfates exhibit higher stability due to sulfate ion’s greater affinity for protons. Upon increasing the potential, the situation reverses. The electrostatic potential energy associated with sulfate configurations overtakes that of bisulfate configurations, considering both the proton affinity and electrostatic potential energies. This shift promotes the adsorption of sulfate ions. Additionally, coadsorbed water molecules form hydrogen bonds with adjacent water species and sulfate ions, leading to further stabilization of the adstructure at elevated potentials.
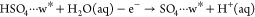
16

#### 4.3.2 Platinum

The interaction of water with the Pt(111) surface has been the emphasis of much research during the last few decades. Here, we emphasize several experimental^328−331^ and theoretical^332^ works in this area to demonstrate the high complexity of this seemingly simple system. It has been suggested that the first wetting layer forms a classical bilayer √3 × √3–*R*30° superlattice on hexagonal d-band metals.^333−335^ However, high-resolution helium atom scattering during the growth of 2D ice layers revealed rotational ordering that leads to well-ordered, epitaxially rotated islands with symmetries of √37 × √37–*R*25.3° or √39 × √39–*R*16.1°.^335,336^ Using low-energy electron diffraction, similar observations were made for coverage-dependent water islands.^337,338^ While a previous STM investigation^33^ could not observe such periodicities, Standop et al.^34^ and Nie et al.^35^ both reported the observation of √37 × √37–*R*25.3° and √39 × √39–*R*16.1° using STM. In what follows we want to highlight the results from Nie et al. and especially refer to ref (335), providing an excellent and unique discussion about the results.

**Figure 12:**
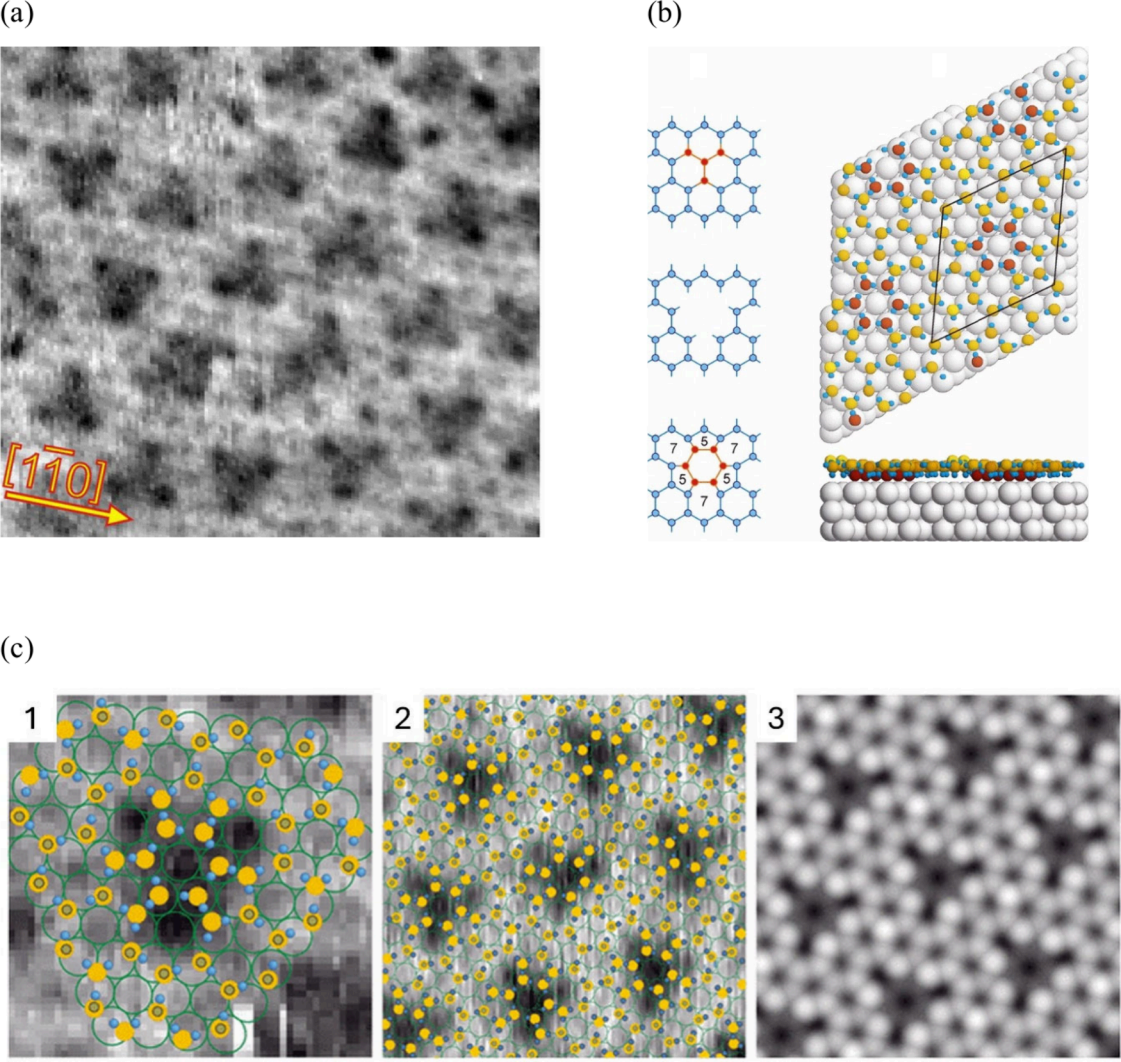
(a) 8 nm × 8 nm STM image of a triangular depression observed at submonolayer water on Pt(111) at 140 K using tip voltage *V*_tip_ = 0.5 V and tunneling current *I*_t_ = 1 pA. (b) DFT simulations of water molecule arrangement in a √37 water phase on Pt(111). Yellow and brown oxygen atoms visualize relative height variations induced from the 575757 di-interestitial defects of the hexagonal lattice. The respective defect and its formation are visualized in an additional schematic. (c) Combination of STM image and DFT results for single triangle and ordered √37 water phases on Pt(111). The third subimage depicts a simulated STM image generated from DFT-calculated charge densities. Reprinted with permission from ref (35). Copyright 2010 American Physical Society.

Nie et al.^35^ employed STM to explore 2D islands of wetting layer at low coverage levels, as displayed in Figure 12a. Evidently, the arrangement appears to be somewhat disordered, as indicated by the dark triangular spots that are positioned slightly lower than the surrounding, lighter areas of water molecules (∼0.3 Å). Surrounding these depressed spots, water molecules arrange hexagonally but with a 30° tilted orientation than the conventional √3 bilayer model typically expects. The study from Nie et al. attributed the nature of these triangular depressions to “575757” di-interstitial defects within the hexagonal water molecule lattice. Here, the dark vertices of the triangle correspond to heptagon centers. Figure 12b shows the results obtained from DFT indicating how these di-interstitial defects can be incorporated into the relaxed √37-unit cell. The results show that the wetting layer consists of a mixture of five-, six-, and seven-membered water rings. Both experimental and theoretical work turned out to be compatible with each other and were further validated by simulating STM images based on DFT electron density (see Figure 12c). The binding energies of the di-interstitial arrangement suggest larger stability than any other hexagonal H_2_O network or conventional √3 H-down bilayer. Similar investigations were conducted for the higher coverage √39 phase.

**Figure 13:**
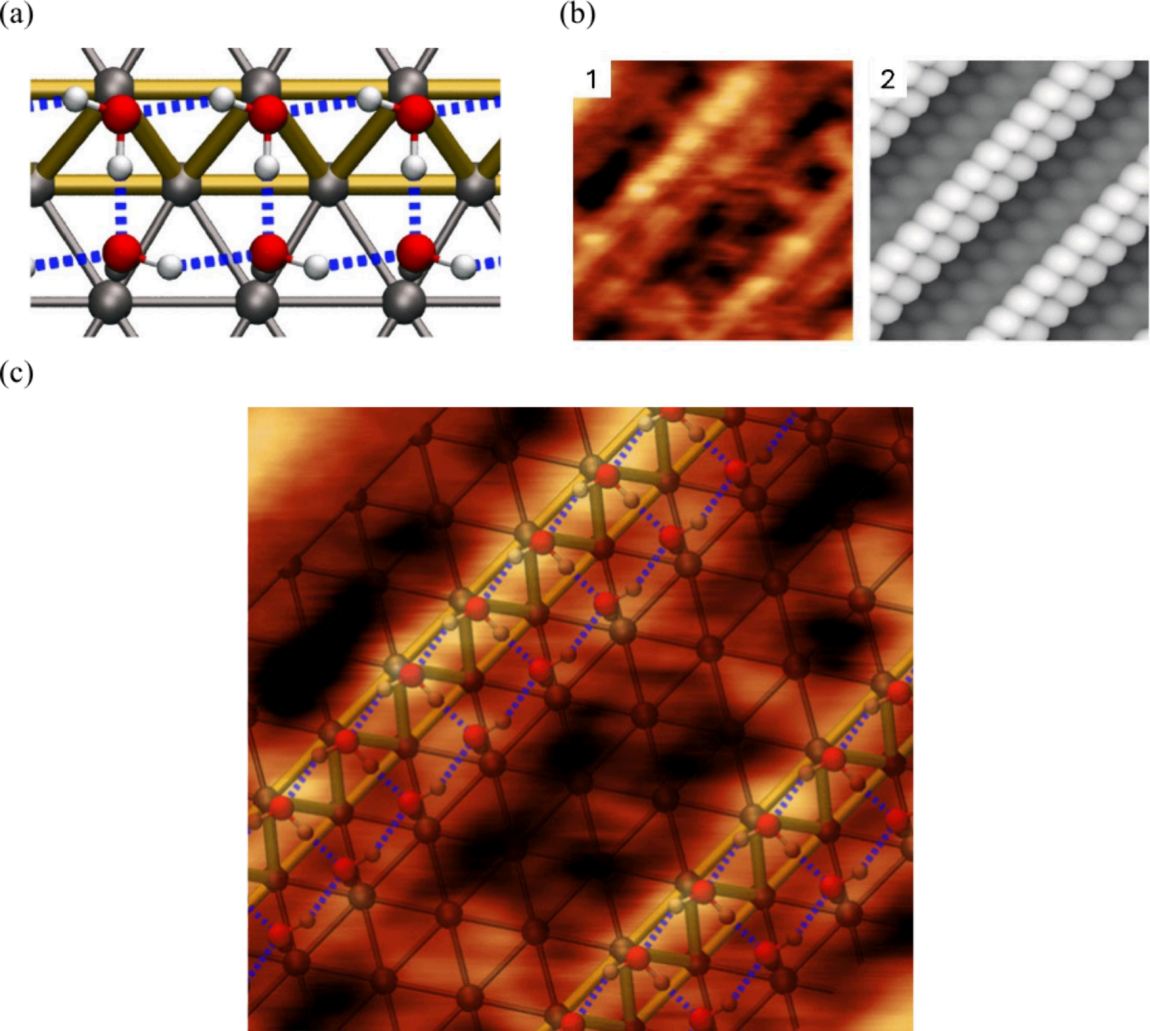
(a) Double-stranded, tetragonal 4–4–4 adsorption structure toward the lower terrace for increased coverage obtained from DFT. Gray, red, and white shapes correspond to platinum, oxygen, and hydrogen atoms. (b) Subfigure 1 corresponds to a 4.5 nm × 4.5 nm STM image of a D_2_O-covered step edge on Pt(553) visualized via STM using a voltage of −1 V and *I* = −9 pA at 25 K. Subfigure 2 displays the results from STM simulations of adsorbed water on Pt(553) (*V* = −0.9). (c) Combination of both STM and DFT results with the adsorption structure overlaying on the obtained STM image (4.5 nm × 4.5 nm). Reprinted with permission from ref (290). Copyright 2016 American Physical Society.

Furthermore, Kolb et al.^290^ studied the adsorption structures of water at the Pt(111) step with adjacent (111) terraces using both STM measurements and DFT calculations. The authors also highlighted other experimental^339^ and theoretical^297,340−347^ research conducted to investigate step edges for Pt single crystals. In their study, Kolb et al. identified water structures along the edges of Pt(553) steps, revealing patterns described as double-stranded, tetragonal 4–4–4 arrangements extending toward the lower terraces (Figure 13a). These unique structures, reported for the first time, are believed to result from a significant templating effect of the Pt(111) type step edge. Figure 13b displays STM images obtained of D_2_O-covered step edges on Pt(553) alongside calculated STM images. In these simulations, the O atoms of H_2_O are depicted as bright shapes. In the experimental images, the bright lines indicate step edges. Notably, the experimental images suggest that water molecules form parallel strands either above or below the step edge, an observation confirming templating. Both experimental and theoretical STM align well with each other, and thus, the resulting configuration is shown in Figure 13c.

**Figure 14:**
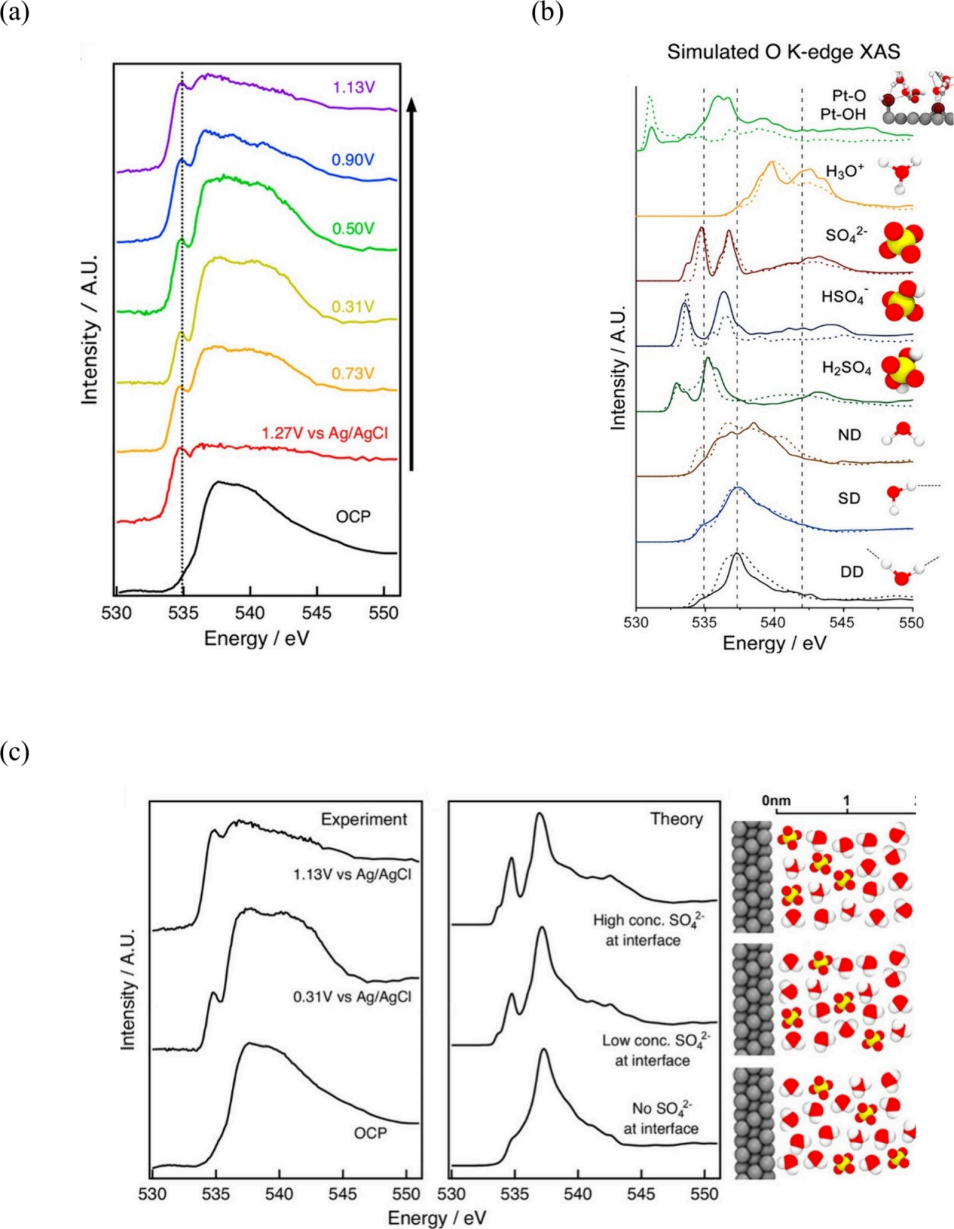
(a) Operando XAS spectra obtained for Pt in H_2_SO_4_ for different potentials, including information about the interfacial water structure. (b) The results of first-principles calculations of O K-edge XAS spectra, including different O-containing species at the Pt/H_2_SO_4_ interface. (c) The dependence of the composite simulated XAS spectra with varying SO_4_^2–^ concentrations at the Pt/H_2_SO_4_ interface and the respective molecular structure of the EDL. Reprinted from ref (178). Copyright 2018 American Chemical Society.

As discussed in our review, electrolytes play an important role in defining EDL structures and properties. While the previously discussed STM-based studies employed either H_2_O or D_2_O, we also provide some discussion for the Pt–H_2_SO_4_ (0.05 M) interface. The Pt–H_2_SO_4_ system has been thoroughly explored at voltages below 0.8 V vs RHE. However, debate remains over the molecular arrangement and chemical structure at more anodic potentials. To clarify the ambiguity, Wu et al.,^178^ recorded O K-edge EY-XAS spectra at different potentials, including the open-circuit potential (OCP) and more anodic potentials (Figure 14a). The pre-edge of the O K-edge EY-XAS spectra become more pronounced at larger anodic potentials, along with an extension of the plateau at (>540 eV). For deeper insights, the study also utilized several theoretical approaches, including first-principles molecular dynamics, multiscale simulations, and continuum thermodynamics models. We highlight the oxygen K-edge XAS spectra of oxygen-containing species and the comparison between experimental and theoretical O K-edge XAS spectra at various potentials, as depicted in Figure 14, parts b and c, respectively. The comparison of experimental and theoretical results suggests the adsorption of sulfate species at higher anodic potentials as indicated by a marked pre-edge feature at these potentials. However, bisulfates or sulfuric acid molecules do not appear to contribute to this spectral feature. Interestingly, water molecules do not contribute to the pre-edge peak at these anodic potentials. As discussed in previous studies (section 4.3.1), this behavior is likely due to reorientation, which suppresses the pre-edge. Most astonishingly, the study demonstrated the absence of Pt–OH formation and desorption of sulfate anions due to −OH adsorption,^348,349^ even at high anodic potentials. These findings are in contrast to other results obtained, for instance, from rate-dependent CV. The authors attributed this to the prolonged voltage application that corresponds to roughly 20 min at each step. This again highlights the high sensitivity of solid–liquid interfaces to the measurement details.

In summary, in this section, we delved into discussing several representative studies that combine experiments and simulations. We underscore the insights gained from STM investigations that have explored the wetting layer of ice on single crystals, facilitating the observation of superstructure periodicities at low temperatures. Nevertheless, the obtained results raise the question of whether similar periodicities of the superstructures manifest under ambient conditions and how relevant they are toward predicting the electrocatalytic activity, given the structural dependency of interfacial water on the electrode bias.

Furthermore, it was shown that utilizing XAS combined with AIMD simulations enables the analysis of the molecular structure of interfacial water. However, it remains unclear how key parameters, such as the types of hydrogen bonds at the surface and the orientation of water molecules, correlate with electrocatalytic activity. Similarly, using EC-Raman with AIMD calculations revealed the orientation of water molecules as a function of the electrode potential. From our perspective, two approaches seem promising for enhancing our understanding of the interconnection between interfacial water and electrocatalytic activity. First, it is essential to explore whether there is a correlation between the population of different molecule orientations (nondonor (ND), double donor (DD), single donor (SD) in parallel (SD^∥^) and perpendicular (SD^⊥^)) and the electrocatalytic activity. Second, by quantifying the molecular structure of interfacial water through parameters such as the number of hydrogen bond donors (*N*_donor_), a novel parameter can be established that facilitates the direct investigation of its correlation with electrocatalytic activity.

Quantifying interfacial water and examining its relationship with electrocatalytic activity would align with more macroscopic approaches to study the EDL conducted by, for instance, CV, EIS, CO displacement, and LICT. These studies aim to determine global parameters such as the pzc, *C*_dl_, and PME, which offer a more macroscopic view of the EDL. The subsequent sections will focus on such studies and aim to highlight existing interconnections between EDL parameters and electrocatalytic activity.

## 5 The Role of Electrode Composition

The chemical compositions of electrodes can greatly influence the catalytic efficiency of electrochemical reactions. Depending on the reaction, catalytic activity strongly depends on the material class, for instance, noble^350−353^ and non-noble^352,354−359^ metals, metal oxides,^352,360−366^ and transition (di)chalcogenide metals.^352,367−369^ Furthermore, the concept of alloying catalyst materials,^352,370−373^ or deposition of monolayers/islands, is well-known;^374−376^ however, there is an increasing demand for more complex material compositions, such as ternary systems^352,377^ or high entropy alloys,^378−390^ as they can provide further opportunities for tuning catalytic properties.

Despite continuous efforts aimed at developing compositionally rich catalytic systems, the fundamental understanding of EDL properties remains largely limited to single-component model systems and, therefore, cannot fully explain the origin of many interesting observations for complex electrode materials. Unfortunately, only a limited number of investigations have addressed fundamental properties of complex electrodes, such as high entropy alloys, as alluded to below.

To improve the fundamental knowledge of the EDL, it is mandatory to explore and compare EDL properties for different chemical compositions of the surface and subsurface of the electrode under similar experimental conditions, i.e., identical electrolyte chemistry and electrode composition and structure. The characteristics of the EDL can be quantified by several parameters, such as the *C*_dl_, pzc, and PME, which intend to describe the structure of the EDL macroscopically. The differences in those parameters arising from distinct electrode compositions seem to depend on the electronic structure and adsorption properties of the materials, leading to changes in the charge separation properties at the electrode–electrolyte interface.^391,392^ Consequently, the acquired fundamental understanding will help in developing a theory that reveals the relationships between the EDL structure and electrochemical activity and, therefore, clarify whether EDL parameters can serve as suitable descriptors of electrocatalytic activity.

### 5.1 Noble and Non-noble Metals, Metal Oxides, Transition (Di)chalcogenide Metals, and Carbon-Based Materials

The EDL properties of gold and platinum electrodes are much better understood than those of other electrode materials owing to their great electrochemical stabilities and well-defined double-layer regions. However, significant differences in the EDL properties of these two electrode materials exist attributed to notable differences in their electronic structures and adsorption properties. For instance, Xue et al.^393^ and Garlyyev et al.^198^ investigated the differential capacitances of Pt(111) and Au(111) in 0.05 M MeClO_4_ (Li^+^, Na^+^, K^+^, Rb^+^, Cs^+^). To determine the pzc and the corresponding *C*_dl_, they conducted EIS measurements with a physical EEC. Their model exhibits higher complexity compared to the classical R-CPE model since additional adsorption processes occurring in these specific aqueous electrolytes must be considered. In their work, *R*_a_ and *C*_a_ correspond to the adsorption resistance and capacitance, respectively, arising from OH* and H* adsorption.^198^ The results from Xue et al. and Garlyyev et al. show that, for instance, in 0.05 M LiClO_4_, the *C*_dl_ of Pt(111) in the vicinity of the pzc corresponds to ∼25 μF cm^–2^ and thus exhibits a significantly larger value compared to the *C*_dl_ of Au(111), which is ∼14 μF cm^–2^.

**Figure 15:**
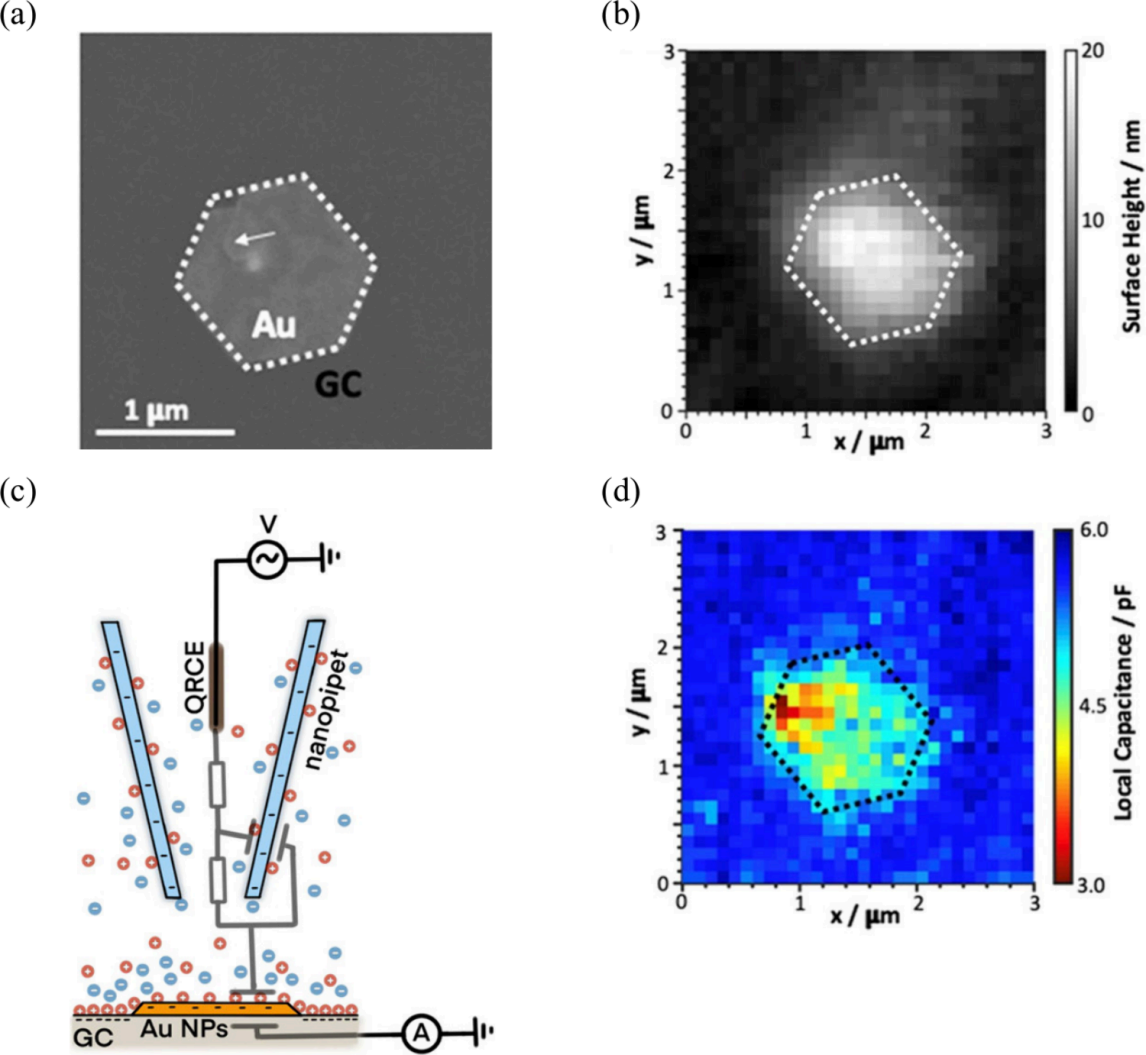
(a) Secondary electron SEM image of the Au nanoplate drop-casted on glassy carbon (GC), at which a dislocation within the Au nanoplate is highlighted by the inserted white arrow. (b) Topography obtained from the AC-SICM experiments. (c) Schematic of the AC-SICM setup, consisting of nanopipet, quasi-reference counter electrode (QRCE), and sample. Furthermore, the equivalent circuit is highlighted with the respective elements consisting of the bulk resistance *R*_b_, sample capacitance *C*_s_, and tip resistance and capacitance *R*_t_ and *C*_t_. (d) Local capacitance distributions obtained from the AC-SICM experiments (320 Hz, 50 mV peak-to-peak (ptp) amplitude, 50 mM KCl). Reprinted from ref (75). CC-BY.

Another well-studied material class in electrochemistry is carbon-based materials, as they are widely used as supporting materials for nanostructured catalysts^350^ or even as catalysts themselves.^394,395^ To reliably compare the influence of the electronic structure and adsorption properties of carbon with other material compositions, glassy carbon (GC) and highly oriented pyrolytic graphite (HOPG) were primarily explored. Shkirskiy et al.^75^ recently employed an AC-SICM approach to explore the capacitance difference from Au nanoplates drop-casted on a GC electrode with the GC itself, using a 50 mM KCl filling solution in a 100 nm radius nanopipet. A two-electrode setup was utilized for their experiments, which consisted of a quasi-reference counter electrode (QRCE) within the nanopipet and the sample as a working electrode. The sample was maintained at 0 V DC bias relative to the QRCE throughout the experiments, ensuring no significant Faradaic reaction contribution. The data were recorded with an effective sample–tip distance of 12 nm, a single frequency of 320 Hz, and a peak-to-peak amplitude of 50 mV, which were selected to adequately resolve interfacial capacitance without compromising spatial resolution. Figure 15a displays a secondary electron SEM image visualizing the Au nanoplates on GC. The topographical data obtained from the AC-SICM (Figure 15b) revealed thicknesses of the Au nanoplates ranging from 10 to 20 nm. The phase angle data from AC-SICM were modeled with an EEC depicted in Figure 15c using fixed values of *R*_b_ = 200 Ω, *C*_t_ = 5.5 pF, and *R*_t_ = 110 MΩ. Determining these EEC elements’ values from prior impedance measurements facilitates the estimation of local capacitances directly from the respective phase angle values. The resulting map of local capacitances is displayed in Figure 15d and reveals a distinct difference between the Au nanoplates (∼4 pF) and the GC substrate (∼6 pF). Further analysis yielded average local capacitance values of 6.8 pF for the GC and 4.3 pF for the Au nanoplates, which agreed reasonably well with the macroscopically determined capacitance values for GC of 40 μF cm^–2^ and for Au of 20 μF cm^–2^ considering a 15–22 μm^2^ probing area during AC-SICM experiments. Those probing areas agree with conducted numerical simulations that showed that the current density spreads over a wider area than the pipet radius with frequencies lower than 320 Hz, decreasing the spatial resolution.

**Figure 16:**
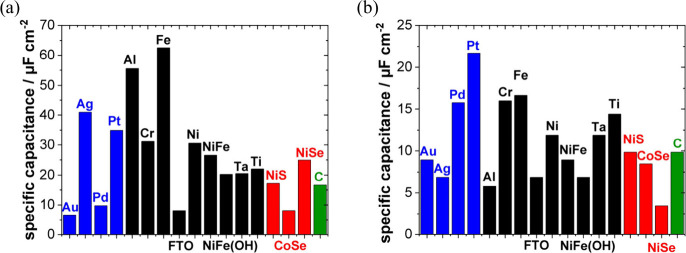
Double layer capacitances measured in (a) 0.15 M NaClO_4_ and (b) 0.15 M KPF_6_ and CH_3_CN with ±50 mV potential versus open circuit potential for four different material groups: noble and base metals, metal chalcogenide, and carbon. Adapted from ref (396). Copyright 2018 American Chemical Society.

We have highlighted some studies comparing Au and Pt metals with each other, as well as a study comparing Au with GC. However, the question arises as to how the determined EDL properties change as a function of the noble metal nature, as well as in comparison with base metals or metal chalcogenides. To address this question, Yoon et al.^396^ conducted experiments for four different material groups: noble and base metals, metal chalcogenide, and carbon. In this study, the results were obtained using the CV method via cycling in a potential range of ±50 mV around the open circuit potential in aqueous 0.15 M NaClO_4_ and in 0.15 M KPF_6_ and CH_3_CN at different scan rates ranging from 5 to 50 mV s^–1^ (Figure 16). Yoon et al. discovered a drastic difference between Au and Pt independently of a protic or aprotic electrolyte. In the case of the aqueous 0.15 M NaClO_4_, Pt exhibited a specific capacitance of 35 μF cm^–2^, while the capacitance for Au corresponds to ∼7 μF cm^–2^. Two additional noble metals, Ag and Pd, were characterized. While Ag exhibited the largest *C*_dl_ among all considered noble metals of 40 μF cm^–2^, Pd was characterized by a slightly higher value (∼10 μF cm^–2^) compared to Au. This significant difference between different noble metals might be attributed to a different degree of specific adsorption of OH_*x*_ species. At the same time, both Na^+^ and ClO_4_^–^ are known to be weakly coordinated at metal surfaces.^397^

Furthermore, the *C*_dl_ values obtained for noble metals were compared with those for base metals,^396^ for which the complexity of EDL measurements significantly increases due to the tendency of base metals to be oxidized. Therefore, it is essential to point out that, for Al, Cr, Fe, Ni, Ni_0.8_Fe_0.2_, Ta, and Ti, the surface was oxidized under experimental conditions, while the chemical composition of the bulk most likely remained in its fully reduced state. In the case of base metals, we also need to differentiate between oxide-passivated base metals and fully bulk conductive oxides like fluorine doped tin oxide (FTO) in which not only the surface but the entire bulk material is oxidized. Another chemical composition that arises for hydroxides is NiFe(OH). Generally, surface oxidation leads to enhanced adsorption of H^+^ and OH^–^ at the surface and, therefore, to larger *C*_dl_ values compared to noble metals. Yoon et al.^396^ especially pointed out the significant difference between FTO and surface oxidized Fe with an 8 times difference in the *C*_dl_ values. However, since the experiments were not conducted using single crystals, the question arises of whether the difference in *C*_dl_ values is purely due to the distinct chemical composition or due to the different crystallographic structures at the surface influencing the EDL properties. In addition, Yoon et al.^396^ investigated metal chalcogenides (NiS_*x*_, CoSe_*x*_, NiSe_*x*_)—specifically, the comparison of CoSe_*x*_ with NiSe_*x*_—which improves our understanding of how Ni and Co in Se-based metal chalcogenides affect EDL properties. The results revealed a significant increase in *C*_dl_ from ∼8 to ∼25 μF cm^–2^ by changing from CoSe_*x*_ to NiSe_*x*_, highlighting the different H^+^/OH^–^ adsorption properties and electronic structures. Similar experiments conducted in 0.15 M KPF_6_ and CH_3_CN demonstrated that specific capacitances are less scattered compared to aqueous electrolytes, and thus smaller deviations between different materials emerge. According to the hypothesis by Yoon et al., the H^+^/OH^–^ adsorption processes in protic electrolytes crucially depend on the chemical composition of the electrode, leading to more scattered *C*_dl_ values among the investigated materials.

While this study gives a comprehensive view of the influence of material composition on the *C*_dl_ within one experimental work, the question remains whether these *C*_dl_ values can be correlated with the electrocatalytic activity of the electrode. To address this question, activity measurements need to be performed with the discussed catalytic systems in similar electrolytes.

**Figure 17:**
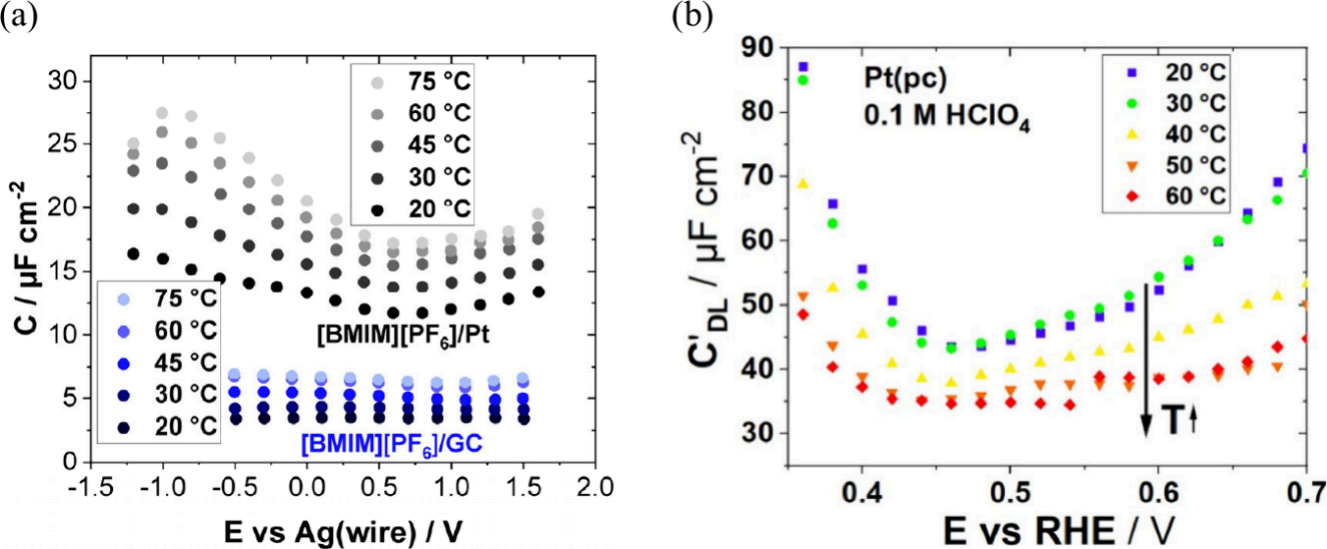
Differential capacitance curves in dependence of the potential and the temperature for (a) Pt and GC in [BMIM][PF_6_]^398^ and (b) Pt in 0.1 M HClO_4_.^154^ (a) Adapted with permission from ref (398). Copyright 2008 Elsevier. (b) Reprinted with permission from ref (154). Copyright 2021 Elsevier.

Nevertheless, if a correlation between *C*_dl_ parameters and electrocatalytic activity exists, then the *C*_dl_ parameter should be a function of temperature. Temperature is a key parameter in electrochemistry since it directly influences the reaction kinetics of Faradaic processes. The temperature dependence of *C*_dl_ was reported by Silva et al.,^398^ who investigated the *C*_dl_ values of Pt and GC. Classical EIS was used for the experiments with a physical EEC consisting of resistance *R*_s_ related to the resistance arising from the electrolyte/ionic liquids and a CPE describing the *C*_dl_. The *C*_dl_ dependence on the potential in the [BMIM][PF_6_] electrolyte is shown in Figure 17a for Pt and GC in a temperature interval from 20 to 70 °C. These experiments show that, in the case of [BMIM][PF_6_], the capacitance values increase with increasing temperature in the entire range of applied potentials independently of the electrode composition (Pt/GC). Moreover, the shape of the capacitance curves does not change significantly. For the position of the minimal capacitance, no significant shift in the presented figures occurs; nevertheless, one should not forget that, with increasing temperature, the equilibrium potential of the reference electrode changes. An opposite tendency for *C*_dl_ with changing temperature was detected by Watzele et al.,^154^ who investigated *C*_dl_ for Pt(pc) in aqueous HClO_4_ using EIS with an R-CPE model, including adsorption resistance *R*_a_ and capacitance *C*_a_, to consider specific adsorption at the interface. As can be seen in Figure 17b, *C*_dl_ decreases with increasing solution temperature for Pt(pc) in aqueous HClO_4_. The authors ascribed this observation to the reduced dielectric constant ε_r_ at higher temperatures. The two studies underscore the necessity of using identical conditions, such as the same electrolytes and crystallographic orientations, when comparing different material compositions. Moreover, whether a similar temperature trend emerges for other noble metals or more complex material compositions such as oxides is uncertain.

### 5.2 Non-noble Metals and Dielectric Materials Explored by Localized Electrochemical Impedance Spectroscopy

So far, we have discussed the results obtained from traditional macroscopic approaches based on EIS and voltammetry to explore EDL properties. To investigate these properties at micrometer length scales, LEIS via SECM could provide valuable insights. During LEIS experiments, the perturbation voltage is applied to the microelectrode, and thus, the interfacial properties at the sample are indirectly probed. Below, we discuss two studies investigating the *C*_dl_ distribution across metal compounds partially coated with dielectric materials by means of LEIS.

**Figure 18:**
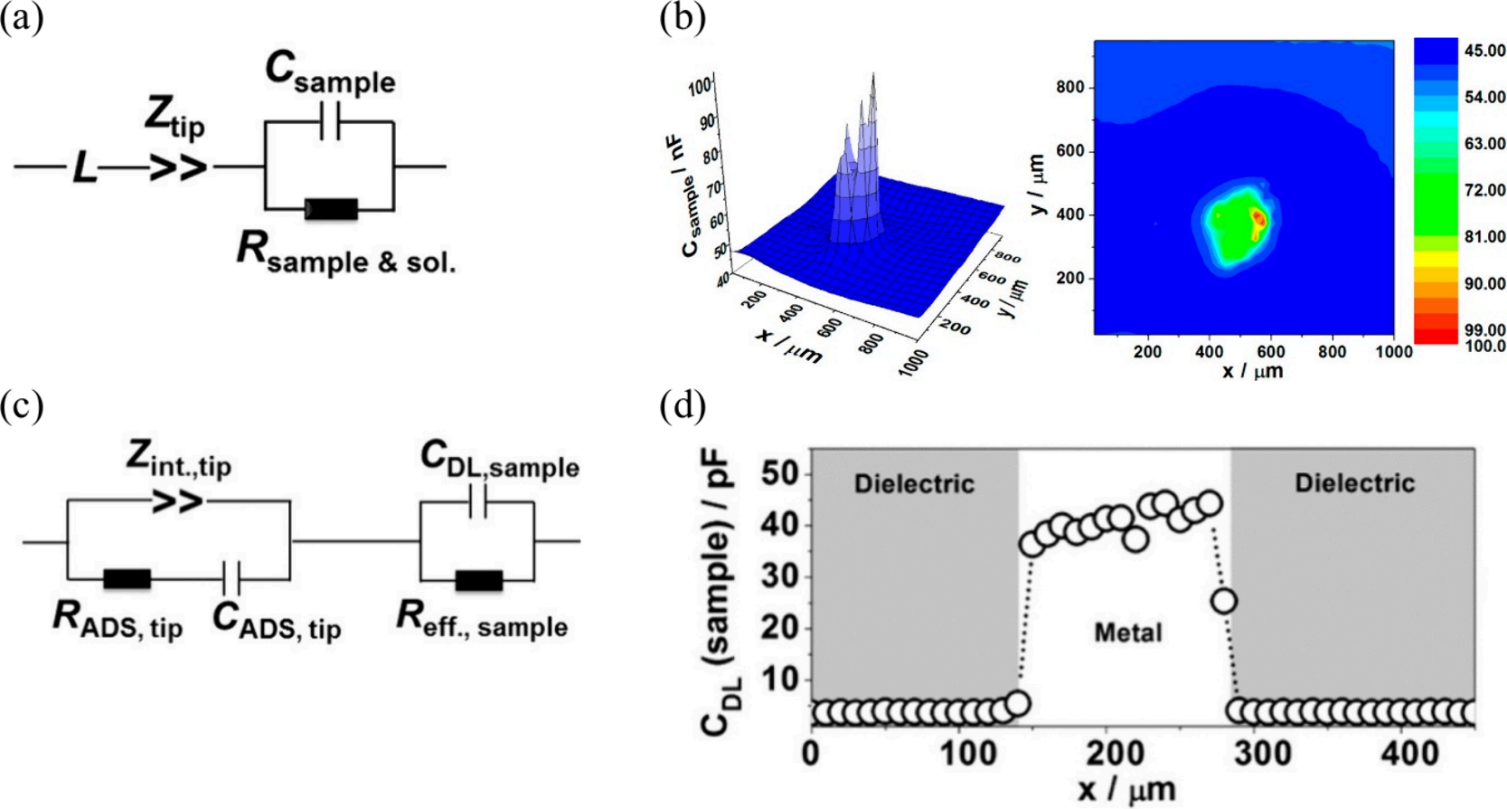
Scheme of the EEC consisting of an inductance *L* compensating nonideal potentiostatic behavior, CPE (Ztip) and *C*_Sample_ considering the *C*_dl_ at the microelectrode tip and sample, respectively, such as *R*_sample&sol._, which considers contributions of charge transfer and electrolyte resistance. (b) *C*_dl_ map of the low-carbon steel coated with tin and protected by an epoxy phenolic varnish obtained by LEIS.^73^ (c) Modified EEC compared to (a) without inductance, but therefore including adsorption resistance *R*_ads_ and capacitance *C*_ads_ at the tip considering specific adsorption processes. (d) *C*_dl_ deviation of the Al alloy (oxide) and poly(methyl methacrylate) (PMMA) along one line scan.^244^ (a, b) Reprinted from ref (73). Copyright 2013 American Chemical Society. (c, d) Reprinted/adapted with permission from ref (244). Copyright 2014 American Chemical Society.

Because of the four-electrode configuration employed in LEIS, more complex EECs than those traditionally used in conventional EIS are mandatory. Bandarenka et al.^73^ employed an EEC (Figure 18a) that included an inductor to compensate for potentiostatic deviations at high frequencies and a CPE element modeling the EDL at the microelectrode tip. A capacitor for the EDL and a resistance, which considers Faradaic processes and the electrolyte resistance, models the interface of the sample. Bandarenka et al. employed very low concentration 1 mM KClO_4_, with the anion ClO_4_^–^ usually not participating in Faradaic processes nor specifically adsorbing at the electrode. They explored low-carbon steel coated with tin and protected by an epoxy phenolic varnish (∼9–15 μm thick). After introducing a circular defect of ∼220 μm in diameter in the varnish to reveal the underlying metal, they observed a significant difference in the *C*_dl_ between the exposed metal (70–100 nF) and the dielectric area (45 nF), as shown in Figure 18b. In another study, Bandarenka et al.^244^ explored an aluminum alloy coated with poly(methyl methacrylate) (PMMA) as a dielectric material. A higher electrolyte concentration (10 mM) and chloride ions (Cl^–^) were used, requiring a modified EEC model (Figure 18c) to account for adsorption resistance and capacitance at the tip. They found distinct *C*_dl_ values for the dielectric material (3 pF) and the Al alloy (oxide) (∼40 pF). A considerably lower capacitance observed for the dielectric material could be attributed to its insulating characteristics. However, the sample–tip distance may influence the accuracy of the *C*_dl_ determination.

In conclusion, applying LEIS coupled with fitting to a physical EEC opens a new field of research in interfacial electrochemistry. However, it is crucial to mention that all the research conducted using LEIS was done without potential control of the sample. As a result, only limited insights can be obtained via LEIS measurements since the EDL structure inherently depends on the applied potential. Furthermore, we highlight that all LEIS measurements were carried out with low electrolyte concentrations (1–10 mM). To probe the sample’s interfacial properties using LEIS experiments, the electrolyte resistance needs to be marginally large, which is only possible in the case of low concentrations. This reflects a significant limitation on EDL research since the EDL structure and, thus, its properties significantly depend on the ionic strength of the electrolyte (as will be shown in section 7).

### 5.3 Monolayers/Islands on Bulk Single Crystals

Our discussion so far has primarily focused on relatively simple chemical compositions and compounds; however, more complex catalytic systems have been known for decades, and the comprehension of their EDL properties is necessary to understand the origin of their activity. One promising class of systems in electrocatalysis is core–shell nanoparticles or nanostructures decorated with islands or adatoms of different material compositions on the surface. To study such systems, monolayers (MLs), islands, or adatoms deposited on the surface of a bulk single crystal are typically synthesized. The lattice constants of the ML material deposited on top of single crystals vary depending on the number of MLs deposited and the single crystal’s chemical composition. The mismatch in the lattice constants leads to modifications of physical and chemical properties and, hence, to significant differences in double-layer properties.

**Figure 19:**
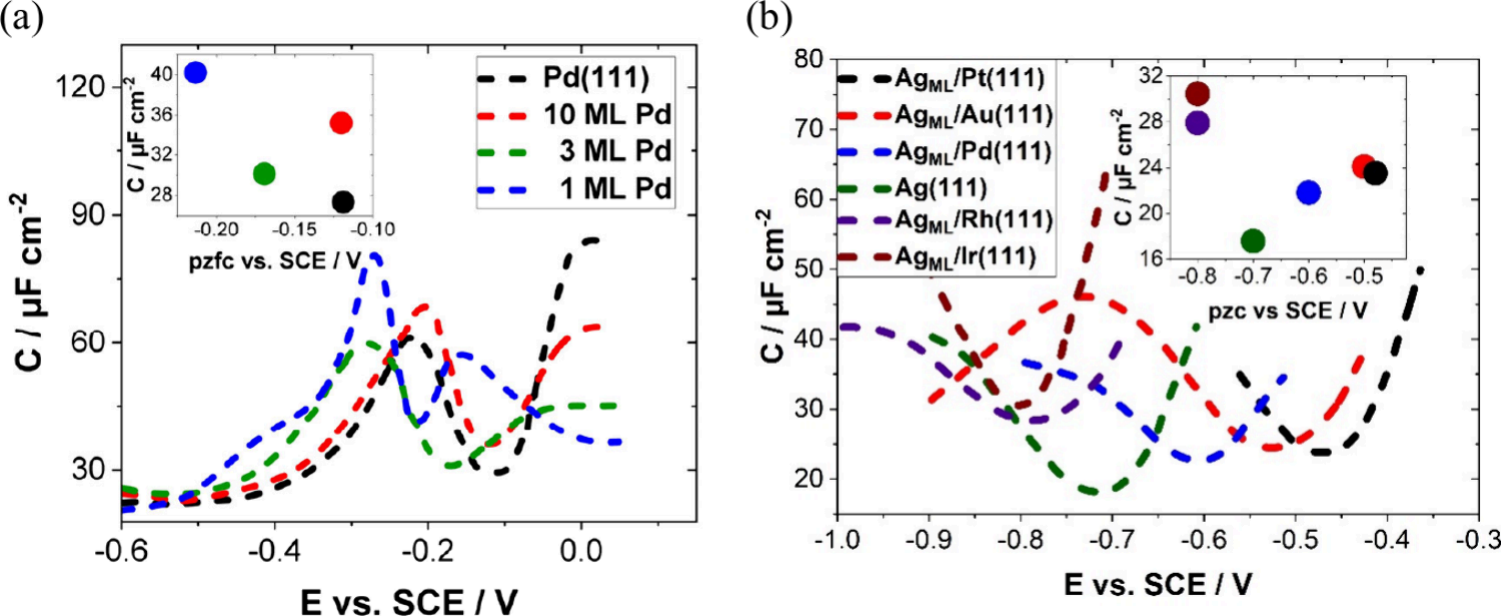
(a) Capacitance curves in dependence of the applied potential for different amounts of Pd MLs on Pt(111) measured in 5 mM NaF by using a scan rate of 10 mV s^–1^ and a frequency of 18 Hz.^399^ (b) Capacitance curves in dependence of the applied potential for Ag MLs on different fcc(111) single crystals, including Ir, Rh, Pd, Au, and Pt, as well as pure Ag(111) measured in 5 mM KClO_4_ by applying a scan rate of 10 mV s^–1^ and a frequency of 20 Hz.^400^ The insets in (a) and (b) display the estimated *C*_dl_ values at the corresponding pzc/pzfc, extracted from (a) and (b). (a) Adapted with permission from ref (399). Copyright 2005 Elsevier. (b) Adapted with permission from ref (400). Copyright 2007 Elsevier.

El-Aziz et al.^399^ explored a system comprised of Pd monolayers on Pt(111) via EIS and compared the observed *C*_dl_ properties to those of pristine Pt(111) using an 18 Hz and 10 mV peak-to-peak sinusoidal perturbation in 5 mM NaF. Depending on the number of Pd MLs, a shift in the pfzc appears, which diminishes upon increasing Pd layer thickness. Consequently, the position of the pzfc for 10 ML of Pd approaches the one of pure Pd(111), as shown in Figure 19a. Similar to a change in the pzfc, the respective capacitances at the pzfc seem to depend on the number of Pd MLs on Pt(111), which generally decreases with increasing Pd thickness. The *C*_dl_ values correspond to roughly 41, 31, 36, and 28 μF cm^–2^ for 1 ML, 3 ML, 10 ML, and Pd(111), respectively. One year after the work of El-Aziz et al., Soliman et al.^400^ went one step further in investigating the material effect on *C*_dl_ properties and investigated Ag pseudomorphic MLs on various single crystals in 5 mM KClO_4_ at 20 Hz with a scan rate of 10 mV s^–1^ and a 10 mV sinusoidal peak-to-peak perturbation. The single crystal’s structure corresponds to (111) and is comprised of different materials like Ir, Rh, Pd, Au, and Pt. Furthermore, the EDL properties of the deposited Ag MLs on those single crystals were compared with those of pure Ag(111). Depending on the material of the underlaying single crystal, a shift in the pzc of up to 0.3 V was observed, and the corresponding *C*_dl_ changed relative to pure Ag(111), as illustrated in Figure 19b. The authors point out the absence of a correlation between the lattice constant of the underlying single crystals and the respective pzc. Nevertheless, the d-band center shifts appear to follow a pattern similar to that of the pzc.

Next, Climent et al.^401^ examined the impact of electrode composition on the PME using the LITJ technique for a Pt(111) single crystal, which was modified by adsorbing bismuth adatoms. It is worth noting that 0.1 M HClO_4_ (pH 1) and 0.1 M KClO_4_ + 1 mM HClO_4_ (pH 3) electrolytes were used. At both pH levels, it was found that the PME varies with the bismuth coverage as follows: The PME decreases at high coverage, and the PME increases at low coverage. These observations were attributed to several factors, such as the mobility of bismuth adatoms and its influence on the entropy of the double-layer formation, the spillover of electron density, and the entropic contribution, among others.^401^ However, a subsequent comprehensive investigation of the influence of the electrode composition on the PME was not conducted until 2021, when Auer et al. explored a modified Cu(111) electrode surface adsorbing either 0.1 or 0.2 ML of Ni(OH)_2_ in alkaline media.^114^ Interestingly, upon Ni(OH)_2_ deposition, the PME exhibited a trend in the order Cu(111) < 0.2 ML of Ni(OH)_2_ < 0.1 ML of Ni(OH)_2_. Moreover, in the case of 0.2 ML of Ni(OH)_2_ deposited on Cu(111), two PMEs for the double-layer formation were identified. The second PME value was found to be smaller than the value for Cu(111). The chemical composition of the electrode material influences the solid/liquid interface, such as the interfacial water molecule reorganization, which the PME descriptor can characterize.^128,402,403^

### 5.4 High Entropy Alloys

The continuous search for catalysts with higher activities with reduced shares of noble metals has led to an increasing interest in ternary, quaternary, and so-called high entropy alloys (HEAs). However, the complexity of such catalytic systems further increases, which often impedes unambiguous elucidation of the origin of electrochemical activity. In the following, we discuss a pioneering study on determining EDL properties of HEAs despite significant reliability challenges. These difficulties stem from overlapping double-layer regions of the respective elements within the HEA and emerging phase transitions during potential cycling of the HEA, as evidenced by Pourbaix diagrams.^247^ However, this innovative approach based on the SECCM technique was shown to be capable of determining the pzc’s of HEAs. A brief introduction to the SECCM technique is provided in section 3. In 2023, Kim et al.^247^ employed SECCM to explore the correlation between the pzc, the work function, and the electrocatalytic HER activity of an HEA using a 10 mM HClO_4_ electrolyte. The methodology for the pzc determination involves monitoring the capacitive charging current associated with EDL formation as the SECCM nanopipet slowly establishes physical contact with the sample. By retracting the nanopipet after each measurement, slightly adjusting the sample potential, and then remeasuring the capacitive charging current upon recontact, the researchers could map out the pzc across a wide range of sample potentials. The absence of a capacitive charging current at the transient potential indicates the position of the pzc, with negative currents being observed at potentials that are more negative than the pzc and positive currents observed at more positive potentials.

**Figure 20:**
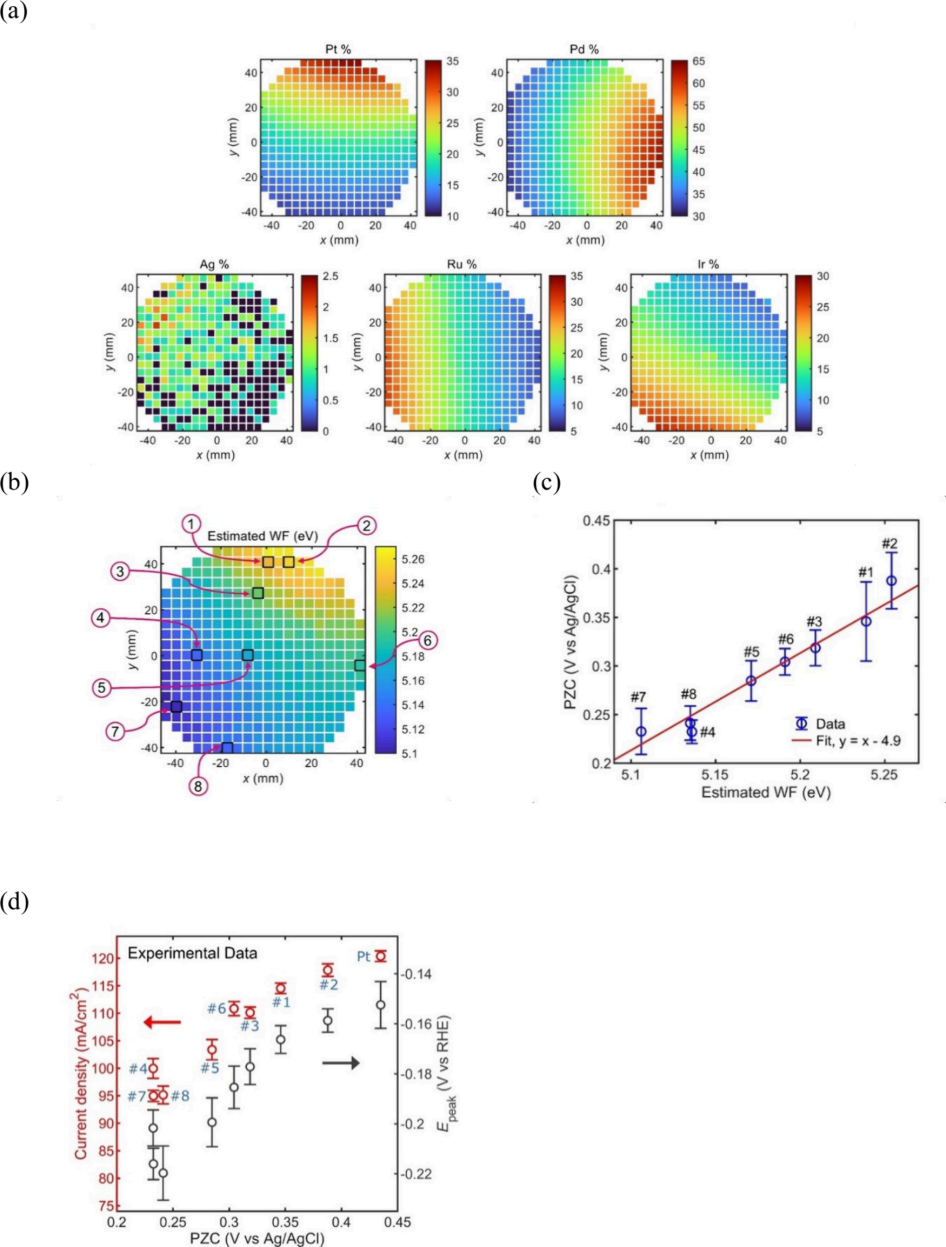
(a) Energy dispersive X-ray (EDX) results identifying the composition gradient and ranges of Pt, Pd, Ru, Ir, and Ag HEAs. (b) Estimated work functions of the respective HEAs shown in (a). (c) Correlation of the pzc determined via approaching SECCM method in correlation with the determined work function of eight different HEAs, marked in (b) via numbers. (d) Dependency of the determined pzc of the HEA shown in (c), and for Pt as reference, and the HER current density evaluated at −100 mV vs RHE and the peak potential *E*_peak_ during the conducted CV. Reprinted from ref (247). CC BY NC-ND 4.0.

Different Pt, Pd, Ru, Ir, and Ag HEAs were synthesized using cosputtering on a silicon wafer, and the respective elemental compositions of the distinct HEAs were detected via energy dispersive X-ray (EDX) analysis, as displayed in Figure 20a. From the determined elemental fractions, the composition-weighted average work functions were calculated for each synthesized HEA, as displayed in Figure 20b. Since the work function is a suitable descriptor for correlating the elemental composition with the pzc, the study focuses on eight HEAs with work functions (WFs) ranging from 5.11 to 5.25 eV, as shown in Figure 20c. The analysis revealed a linear correlation between the WF and the pzc, indicating that alloys with a higher WF exhibit a higher pzc, which is in agreement with previous theoretical literature.^101,108^ Furthermore, CV experiments in 10 mM HClO_4_ were conducted for those eight HEAs to evaluate their HER activities. Two parameters for HER activity comparison were chosen, corresponding to the current density at −100 mV vs RHE and the potential *E*_peak_ corresponding to the largest current density in the forward scan of the CVs. The results are displayed in Figure 20d and indicate larger HER current densities with larger pzc’s and a noteworthy positive shift in *E*_peak_ with increasing pzc, suggesting that materials with a more positive pzc tend to exhibit higher HER activity. The authors highlighted that these observations align with the results from Trasatti et al., who demonstrated that pure metal electrodes with higher WFs exhibited improved HER activity in acidic electrolytes, as measured by the exchange current density.^108^

We want to highlight once more the successful, pioneering attempts to study the pzc’s dependence on the WF from HEAs and their correlation with their electrocatalytic activities. Nevertheless, our knowledge fails to fundamentally explain the interconnection between the pzc and electrocatalytic activity, especially for complex systems. Furthermore, the WF corresponds to a global parameter; however, the catalytic efficiency of active sites crucially depends on the position of the atom in a specific crystallographic orientation. Therefore, conducting such experiments with single crystals would provide deeper and more fundamental insights into the correlation between EDL properties and electrocatalytic activity. In the case of single crystals, instead of the WF, the area-normalized generalized coordination number can be applied as a descriptor. We highlight that such considerations are mandatory to improve our fundamental understanding of the influence of EDL properties on electrocatalytic activity. Furthermore, the detected catalytic activities of the HEA for the HER show lower HER activities than classical Pt, and the question arises of whether the found correlation between the pzc, WF, and catalytic activity is as well-established for pronounced HER activities.

In this section, we have discussed some research efforts that examine the influence of the material composition on the EDL. A summary of selected EDL parameters discussed within this study is provided in Tables 1 and 2. It is established that the material composition significantly influences the EDL parameters such as the *C*_dl_, pzc, pzfc, and PME. These effects are attributed to variations in physical and chemical characteristics related to adsorption properties, water molecule interactions, and lattice constants. Some studies highlight that parameters pzc and PME both seem to correlate with electrocatalytic activity. However, such a correlation has not yet been established for the *C*_dl_ parameter. Nevertheless, investigating the connection between *C*_dl_ and electrocatalytic activity will be of major importance for the further advancement of our understanding of how the EDL affects electrocatalytic activity.

**Table 1:** Selected Representative Studies Exploring Variations in *C*_dl_ Values Based on Electrode (Sub)surface Compositions^a^

composition	*C*_dl_	potential	method	electrolyte	ref
GC	6 pF^d^	0 V DC	AC-SICM	50 mM KCl	(75)
Au	4 pF^d^	0 V DC	AC-SICM	50 mM KCl	(75)
GC	40 μF cm^–2^	0 V vs Ag/AgCl	EIS	50 mM KCl	(75)
Au	20 μF cm^–2^	0 V vs Ag/AgCl	EIS	50 mM KCl	(75)
Pt(111)	25 μF cm^–2^	pzc	EIS	0.05 M LiClO_4_	(198, 393)
Au(111)	14 μF cm^–2^	pzc	EIS	0.05 M LiClO_4_	(198, 393)
Au	7 μF cm^–2^	±50 mV OCP	CV	0.15 M NaClO_4_	(396)
Ag	41 μF cm^–2^	±50 mV OCP	CV	0.15 M NaClO_4_	(396)
Pd	10 μF cm^–2^	±50 mV OCP	CV	0.15 M NaClO_4_	(396)
Pt	35 μF cm^–2^	±50 mV OCP	CV	0.15 M NaClO_4_	(396)
Al^b^	56 μF cm^–2^	±50 mV OCP	CV	0.15 M NaClO_4_	(396)
Cr^b^	31 μF cm^–2^	±50 mV OCP	CV	0.15 M NaClO_4_	(396)
Fe^b^	62 μF cm^–2^	±50 mV OCP	CV	0.15 M NaClO_4_	(396)
FTO^c^	8 μF cm^–2^	±50 mV OCP	CV	0.15 M NaClO_4_	(396)
Ni^b^	31 μF cm^–2^	±50 mV OCP	CV	0.15 M NaClO_4_	(396)
NiFe^b^	27 μF cm^–2^	±50 mV OCP	CV	0.15 M NaClO_4_	(396)
NiFe(OH)	20 μF cm^–2^	±50 mV OCP	CV	0.15 M NaClO_4_	(396)
Ta^b^	21 μF cm^–2^	±50 mV OCP	CV	0.15 M NaClO_4_	(396)
Ti^b^	22 μF cm^–2^	±50 mV OCP	CV	0.15 M NaClO_4_	(396)
NiS	17 μF cm^–2^	±50 mV OCP	CV	0.15 M NaClO_4_	(396)
CoSe	8 μF cm^–2^	±50 mV OCP	CV	0.15 M NaClO_4_	(396)
NiSe	25 μF cm^–2^	±50 mV OCP	CV	0.15 M NaClO_4_	(396)
C	17 μF cm^–2^	±50 mV OCP	CV	0.15 M NaClO_4_	(396)
Au	9 μF cm^–2^	±50 mV OCP	CV	0.15 M KPF_6_, CH_3_CN	(396)
Ag	7 μF cm^–2^	–600 mV vs Ag/AgCl	CV	0.15 M KPF_6_, CH_3_CN	(396)
Pd	16 μF cm^–2^	±50 mV OCP	CV	0.15 M KPF_6_, CH_3_CN	(396)
Pt	22 μF cm^–2^	±50 mV OCP	CV	0.15 M KPF_6_, CH_3_CN	(396)
Al^b^	6 μF cm^–2^	±50 mV OCP	CV	0.15 M KPF_6_, CH_3_CN	(396)
Cr^b^	16 μF cm^–2^	±50 mV OCP	CV	0.15 M KPF_6_, CH_3_CN	(396)
Fe^b^	17 μF cm^–2^	±50 mV OCP	CV	0.15 M KPF_6_, CH_3_CN	(396)
FTO^c^	7 μF cm^–2^	±50 mV OCP	CV	0.15 M KPF_6_, CH_3_CN	(396)
Ni^b^	12 μF cm^–2^	±50 mV OCP	CV	0.15 M KPF_6_, CH_3_CN	(396)
NiFe^b^	9 μF cm^–2^	±50 mV OCP	CV	0.15 M KPF_6_, CH_3_CN	(396)
NiFe(OH)	7 μF cm^–2^	±50 mV OCP	CV	0.15 M KPF_6_, CH_3_CN	(396)
Ta^b^	12 μF cm^–2^	±50 mV OCP	CV	0.15 M KPF_6_, CH_3_CN	(396)
Ti^b^	15 μF cm^–2^	±50 mV OCP	CV	0.15 M KPF_6_, CH_3_CN	(396)
NiS	10 μF cm^–2^	±50 mV OCP	CV	0.15 M KPF_6_, CH_3_CN	(396)
CoSe	8 μF cm^–2^	±50 mV OCP	CV	0.15 M KPF_6_, CH_3_CN	(396)
NiSe	3 μF cm^–2^	±50 mV OCP	CV	0.15 M KPF_6_, CH_3_CN	(396)
C	10 μF cm^–2^	±50 mV OCP	CV	0.15 M KPF_6_, CH_3_CN	(396)
low-carbon steel coated with tin	70–100 nF^d^	OCP	LEIS	1 mM KClO_4_	(73)
epoxy phenolic varnish	45 nF^d^	OCP	LEIS	1 mM KClO_4_	(73)
Al alloy (oxide)^b^	40 pF^d^/0.1 μF cm^–2^	OCP	LEIS	10 mM KCl	(244)
PMMA	3 pF	OCP	LEIS	10 mM KCl	(244)
Pd(111)	27 μF cm^–2^	pzfc	dEIS	5 mM NaF	(399)
Pd_10 ML_/Pt(111)	35 μF cm^–2^	pzfc	dEIS	5 mM NaF	(399)
Pd_3 ML_/Pt(111)	30 μF cm^–2^	pzfc	dEIS	5 mM NaF	(399)
Pd_ML_/Pt(111)	40 μF cm^–2^	pzfc	dEIS	5 mM NaF	(399)
Ag_ML_/Ir(111)	30 μF cm^–2^	pzc	dEIS	5 mM KClO_4_	(400)
Ag_ML_/Rh(111)	28 μF cm^–2^	pzc	dEIS	5 mM KClO_4_	(400)
Ag_ML_/Pd(111)	22 μF cm^–2^	pzc	dEIS	5 mM KClO_4_	(400)
Ag_ML_/Au(111)	24 μF cm^–2^	pzc	dEIS	5 mM KClO_4_	(400)
Ag_ML_/Pt(111)	24 μF cm^–2^	pzc	dEIS	5 mM KClO_4_	(400)
Ag(111)	18 μF cm^–2^	pzc	dEIS	5 mM KClO_4_	(400)
Pt_31.2_Pd_43.3_Ir_9.8_Ru_14.2_Ag_1.4_	(0.35 ± 0.04) V vs Ag/AgCl	pzc	SECCM	10 mM HClO_4_	(247)
Pt_32.3_Pd_45.2_Ir_9_Ru_12.5_Ag_9.7_	(0.39 ± 0.03) V vs Ag/AgCl	pzc	SECCM	10 mM HClO_4_	(247)
Pt_26.5_Pd_44.6_Ir_11.7_Ru_16.1_Ag_1.1_	(0.32 ± 0.02) V vs Ag/AgCl	pzc	SECCM	10 mM HClO_4_	(247)
Pt_18_Pd_38.4_Ir_18_Ru_24.3_Ag_1.3_	(0.23 ± 0.01) V vs Ag/AgCl	pzc	SECCM	10 mM HClO_4_	(247)
Pt_19.6_Pd_45.6_Ir_16.1_Ru_17.6_Ag_1.1_	(0.28 ± 0.02) V vs Ag/AgCl	pzc	SECCM	10 mM HClO_4_	(247)
Pt_17.8_Pd_62.1_Ir_11.3_Ru_8.0_Ag_0.8_	(0.3 ± 0.01) V vs Ag/AgCl	pzc	SECCM	10 mM HClO_4_	(247)
Pt_13.4_Pd_33.6_Ir_23.8_Ru_27.9_Ag_1.4_	(0.23 ± 0.02) V vs Ag/AgCl	pzc	SECCM	10 mM HClO_4_	(247)
Pt_12.2_Pd_39.9_Ir_26.2_Ru_21_Ag_0.9_	(0.24 ± 0.02) V vs Ag/AgCl	pzc	SECCM	10 mM HClO_4_	(247)

a The table columns list the material (sub)surface compositions, corresponding *C*_dl_ values, potentials at which *C*_dl_ values were determined, methods used, electrolytes employed, and references cited. It should be noted that several *C*_dl_ values are estimated from the original published data. This table serves as a comprehensive overview of the cited literature.

b Oxide-passivated surface during experimental conditions with the bulk most likely in its fully reduced state.

c Fully bulk conductive oxides.

d No surface normalization due to the usage of scanning probe approaches.

**Table 2:** Selected Representative Studies Exploring Variations in pzc/pzfc Values Based on Electrode (Sub)surface Compositions^a^

composition	potential	parameter	method	electrolyte	ref
Ag_ML_/Ir(111)	–0.8 V vs SCE	pzc	dEIS	5 mM KClO_4_	(400)
Ag_ML_/Rh(111)	–0.8 V vs SCE	pzc	dEIS	5 mM KClO_4_	(400)
Ag_ML_/Pd(111)	–0.6 V vs SCE	pzc	dEIS	5 mM KClO_4_	(400)
Ag_ML_/Au(111)	–0.5 V vs SCE	pzc	dEIS	5 mM KClO_4_	(400)
Ag_ML_/Pt(111)	–0.48 V vs SCE	pzc	dEIS	5 mM KClO_4_	(400)
Ag(111)	–0.7 V vs SCE	pzc	dEIS	5 mM KClO_4_	(400)
Pd(111)	–0.12 V vs SCE	pzfc	dEIS	5 mM NaF	(399)
Pd_10 ML_/Pt(111)	–0.12 V vs SCE	pzfc	dEIS	5 mM NaF	(399)
Pd_3 ML_/Pt(111)	–0.17 V vs SCE	pzfc	dEIS	5 mM NaF	(399)
Pd_ML_/Pt(111)	–0.21 V vs SCE	pzfc	dEIS	5 mM NaF	(399)

a The table columns list the material (sub)surface compositions, the potential at which the respective parameters were determined, which parameter was explored (pzc/pzfc), methods used, electrolytes employed, and references cited. It should be noted that several pzc/pzfc values are estimated from the original published data. This table serves as a comprehensive overview of the cited literature.

Regarding catalyst development, there has recently been a clear trend toward more compositionally complex systems, such as HEAs, due to their promising catalytic characteristics relative to single-component systems. However, our fundamental understanding of the relationship between the EDL and electrocatalytic properties for complex systems remains relatively poor. Here, we would like to highlight several crucial aspects that should be considered when conducting experiments on such systems. First, despite using the same electrolyte to study different material compositions, the structures of the materials featuring distinct compositions must be identical. Comparing different crystalline structures, nanoparticles, or other nanostructured materials will not yield accurate insights. Employing single crystals with consistent crystal orientations seems to be a more suitable approach for studying material composition. Second, utilizing a consistent electrolyte across various material compositions is necessary to avoid dissimilarities emerging from various ionic concentrations.

### 5.5 DFT Simulations

**Figure 21:**
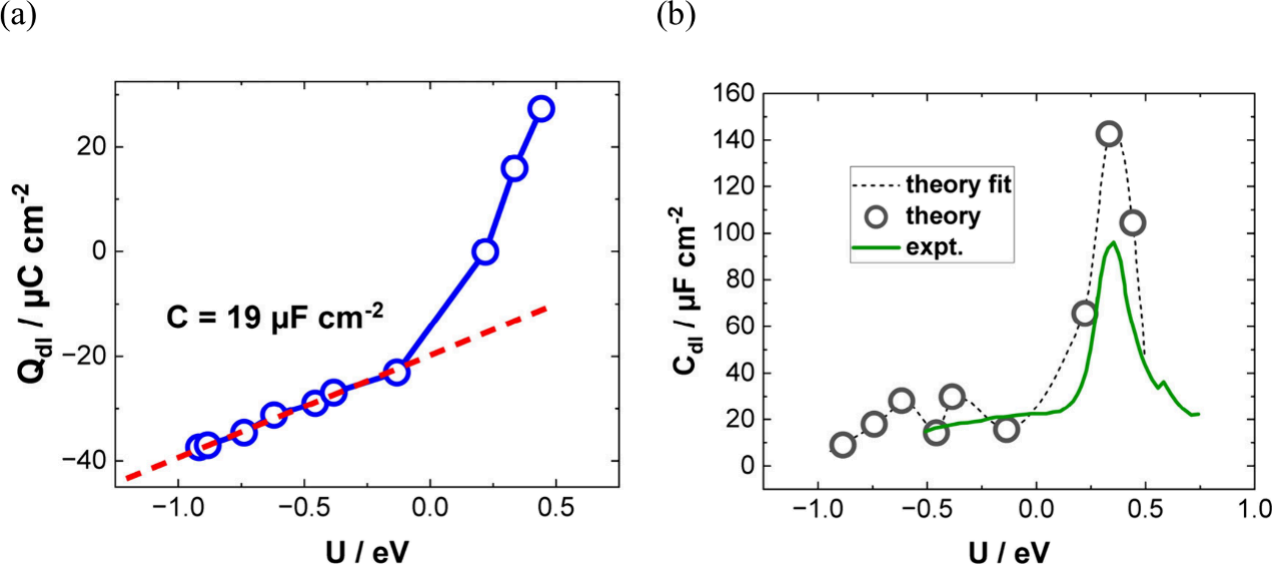
(a) *Q*_dl_ for Pt electrodes as a function of the applied electrode potential *U*. The red line is a linear fit used to derive the *C*_dl_ value. (b) Calculated *C*_dl_ as a function of applied potential *U* (gray points). The simulation points are fitted through a polynomial function (gray dashed line) and are compared to experimental data (green line). The energies are referred to as SHE. Adapted from ref (306). Copyright 2018 American Chemical Society.

There have been a series of DFT simulations focusing on understanding how the chemical composition of the electrode affects EDL properties (see, e.g., refs (83, 87, and 404)). In this section, we would like to highlight just a few recent DFT studies that focused on evaluating several fundamental EDL properties considering the electrified nature of electrodes. For example, in a recent DFT investigation based on constant Fermi-level molecular dynamics, the basic properties of the Pt(111)/water interface (*C*_dl_ and pzc) were examined under variable bias potentials.^306^ Within the applied theoretical formulation, the total electronic charge in the system is controlled and evolves according to fictitious Newton-like equations of motion. An alignment scheme was employed to reference the electrode potential to the SHE scale by considering the hydrogen adsorbed at the Pt electrode as an intermediate step between the aqueous hydronium ion and the gas phase H_2_. Figure 21 shows the change of the EDL charge (*Q*_dl_) as a function of the electrode potential (*U*) that allows one to estimate the capacitance as *C*_dl_ = d*Q*_dl_/d*U*. The dependence of *C*_dl_ on *U* compares well with the curve derived from experimental data. The estimated *C*_dl_ of 19 μF cm^–2^ agrees with the commonly reported experimental value of ∼20 μF cm^–2^.^405−407^ The main peak of the *C*_dl_ curve in Figure 21 at *U* = 0.33 eV corresponds to a rapid change in *C*_dl_ and is associated with the atomic-scale structural reorganization of the EDL. Specifically, it is found that at potentials above the pzc (*U* > 0.22 eV), the water structure is dominated by O_down_ configurations, in which the average dipole moment points toward the bulk water. On the other hand, at potentials lower than the pzc (*U* < 0.22 eV), water dipoles become oriented toward the Pt surface.

**Figure 22:**
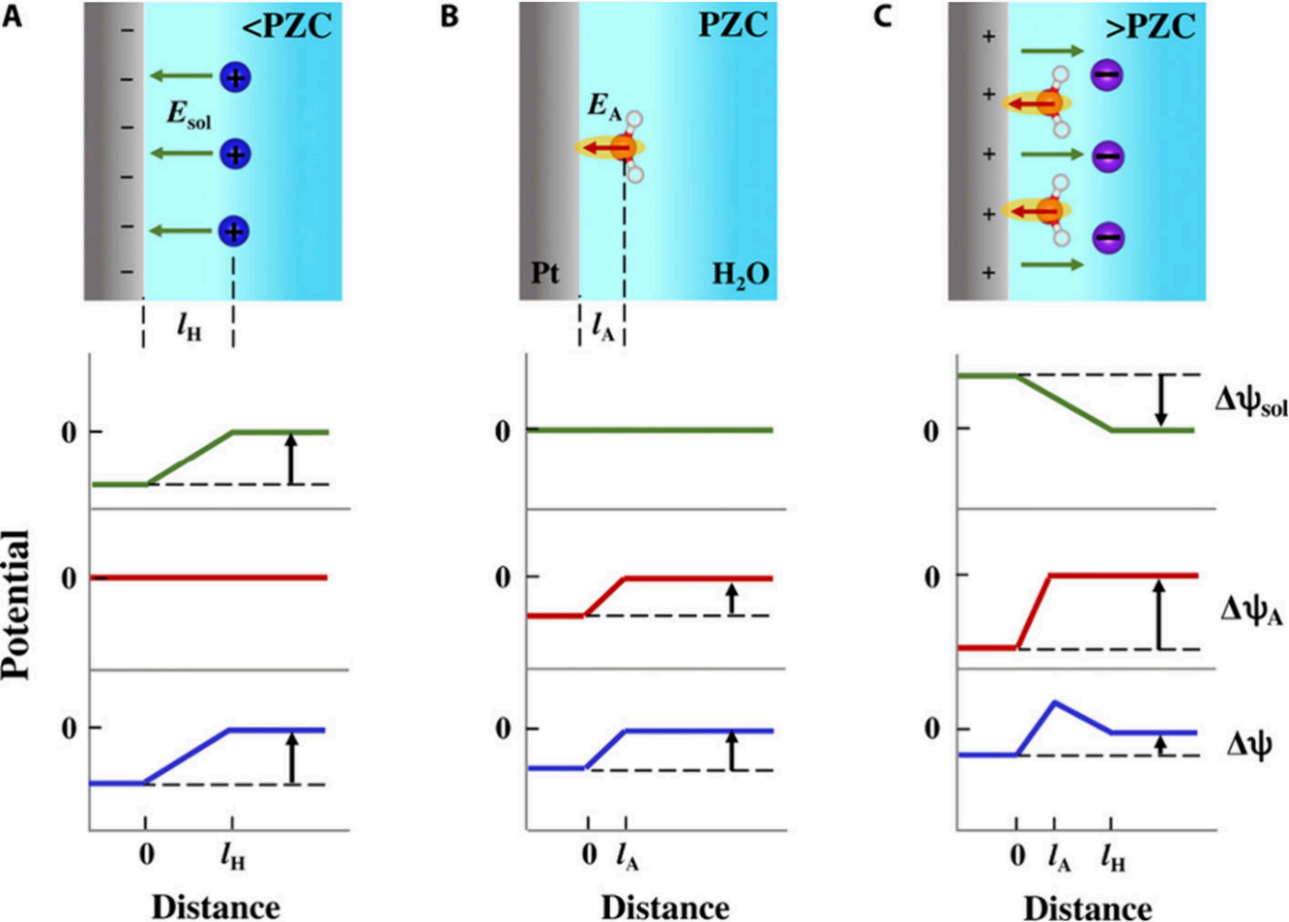
Proposed model of Helmholtz layer. Potential profiles at the Pt(111)water interface at different potentials: (A) <pzc without water chemisorption, (B) pzc, and (C) >pzc. The Pt electrode and water solution are the regions colored by gray and light blue, respectively. The balls in red, white, blue, and violet stand for oxygen atoms, hydrogen atoms, cations, and anions, respectively. The interface potential change Δψ (blue) consists of the usual potential change Δψ_sol_ induced by the surface charge (green) and the potential Δψ_A_ caused by water chemisorption. The potential in bulk solution is set to 0. *I*_A_ and *I*_H_ denote the distance separation of the dipole induced by chemisorbed water and the Helmholtz layer, respectively. Reprinted from ref (408). CC BY NC 4.0.

In another AIMD-based investigation,^408^ the puzzling origin of the bell-shaped differential Helmholtz capacitance at the electrified Pt(111)/water interface was examined. A detailed analysis of AIMD trajectories showed that the density and orientation of interfacial water molecules strongly depend on the applied potential. It was observed that the surface coverage of chemisorbed water goes up when the potential is shifted from negative to positive. This change in the coverage leads to a significantly modified interfacial dipole potential and causes a negative capacitive response. This is schematically illustrated in Figure 22.

In a recent investigation of the Au(111)/water interfaces, AIMD-based controlled-potential simulations were employed to examine the structural, hydrogen bonding, dynamic, and vibrational properties of water.^409^ It was determined that the relationship between the surface charge and the full trajectory-averaged electrode potential versus pzc is nearly linear. The derived differential capacitance of (14.3 ± 2.2) μF cm^–2^ was found to be within the range of experimentally measured values for dilute electrolytes.

## 6 The Role of the Electrode Surface Structure

The structure of the electrode (sub)surface is another critical parameter that influences phenomena at the electrode–electrolyte interface.^410^ Therefore, a deep understanding of how the atomic structure of electrode surfaces affects EDL properties is essential. For example, stepped metal surfaces often exhibit greater electrocatalytic activity than atomically flat surfaces.^411−414^ Consequently, single crystals offer a way to study structural effects on EDL properties due to their unique and well-defined surface structure. Although many research groups have been exploring how the structure of electrodes impacts EDL properties for decades, a fundamental connection between structural influences on EDL properties and electrocatalytic activity is still missing. Below, we discuss several systems for which the role of electrode structure in EDL properties has been addressed.

### 6.1 Gold (Au)

Although Au has been explored as a catalyst for various reactions,^415−420^ it performs poorly in many key electrochemical processes such as the hydrogen evolution reaction (HER), oxygen reduction reaction (ORR), and carbon dioxide reduction reaction (CO_2_RR). Nevertheless, Au is one of the most investigated systems due to its outstanding electrochemical stability and well-defined EDL potential regions, making it an excellent model system for studying EDL properties.

In 1996, Pajkossy et al.^421^ analyzed anion adsorption on different Au surfaces to explore the origin of the frequency dispersion observed for single-crystal electrodes. They determined interfacial capacitances and rate coefficients in the presence and absence of specific adsorption for the Au(111) and Au(100) single-crystal surfaces using CV- and EIS-based techniques, which are discussed in more detail in section 3. While they did not observe any significant frequency dispersion without specifically adsorbed anions for both Au surfaces, EIS results revealed a difference in the *C*_dl_ as a function of the formal electrode charge density between Au(100) and Au(111). In 0.1 M HClO_4_, as well as with the addition of 0.15 mM NaBr, the *C*_dl_ curves exhibited the same shape, but the absolute values of the *C*_dl_ decreased for Au(100) compared to Au(111). Although no *C*_dl_ minima could be detected in these measurements, and all reported capacitances contain non-negligible contributions of the diffuse layer capacitance, it represents an early example of structure-sensitive double-layer-capacitance studies.

Around the same time, Eberhardt et al.^422^ conducted EIS on Au(100), Au(111), and Au(210) single-crystal surfaces. The simple R-C or R-CPE circuit was chosen for the measurements executed in 0.01 M HClO_4_ electrolyte. However, using a CPE element, they highlighted that the CPE exponent reaches values of up to 0.99, indicating the behavior of an ideal capacitor. Hence, the EDL exhibits minimal frequency dispersion. Before surface reconstruction of the ideal gold surfaces in 0.01 M HClO_4_, the minimum *C*_dl_ of the stable Au(210) surface was identified as the lowest value, followed by the ideal Au(111) surface, while the highest value of the minimum *C*_dl_ was obtained for Au(100). After surface reconstruction of the Au(100) and Au(111) single-crystal surfaces, the minimum *C*_dl_ of Au(100) became smaller than that of Au(111), while Au(210) still demonstrated the smallest *C*_dl_ minimum (*C*_dl_ < 20 μF cm^–2^). In addition, Eberhardt et al. revealed how the electrode potential at which *C*_dl_ reaches its minimum changes due to the differently structured single-crystal surfaces.

These results served as an early validation that the electrode structure influences *C*_dl_ across three surface orientations, including surface reconstruction phenomena. To better understand how these parameters correlate with the activity of an electrochemical reaction, it would be beneficial to consider more suitable materials for electrocatalytic reactions.

### 6.2 Iridium (Ir)

Ir-based catalysts are well-studied in electrochemistry and show good electrocatalytic activities toward multiple reactions, including, for instance, HER, OER, the hydrogen oxidation reaction (HOR), methanol oxidation, ammonia oxidation, formic acid oxidation, ORR, CO_2_RR, and the nitrogen reduction reaction.^423^ We refer to the review article from Huang et al.,^423^ which discusses the catalytic properties of Ir-based catalysts in relation to the mentioned reactions in detail.

Despite being of such electrocatalytic importance, investigations of the structural influence of Ir on EDL properties are scarce. Here, we highlight the work of Pajkossy et al.,^424,425^ who investigated structural effects for Ir(111) and Ir(100) single-crystal surfaces. By performing dEIS (see section 3) with single-frequency capacitance measurements at 1 kHz while scanning the electrode potential, they determined potential-dependent double-layer-capacitance curves for both Ir surfaces in pH-neutral 0.1 M KClO_4_ solution. The *C*_dl_ curves vary in shape and absolute value depending on the surface structure of the Ir single crystal. For instance, Ir(111) exhibits a minimum *C*_dl_ of ∼20 μF cm^–2^ at ∼0.15 V vs SCE, while for Ir(100) they found a higher absolute value of the *C*_dl_ minimum at ∼28 μF cm^–2^ at ∼0.55 V vs RHE.^425^ Due to the limited number of EDL studies for Ir, particularly those examining structural effects, it is challenging to determine any correlations between the EDL structure and electrocatalytic activity. Therefore, more fundamental research about the electrode–electrolyte interface of different Ir surface structures is required.

### 6.3 Copper (Cu)

Cu-based catalysts have received much attention in the context of CO_2_RR due to their unique ability to produce value-added hydrocarbon products with reasonable activity. It was found that the crystal orientation of Cu significantly affects the reaction selectivity toward methane, ethylene, and ethanol production.^426^ However, the complex, multistep charge transfers and the existence of numerous intermediates during CO_2_RR at Cu surfaces raise questions about how exactly reaction pathways and intermediates are connected and what role the EDL and its properties play in this context. From our perspective, a fundamental understanding of how EDL properties and processes correlate with electrocatalytic activity or selectivity is still far from providing any useful insight for such complex reactions as CO_2_RR. Therefore, we focus on more model electrochemical reactions in this section, such as HER and ORR, by discussing the EDL properties of various Cu structures providing important insights into how different adsorption energies and work functions affect EDL properties.

In 2020, Xue et al. investigated the influence of copper single-crystal electrode structures on the minimum *C*_dl_ in alkali metal perchlorate electrolytes (0.05 M MeClO_4_, Me^+^ = Li^+^, Na^+^, K^+^, Rb^+^, Cs^+^) to minimize the influence of hydroxide and hydronium ions on the measurements.^393^ As mentioned in section 5, Xue et al. employed an EEC including adsorption processes from OH* and H* displayed by the adsorption resistance *R*_a_ and capacitance *C*_a_. A more detailed description can be found in section 3. Comparing Cu(111) and Cu(100) revealed that, for each electrolyte, the minimum *C*_dl_ for Cu(100) was higher than that for Cu(111). Interestingly, the electrode potential at which the minimum *C*_dl_ value was observed also changed with the surface structure of the Cu single crystals.

**Figure 23:**
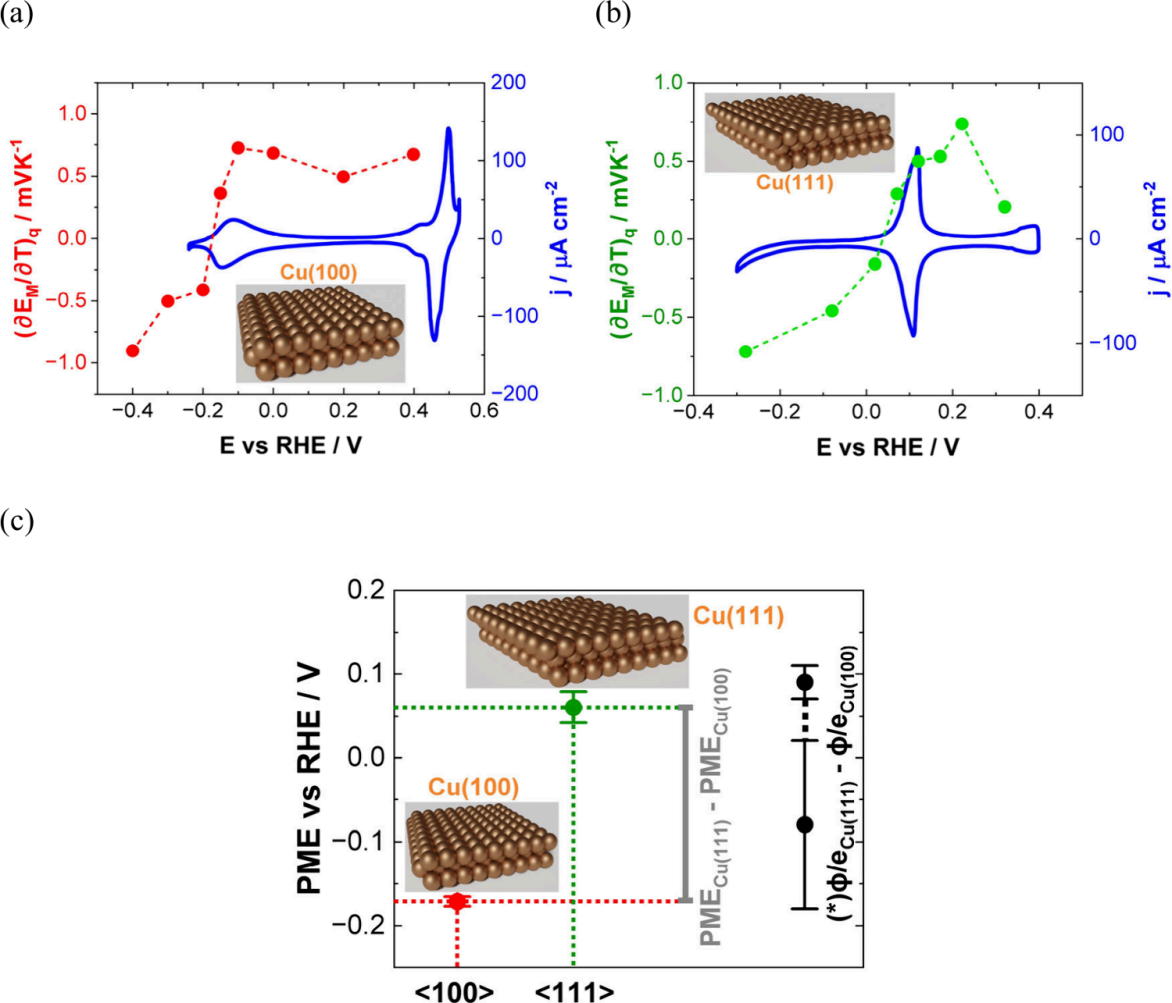
Thermal coefficients (∂*E*_M_/∂*T*) at different applied potentials and corresponding blank CVs of the (a) Cu(100) surface in 0.1 M NaOH solution and (b) Cu(111) surface in 0.1 M NaOH solution. (c) PME for Cu(100) and Cu(111) surfaces in 0.1 M NaOH, plotted against the difference between the values of their work function. Adapted from ref (427). Copyright 2020 American Chemical Society.

Also, Sebastián-Pascual et al.^427^ investigated Cu(111) and Cu(100) in 0.1 M NaOH solution. By applying CV and LITJ, the authors demonstrated that the PME of Cu(111) is higher than that of Cu(100). In this work, the PME was associated with the pzc. Interestingly, the location of the PME values was determined close to the onset peak of adsorbed OH species in the blank CVs and shifts with the pzc value of the Cu single crystal, as shown in Figure 23.

### 6.4 Palladium (Pd)

**Figure 24:**
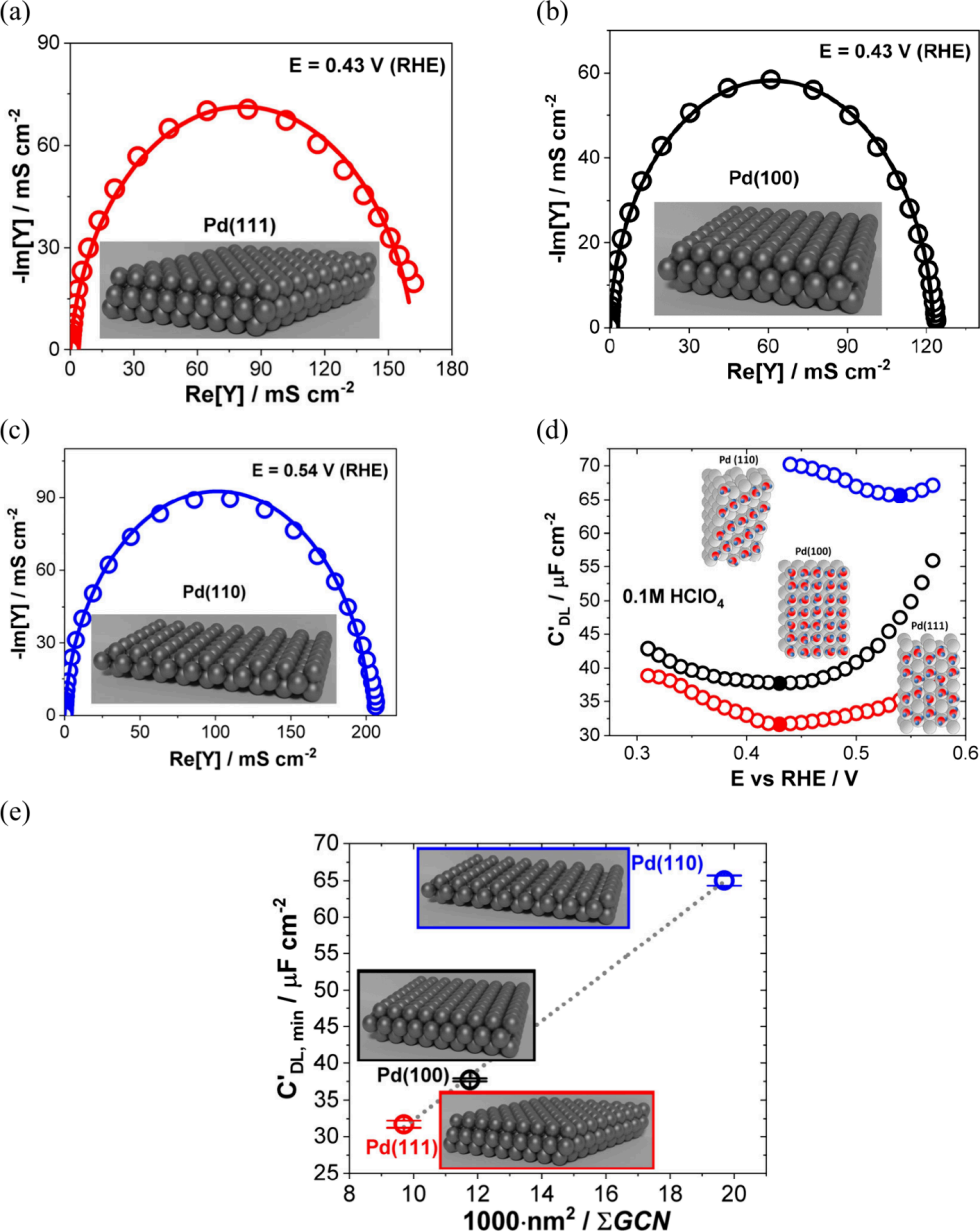
EIS spectra for (a) Pd(111), (b) Pd(100), and (c) Pd(110) at potentials without Faradaic contributions, corresponding to 0.43, 0.43, and 0.54 V, respectively. The solid lines indicate the fit obtained from a classical R-CPE EEC. (d) Extracted *C*_dl_ values obtained for the Pd single crystals with distinct crystal structures for different potentials, measured in 0.1 M HClO_4_. The filled circles indicate the pzc. (e) The respective *C*_dl_ values at the identified pzc as a function of the surface area normalized GCN. Adapted with permission from ref (441). Copyright 2022 American Chemical Society.

Palladium is one of the most widely used materials in catalysis due to its universal application as a catalyst material.^428−440^ In 2022, Gubanova et al.^441^ investigated the *C*_dl_ of basal plane Pd(111), Pd(100), and Pd(110) electrodes. The authors measured the *C*_dl_ values for the three Pd(*hkl*) electrodes in 0.1 M HClO_4_ using EIS, as depicted in Figure 24a–d. In their work, they employed a simple R-CPE model, which was introduced in section 3. Interestingly, the obtained *C*_dl_ values differ substantially as a function of the electrode surface structure, even though all three planes are rather flat model surfaces. When identifying the minimum *C*_dl_ value for each structure, one can observe that not only the absolute values of the minimum *C*_dl_ but also the electrode potential positions at which they occur change. This is an interesting finding since these minima are typically located close to the pzc where the net surface charge is zero. Consequently, the structure of the Pd basal plane influences the double-layer capacitance significantly. To quantitatively describe these results, DFT calculations of water adsorption energies were conducted. To quantify changes in *C*_dl_ upon modifications of the electrode structure, generalized coordination numbers (GCNs) can be used by linking them with the electron density function at the electrode surface. GCNs contain structural information on the electrode surface by accounting for the coordination number of all first-nearest neighbors normalized by the highest individual coordination number of the first-nearest neighbors. With the calculated GCNs for the investigated Pd planes (GCN(Pd_111_) = 7.5 > GCN(Pd_100_) = 6.6 > GCN(Pd_110_) = 5.85), Gubanova et al. found a linear trend between the binding energies for H_2_O molecules and the GCNs of the three Pd planes, as illustrated in Figure 24e. By introducing a new function, which correlates the GCN and the number of accessible surface sites for H_2_O molecules per unit area to the minimum *C*_dl_ as a first approximation, the authors established a quasi-linear relationship showing the effect of electrode structuring on the electrical double layer. They determined minimum *C*_dl_ values of ∼32 μF cm^–2^ for Pd(111), ∼38 μF cm^–2^ for Pd(100), and ∼65 μF cm^–2^ for Pd(110), revealing a strong dependence of *C*_dl_ on the surface structure.

Schmidt et al.^442^ examined the electrocatalytic activity of proton reduction and the HER of the low-indexed basal planes of Pd(111), Pd(110), and Pd(100) in 0.1 M HClO_4_. The activity for the HER followed a trend where Pd(100) was less active than Pd(110), and Pd(111) was the most active. This activity trend does not align with *C*_dl_ or pzc trends observed by Gubanova et al. It raises further questions about the correlation between EDL parameters and electrocatalytic activity. Gubanova et al. measured *C*_dl_ in the double-layer potential region, which characterizes the EDL structure. However, the HER occurs in a different potential region, possibly leading to a different *C*_dl_ trend in the HER Faradaic potential regions. Additionally, Pd is a complex system due to facile hydride formation, which induces strain in the crystal structure and alters the electronic and likely the EDL structure. These results underscore the need for a more detailed understanding of the connections between EDL parameters and electrocatalytic activity.

### 6.5 Platinum (Pt)

Pt-based catalysts are recognized as state-of-the-art electrocatalytic materials that are widely used for many reactions, such as the HER^443,444^ and ORR.^351,443,445−448^ Arguably for this reason, extensive research has been conducted to compare and understand the electrocatalytic properties of Pt. It has been established that its electrocatalytic activity heavily depends on crystallographic orientation and the respective step and terrace sites on the surface. Therefore, changes in EDL properties with step and terrace configurations are crucial to gain a more fundamental understanding of the properties and processes at the electrode–electrolyte interface.

The influence of the step density on the local or overall potential of zero total/free charge was investigated by Gómez et al.^69^ and Climent et al.^232^ for a series of *n*(111) × (111) stepped platinum surfaces in 0.1 M HClO_4_ and 0.5 M H_2_SO_4_ using the CO displacement and N_2_O reduction methods. For example, Gómez et al. observed a decrease in the pztc from ∼0.3 V vs RHE for Pt(10 10 9) to ∼0.2 V vs RHE for Pt(221) with increasing step density, demonstrating the strong dependency of electron charge density redistributions on the structure of electrode surfaces.^69^ These results were later further corroborated by another study by Climent et al., which gave special attention to the local determination of the pzc via CO charge displacement and N_2_O reduction and the approximation of the pzfc of stepped platinum surfaces vicinal to Pt(111).^449^

**Figure 25:**
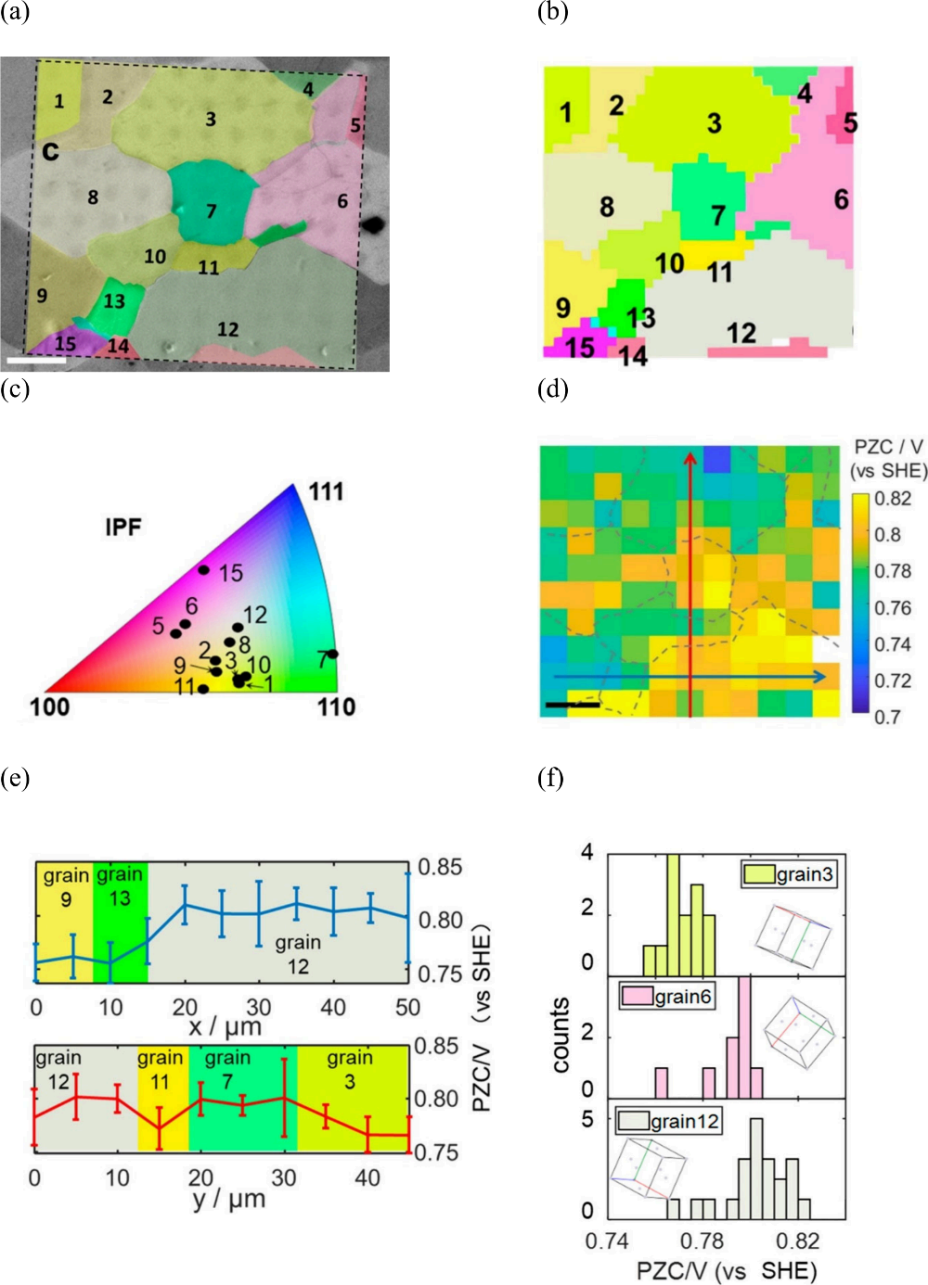
(a) SEM image visualizing distinct grains, which are colored and numbered, respectively. The scale bar in the image corresponds to 10 μm. (b) EBSD map and (c) IPF providing the crystallographic orientations of the respective grains. (d) Determined pzc values on the respective grains through SECCM experiments (repetitive approach method). (e) Line scans showcasing the pzc shift depending on the respective grain. The blue and red line scans correspond to the positions of the arrows drawn in (d). (f) pzc distribution determined for grains 3, 6, and 12, respectively. Reprinted with permission from ref (74). Copyright 2020 American Chemical Society.

Another study, which correlated the pzc of the Pt surface with distinct crystallographic orientation, was conducted by Wang et al.^74^ Their study provided new insights into method development to determine EDL properties by utilizing an innovative SECCM-based method., which was briefly described in section 3 and already discussed in the work of Kim et al. in section 5. Their experimental procedure included 1 μm diameter pipet tips for the repetitive approaching procedures at different grains on a Pt(pc) surface, which were characterized by SEM images and electron backscatter diffraction (EBSD) measurements displayed in Figure 25, parts a and b, respectively. With the help of the inverse pole figure (IPF) (Figure 25c), the crystallographic orientations of the grains were further identified. The resulting pzc map is illustrated in Figure 25d, revealing values ranging from 0.7 to 0.82 V vs SHE in the employed 1 mM HClO_4_ filling electrolyte. Line scans (red and blue color) were extracted and are displayed in Figure 25e, corresponding to the 1D arrows shown in the pzc map. The line scans further indicate the drastic difference arising in the pzc with distinct grains. A more detailed analysis of the results is provided by comparing the pzc distribution across three representative grains, as displayed in Figure 25f. It highlights that the crystal orientation of grain 3, which is closely aligned with the 100 and 110 directions, exhibits the lowest pzc at 0.77 V vs SHE. Conversely, grain 6, oriented closer to the 100 and 111 directions, shows a slightly higher pzc of 0.79 V vs SHE, while grain 12, positioned closer to the 111 and 110 orientations, demonstrates the highest pzc of 0.81 V vs SHE. The authors noted that the measured pzc values are relatively high compared to those derived from capacitance measurements or CO displacement methods^450^ but align more closely with the values obtained through immersion methods.^451^ Additionally, the study explored the relationship between pzc and various parameters related to the HER activity, like current densities, transfer coefficients, standard rate constants, and Tafel slopes in solutions containing 10 mM HClO_4_ and 10 mM KCl.

In summary, the use of single crystals is essential to investigate the influence of electrode structure on EDL properties. This allows for a clearer correlation between different factors such as step densities, terrace sites, surface-normalized generalized coordination numbers, and global adsorption energies with EDL properties. Nevertheless, studying single crystals is quite a formidable task since their preparation and general treatment under experimental conditions is challenging. Considering these points, the presented SECCM-based approach proves very promising. This is because a polished, polycrystalline material consisting of multiple grains with different crystallographic orientations can be used. However, the crystal orientations of the exposed grains likely do not correspond to high-index, kinked stepped surfaces. Furthermore, as mentioned by Wang et al.,^74^ deviation of the pzc values compared to well-known methods like CO displacement arises, emphasizing the early stage of development of SECCM-based approaches. More extensive experimental work in the future should improve the accuracy and range of application of this technique and thus help to elucidate previously found discrepancies.

**Figure 26:**
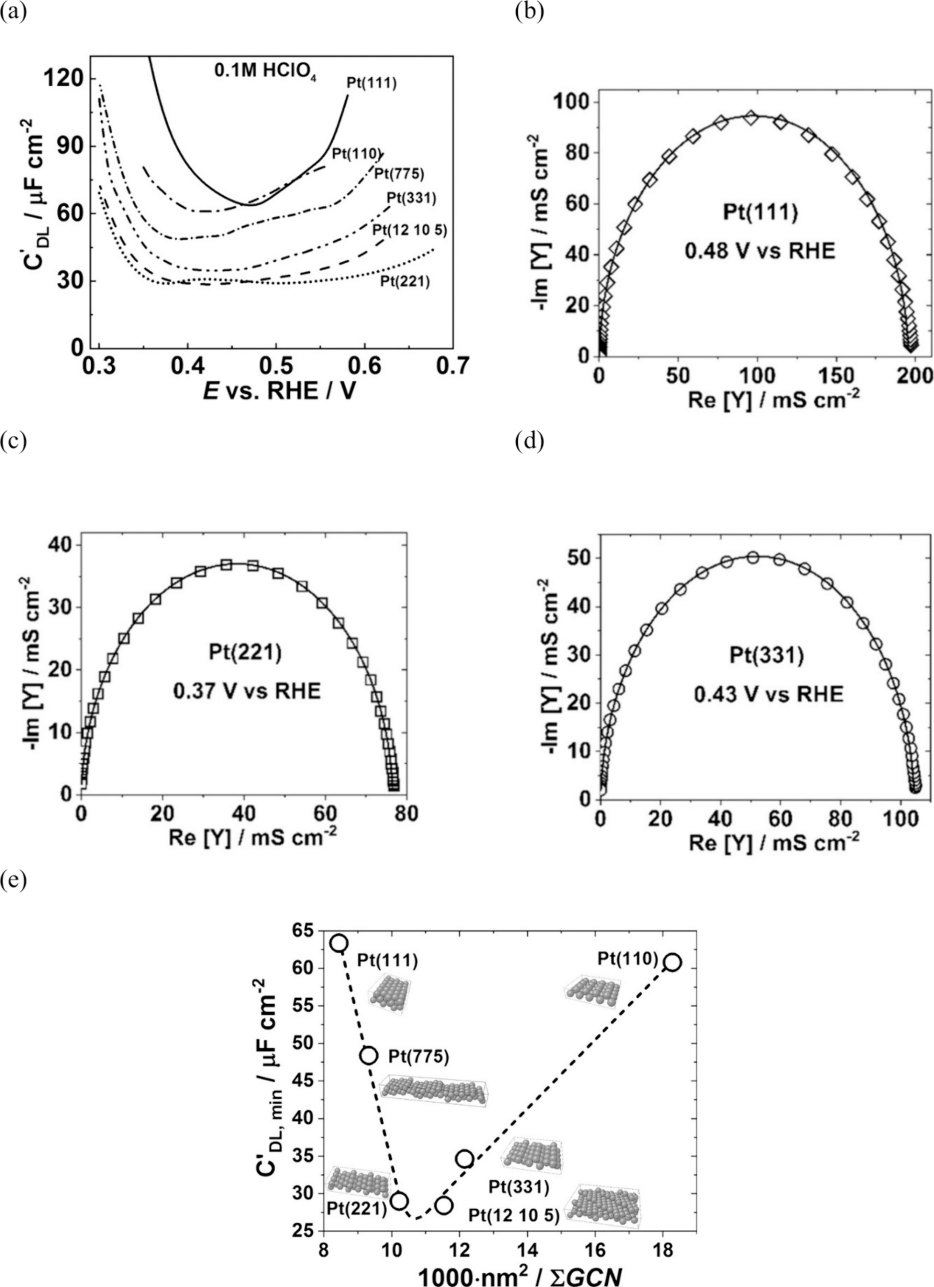
(a) *C*_dl_ measured in 0.1 M HClO_4_ for Pt single crystals including (111), (110), (775), (331), (12 10 5), and (221) crystal orientations. (b–d) Exemplary impedance spectra of Pt(111), Pt(221), and Pt(331) measured at 0.48, 0.37, and 0.43 V vs RHE, respectively. (e) Dependency of the minimal *C*_dl_ determined at the pzc versus the surface area normalized GCN for the previously mentioned Pt(*hkl*) single crystals. Reprinted with permission from ref (452). Copyright 2024 American Chemical Society.

So far, we have discussed the shift of the pzc depending on the crystallographic orientation and, therefore, on different step and terrace sites and densities. In the following, we highlight some studies that focused on *C*_dl_ variations for different single-crystal surfaces. Xue et al.^393^ measured the minimum *C*_dl_ for model Pt(111), stepped Pt(775), and kinked Pt(12 10 5) single-crystal surfaces in various electrolytes (0.05 M MeClO_4_, Me^+^ = Li^+^, Na^+^, K^+^, Rb^+^, Cs^+^) via EIS. Again, in this study, an EEC was used to account for adsorption processes from OH* and H* displayed by the adsorption resistance *R*_a_ and capacitance *C*_a_. They observed that the minimum *C*_dl_ values decreased with the increasing number of steps and kinks at the surface, which resulted in the highest minimum *C*_dl_ obtained for Pt(111), followed by Pt(775), and the lowest minimum *C*_dl_ obtained for Pt(12 10 5) for each electrolyte used in the experiment. Interestingly, the same trend was observed when the electrolyte was replaced by 0.1 M HClO_4_. Furthermore, in 2023, Xue et al.^452^ extended their structure-dependent EDL research by expanding the set of crystallographic orientations to Pt(111), Pt(775), Pt(221), Pt(12 10 5), Pt(331), and Pt(110). The *C*_dl_ dependencies on the potentials in 0.1 M HClO_4_ are displayed in Figure 26a accompanied by exemplary impedance spectra of Pt(111), Pt(221), and Pt(331), shown in Figure 26b–d. The study employed a simple R-CPE EEC due to the absence of specifically adsorbing ions. The derived *C*_dl_ minima were further analyzed and plotted against the surface area normalized GCN, revealing an inverse volcano-type correlation that underscored the significant impact of surface structure on the *C*_dl_ (Figure 26e). Notably, a drastic contrast in *C*_dl_ values was observed between Pt(111) and Pt(110) (around 60 μF cm^–2^) compared to Pt(221) and Pt(12 10 5) (approximately 28 μF cm^–2^), which shows that surface structural effects like steps, terraces, kinks, and their degree of occurrence strongly affect the double-layer structure and dynamics. To explore the origin of such deviations in EDL properties, AIMD simulations were carried out to provide deeper insights into the orientation of water molecules at different Pt surface structures and their dependence on crystallographic orientations, which will be more thoroughly discussed in the dedicated theoretical section, section 6.6.

The study by Xue et al. provides fundamental groundwork for exploring whether there is a correlation between surface-structure-dependent *C*_dl_ and electrocatalytic activity. We refer to several studies from Hoshi and co-workers, who conducted several ORR activity measurements for many basal, stepped, and kinked Pt single crystals. Pt(110), Pt(111), and Pt(775) exhibited smaller ORR activities compared to Pt(331) or Pt(221) in 0.1 M HClO_4_,^453,454^ which could be a first indication of a correlation between activity and the determined *C*_dl_, depending on the surface normalized GCN. Nevertheless, to develop a suitable theory of such a possible correlation, experimental studies should be conducted under the same conditions to ensure the highest level of reproducibility. Simultaneously, experimental findings should be complemented by theoretical calculations to verify and test possible relationships between EDL properties and electrocatalytic activity parameters.

After our discussion about the influence of the electrode structure on the pzc and *C*_dl_, we now turn to the studies of the PME. Garcia-Araez et al.^119^ investigated the structural effects of the Pt(111), Pt(100), and Pt(110) single-crystal surfaces by investigating the pzc via CO displacement and the PME via laser-induced jump technique experiments in 0.1 M KClO_4_ with an optional addition of HClO_4_ to change the electrolyte pH. The authors obtained different pztc’s for different single-crystal surfaces located within the double-layer region, somewhat independently of the electrolyte pH. Pt(111) was found to exhibit the highest pztc value, followed by Pt(100) and Pt(110), even though the pztc of Pt(111) overlaps with the one of Pt(100) for lower pH. Furthermore, all three single-crystal electrodes exhibited different PME values independently of the electrolyte pH, with Pt(111) having the highest value, followed by Pt(100) and Pt(110). Garcia-Araez et al. explained the relative difference in PME values with the difference in the work function values for the three basal planes. This finding indicates a clear correlation between electrode structure and double-layer formation. However, CO displacement and laser-induced jump technique measurements are known to be sensitive to charge transfer processes and water–metal interactions, respectively, which complicates the precise explanation of the structural effect of Pt(111), Pt(100), and Pt(110) on the water reorientation within the EDL. Furthermore, Martínez-Hincapié et al. found that PME and pztc significantly differ for the three basal planes of platinum in the mixture of KClO_4_ and HClO_4_ in the pH range from 1 to 3.^111^ It was demonstrated that pzc and PME have very close values for Pt(111), and they differ significantly for Pt(100) and Pt(110). Besides, the PME values were found to be located at lower potentials than the pztc.

**Figure 27:**
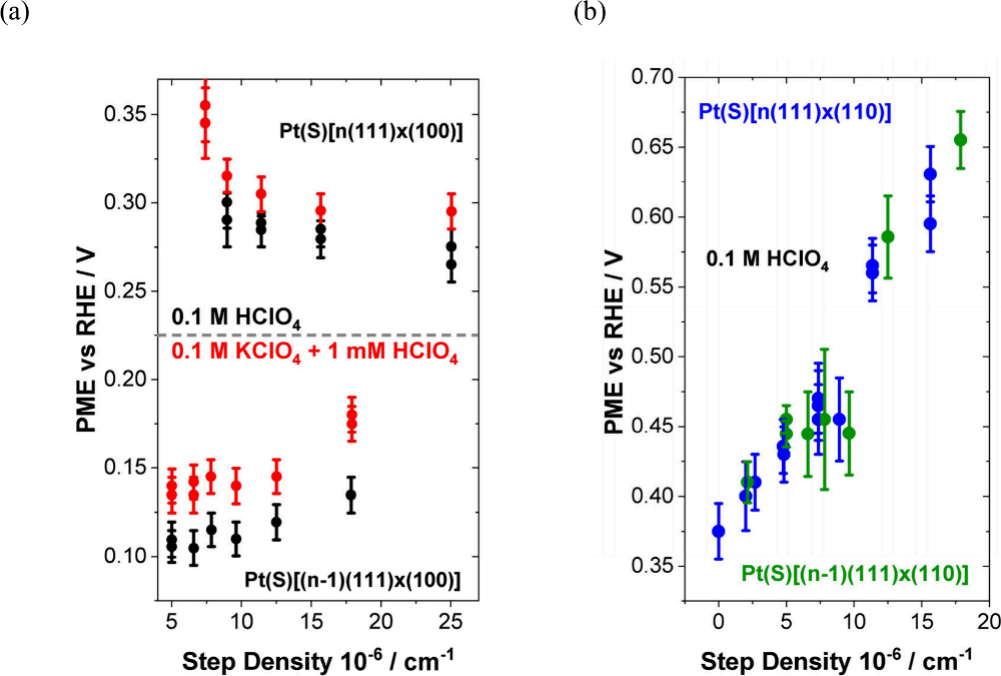
(a) PME related to step sites as a function of step density in 0.1 M HClO_4_ (black points) and 0.1 M KClO_4_ + 1 mM HClO_4_ (red points) on different Pt(S)[(*n* – 1)(111) × (110)] and Pt(S)[*n*(111) × (100)] surfaces. (b) PME related to terrace sites as a function of step density in 0.1 M HClO_4_ on Pt(S)[(*n* – 1)(111) × (110)] (green points) and Pt(S)[*n*(111) × (100)] (blue points) surfaces. Adapted with permission from ref (455). Copyright 2008 Elsevier.

Similar studies were conducted for structurally more complex Pt single-crystal surfaces. For instance, García-Aráez et al.^455^ identified the PMEs for various stepped Pt surfaces with (111) terraces in 0.1 M HClO_4_ and 0.1 M KClO_4_ + 1 mM HClO_4_ electrolytes, depending on the step density of the investigated surface. The first PME, which is demonstrated in Figure 27a, was found in the hydrogen adsorption potential region and was related to the step sites. The location of the second PME, shown in Figure 27b, was identified within the double-layer region and corresponds to terrace sites. The latter allowed one to avoid the contribution to the potential transients of the kinetics of hydrogen or OH adsorption. Remarkably, the second PME significantly increases with the step density.^455^ Following up the Pt stepped single crystal studies of García-Aráez et al., Sarabia et al. elucidated the change of the net orientation of water dipoles for Pt single-crystal surfaces with (111) terraces and (110) steps.^117^ Various stepped surfaces with increasing step density in the order Pt(10 10 9) > Pt(554) > Pt(775) > Pt(553) were examined in 0.1 M NaOH. Compared to the model Pt(111) surface, the recorded laser transients again revealed the existence of two PMEs, one of which is located in the double-layer region and the other is located at ∼0.25 V vs RHE, where hydrogen adsorption to the (110) step takes place. According to Sarabia et al., this corresponds to the existence of two pzfc’s: one related to the steps and the other to the terraces. At these potentials, a local electric field change induces disorder and rearrangement of water molecules in the solid–liquid interface. While the PME associated with the steps stays relatively constant with the step density, the PME associated with the terrace sites decreases with increasing step density. According to their hypothesis, hydroxyl anions located closely on the electrode surface change the preferential orientation of the water molecules at the electrode surface. The increasing number of steps at the surface causes a disruption of the water molecule network, leading to a shift of the PMEs to lower values. Another explanation for the structure sensitivity of the PME could be that the adsorption of hydroxyl anions is more favored at step sites, resulting in an imaginary positive charge at the terrace sites, affecting the orientation of the water molecules with the oxygen atom pointing toward the electrode surface. Consequently, according to the authors, a more negative potential is necessary to induce the rearrangement of the water molecule network.

While high-index stepped Pt surfaces exhibit two distinct PMEs, correlated to step or terrace sites, with opposing trends with the surface step density depending on the used electrolyte, it is not straightforward to correlate these PMEs with electrocatalytic activity. The hypothesis suggests that the potential difference between the PME and the equilibrium potential of an electrochemical reaction is the key parameter in predicting electrocatalytic activity. This implies that the electrocatalytic activity at the step or terrace sites may change independently as the step density increases. Nevertheless, the studies mentioned above established the fundamental basis for further activity measurements and theoretical predictions required to validate the correlation of activity with the respective PME of terraces or steps.

In conclusion, the observed dependencies of EDL parameters, such as *C*_dl_, pzc, and PME, on distinct surface structures within a specific material composition were linked to changes in adsorption properties or work functions due to dissimilar crystallographic orientations and step densities. Our current understanding of the EDL is improving because of the investigation of more complex systems. For example, high-indexed, stepped single crystals were found to have two distinct PMEs, corresponding to terrace and step sites with different trends upon step density and electrolyte. Nevertheless, we strongly suggest that EDL characterizations should be combined directly with activity measurements for selected electrochemical reactions to better understand the correlation between PME, pzc, and *C*_dl_ and their activities. Particularly, the PME seems to have a direct correlation with electrochemical reaction activity. The closer the PME lies to the equilibrium potential of an electrochemical reaction, the larger the reaction activity. However, identical experimental conditions are mandatory to prevent errors, such as the electrolyte’s influence on EDL properties and electrochemical activities, a topic to be further discussed in section 7. Furthermore, the structural effects have been investigated mainly for one-composite noble or base metals. However, future research needs to extend to more complex compounds like bimetallic alloys, metal chalcogenides, ternaries, or high entropy alloys, which constitute promising candidates as electrocatalytic materials for future energy applications. A summary of selected EDL parameters discussed within this study is provided in Tables 3 and 4.

**Table 3:** Selected Representative Studies Exploring Variations in *C*_dl_ Values Based on Electrode (Sub)surface Structure^a^

structure	*C*_dl_	potential	method	electrolyte	ref
Ir(111)	20 μF cm^–2^	∼0.15 V vs SCE	dEIS	0.1 M KClO_4_	(424)
Ir(100)	28 μF cm^–2^	∼0.55 V vs RHE	dEIS	0.1 M KClO_4_	(425)
Cu(111)	(25 ± 2) μF cm^–2^	pzc	EIS	0.05 M LiClO_4_	(393)
Cu(100)	28 μF cm^–2^	pzc	EIS	0.05 M LiClO_4_	(393)
Cu(111)	(31 ± 1) μF cm^–2^	pzc	EIS	0.05 M NaClO_4_	(393)
Cu(100)	33 μF cm^–2^	pzc	EIS	0.05 M NaClO_4_	(393)
Cu(111)	(35 ± 2) μF cm^–2^	pzc	EIS	0.05 M KClO_4_	(393)
Cu(100)	(37 ± 2) μF cm^–2^	pzc	EIS	0.05 M KClO_4_	(393)
Cu(111)	(38 ± 10) μF cm^–2^	pzc	EIS	0.05 M RbClO_4_	(393)
Cu(100)	(43 ± 10) μF cm^–2^	pzc	EIS	0.05 M RbClO_4_	(393)
Cu(111)	(38 ± 5) μF cm^–2^	pzc	EIS	0.05 M CsClO_4_	(393)
Cu(100)	(43 ± 5) μF cm^–2^	pzc	EIS	0.05 M CsClO_4_	(393)
Pd(111)	(32 ± 0.5) μF cm^–2^	pzc	EIS	0.1 M HClO_4_	(441)
Pd(100)	(38 ± 0.5) μF cm^–2^	pzc	EIS	0.1 M HClO_4_	(441)
Pd(110)	(65 ± 0.7) μF cm^–2^	pzc	EIS	0.1 M HClO_4_	(441)
Pt(111)	63 μF cm^–2^	pzc	EIS	0.1 M HClO_4_	(452, 393)
Pt(775)	48 μF cm^–2^	pzc	EIS	0.1 M HClO_4_	(452, 393)
Pt(221)	29 μF cm^–2^	pzc	EIS	0.1 M HClO_4_	(452)
Pt(12 10 5)	28 μF cm^–2^	pzc	EIS	0.1 M HClO_4_	(452, 393)
Pt(331)	35 μF cm^–2^	pzc	EIS	0.1 M HClO_4_	(452)
Pt(110)	61 μF cm^–2^	pzc	EIS	0.1 M HClO_4_	(452)

a The table columns list the material (sub)surface structures, corresponding *C*_dl_ values, potentials at which *C*_dl_ values were determined, methods used, electrolytes employed, and literature references. It should be noted that several *C*_dl_ values are estimated from the original published data. This table serves as a comprehensive overview of the cited literature.

**Table 4:** Selected Representative Studies Exploring Variations in pzc/PME Values Based on Electrode (Sub)surface Structure^a^

structure	potential	parameter	method	electrolyte	ref
Cu(111)	(0.06 ± 0.02) vs RHE	PME	LICT	0.1 M NaOH	(427)
Cu(100)	(−0.17 ± 0.01) vs RHE	PME	LICT	0.1 M NaOH	(427)
Pd(111)	0.43 V vs RHE	pzc	EIS	0.1 M HClO_4_	(441)
Pd(100)	0.43 V vs RHE	pzc	EIS	0.1 M HClO_4_	(441)
Pd(110)	0.54 V vs RHE	pzc	EIS	0.1 M HClO_4_	(441)

a The table columns list the material (sub)surface structures, potentials at which the respective parameters were determined, which parameter was explored (pzc/PME), methods used, electrolytes employed, and literature references. It should be noted that several pzc/PME values are estimated from the original published data. This table serves as a comprehensive overview of the cited literature.

### 6.6 DFT Simulations

It is important to note that most experiments are performed with nanoparticles, while computational studies often focus on atomically flat metal surfaces as simpler model systems. To bridge this gap, a series of ab initio investigations has recently analyzed basic EDL characteristics as a function of the electrode structure.^456−458^ For instance, in a recent computational investigation,^456^ a series of flat (111, 100, and 001) and stepped (211) transition metal–water interfaces were examined employing both static and molecular dynamics DFT calculations. It was shown that the *U*_pzc_ of (211) facets are 0.1–0.6 V_SHE_ lower than those of (111), depending on the transition metal. However, in another AIMD study,^457^ a more positive pzc (by ∼0.3 V) for the Pt(553)/water interface than for the Pt(111)/water interface was reported. A recent AIMD investigation focused on the analysis of the pzc across five Pt stepped surfaces (311, 211, 533, 433, and 544) that provide different step densities.^458^ It was demonstrated that the pzc’s of stepped Pt facets are generally smaller than that of Pt(111), and the difference was primarily attributed to the difference in their work functions. Despite some discrepancies between various theoretical studies, it appears that water molecules prefer to chemisorb at step sites of metal surfaces but physisorb at terrace sites. This, in turn, should have important implications for the double-layer capacitance, as it is more difficult to reorient chemisorbed water molecules.

In another recent AIMD-based study,^452^ a series of Pt single crystals with various crystallographic orientations were examined to elucidate the relationship between the surface structure and the double-layer capacitance. Simulations were aimed at explaining a drastic contrast in measured *C*_dl_ values between Pt(111) and Pt(110) (∼60 μF cm^–2^) and Pt(221) and Pt(12 10 5) (∼28 μF cm^–2^) facets. In agreement with previous AIMD studies of stepped Pt facets discussed above, it was also observed that water molecules prefer to physisorb at terrace sites and chemisorb at step sites. However, the interfacial water structure turned out to depend nonlinearly on the step density. Specifically, it was observed that surfaces with low step densities, such as Pt(111), are indeed characterized by preferentially physisorbed water molecules leading to high measured *C*_dl_ values. When the step density increases, the fraction of chemisorbed water molecules becomes greater, resulting in higher rigidity of the EDL, as reflected by lower average dipole moments of water. This was found to be consistent with substantially lower measured *C*_dl_ values for Pt(221) and Pt(331) facets. However, upon further increase of the step density, such as in the case of Pt(110), the disruption of the water structure due to chemisorbed water on the sites of close proximity becomes more pronounced. This is indicated by higher average water dipole moments calculated using AIMD trajectories agreeing with higher *C*_dl_ values estimated experimentally.

To obtain a more complete picture of the EDL properties as a function of electrode surface structure, further microscopic interfacial studies are warranted. In this regard, AIMD-based investigations, despite their time- and length-scale limitations, are well poised to provide useful mechanistic information. These investigations, however, should also go beyond monometallic surfaces, such as Pt, and also analyze the role of the electrode potential.

## 7 The Role of the Electrolyte Composition

It is well-known that the interfacial environment between electrode and electrolyte also depends on the electrolyte composition. The electrolyte effect includes the ions directly involved in an electrochemical reaction and those that do not participate, known as spectator electrolyte ions, such as alkali metal (AM) cations.^413,459−462^ Both types of ions interact with the electrode catalyst in distinct ways, and numerous studies have confirmed the significant role of even spectator ions in influencing the double-layer structure and properties. The impact of the electrolyte composition can be categorized based on the type of ions and their concentration, thus, the resulting pH value. In principle, a larger density of ions emerges near the electrode surface compared to the bulk.^198^ The IHP and the OHP (Stern layer) consist of specifically adsorbed ions and solvated ions nearest the electrode, respectively. At the same time, the diffuse layer is comprised of nonspecifically adsorbed ions driven by the long-range electrostatic force. The electrostatic interactions at the interface induced by adsorbed species and charging on the electrode surface can strongly alter the double-layer structure, particularly the water layer structure. The species may adsorb on the electrode surface when their tendency to interact with the electrode surpasses the one with the solvent. Instead of specific adsorption on the electrode surface, spectator cations in the electrolyte tend to interact with the reaction intermediate and electrode through noncovalent bonds.^463,464^

In this section, we will elucidate the influence of cation size, hydration energy, electronegativity, and the noncovalent interactions between hydrated cations and intermediate species. Our discussion underscores that those properties modulate the interfacial water environment and the EDL structure.^413,462,465^

### 7.1 The Nature of Alkali Metal Cations

First, we will guide the readers through recent advances in studying the influence of alkali metal cations on EDL properties. This means that these studies do not explore the influence of cation concentrations and electrolyte pH, which will be discussed later.

**Figure 28:**
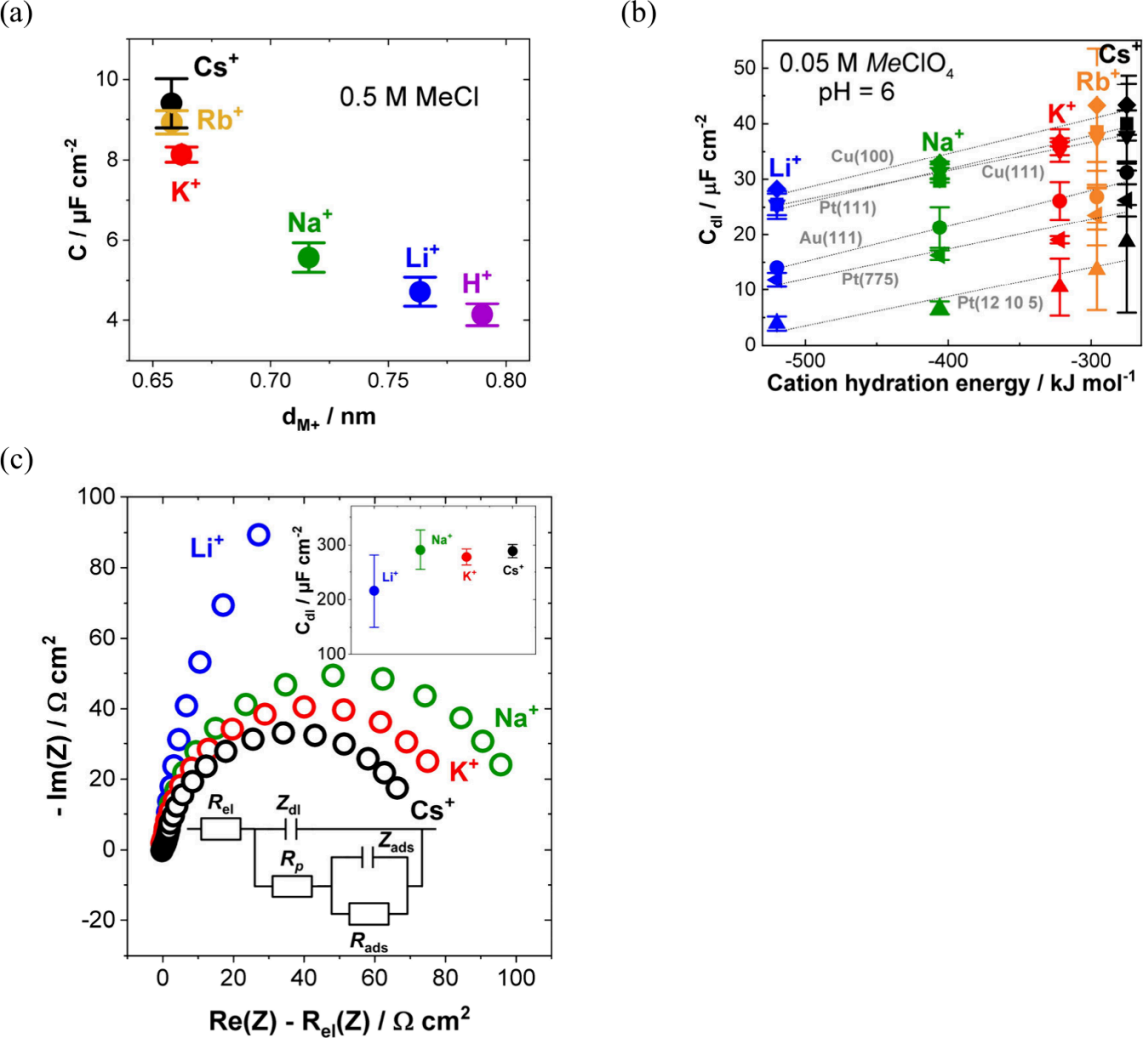
(a) Areal capacitances of basal-plane HOPG determined in 0.5 M MeCl electrolytes, containing different cations.^466^ (b) *C*_dl_ values obtained at the pzc for single crystals with different crystal structures, including Cu(100), Cu(111), Pt(111), Pt(775), Pt(12 10 5), and Au(111) in 0.05 M MeClO_4_ electrolytes with different cations. The *C*_dl_ values are plotted against the hydration energy of the differently employed ion.^393^ (c) Nyquist plot obtained from EIS of an NiOOH surface using the corresponding equivalent circuit. The inset in (c) displays the obtained *C*_dl_ values at the pzc obtained from fitting the EIS spectra.^468^ (a) Adapted from ref (466). Copyright 2019 American Chemical Society. (b) Adapted from ref (393). Copyright 2020 American Chemical Society. (c) Adapted with permission from ref (468). Copyright 2019 Wiley-VCH-Verlag GmbH & Co. KGaA.

For example, distinctions in EDL properties depending on the nature of the alkali metal cation were reported by Iamprasertkun et al.,^466^ who analyzed the capacitance properties of 2D semimetallic highly ordered pyrolytic graphite (HOPG). HOPG is an excellent candidate for EDL studies due to its simple and well-known characteristics.^467^Figure 28a depicts the capacitances of the HOPG basal plane in metal chloride electrolytes (AMCl, AM^+^ = H ^+^, Li ^+^, Na ^+^, K ^+^, Rb^+^, and Cs^+^) with a concentration of 0.5 M, measured using EIS at 0 V vs Ag/AgCl. The acquired data suggest that the capacitances vary with the cation size. While larger cations such as Cs^+^, Rb^+^, and K^+^ feature similar *C*_dl_ values, the *C*_dl_ values progressively decrease for Na^+^, Li^+^, and H^+^. Iamprasertkun et al. attributed this trend to an increase in hydration energy difference across metal cations that leads to a more expansive hydration shell. This finding seems to be not only valid for carbon-based materials, like HOPG, since Xue et al.^393^ reported analogous results only a year later, in 2020, for a variety of metal single crystals. Their work involved EIS measurements (Figure 28b) in 0.05 M AMClO_4_ (AM^+^ = Li ^+^, Na ^+^, K ^+^, Rb ^+^, Cs^+^) on the capacitances of various single-crystalline electrode materials and structures, including Cu(100), Cu(111), Pt(111), Au(111), Pt(775), and Pt(12 10 5). The employed EEC was discussed in section 3 in more detail and mentioned in sections 5 and 6. However, we highlight again that, due to adsorption processes, both *R*_a_ and *C*_a_ are connected in parallel to the CPE, which describes the EDL. The capacitance linearly depends on the cation hydration energy regardless of the single crystal and its structure. Additionally, they reported that most cations are accumulated in the diffuse layer region, with concentrations near the electrode being ∼80 times greater than that in the bulk.

The results from both studies by Iamprasertkun et al. and Xue et al. underscore the significant effect of cation characteristics on EDL properties. Remarkably, these studies found a consistent linear correlation between *C*_dl_ and the size/hydration energy of cations across three distinct classes of materials: carbon-based HOPG, noble metals like Au and Pt, and base metals such as Cu. The correlation also remains established across different crystallographic orientations of the single crystals used in experiments. Despite these insights, all the systems examined so far correspond to relatively simple model systems, which raises the question of whether cations similarly affect EDL properties for more complex systems. For this reason, we guide the readers through the work of Garcia et al.,^468^ who explored the influence of the alkali metal cation within AMOH electrolytes (AM^+^ = Li^+^, Na^+^, K^+^, Cs^+^) on the nickel oxyhydroxide (NiOOH) surface. The respective *C*_dl_ values were determined via EIS in different AMOH electrolytes at 1.6 V_RHE_. Due to the larger complexity of their electrochemical system, an Armstrong–Henderson equivalent electric circuit^469^ was used for the EIS fitting, which is displayed in Figure 28c. *R*_el_ corresponds to the electrolyte resistance, and *Z*_dl_ corresponds to the CPE modeling the electrochemical double layer. Furthermore, the parallel branch describes additional processes related to a charge transfer of a Faradaic reaction (*R*_p_) and the respective adsorption resistance (*R*_ads_) and capacitance (*Z*_ads_). Figure 28c also shows the EIS results of an NiOOH layer, which are similar to those of other groups.^470^ The results indicate that the employed cations do not significantly alter the *C*_dl_, particularly for larger cations like Na^+^, K^+^, and Cs^+^, although there is a tendency for a decrease for the smaller cation Li^+^. These findings suggest that the cation size and hydration energy in more complex systems like NiOOH do not follow the ideal linear trend with *C*_dl_ observed in the model systems studied by Iamprasertkun et al. and Xue et al. The absence of the cation trend for more complex catalyst systems is challenging to explain with our limited understanding of the EDL. Therefore, it is crucial to continue investigating such complex systems. The ongoing research will be essential in refining our theoretical models and generally enhancing our understanding of the EDL.

**Figure 29:**
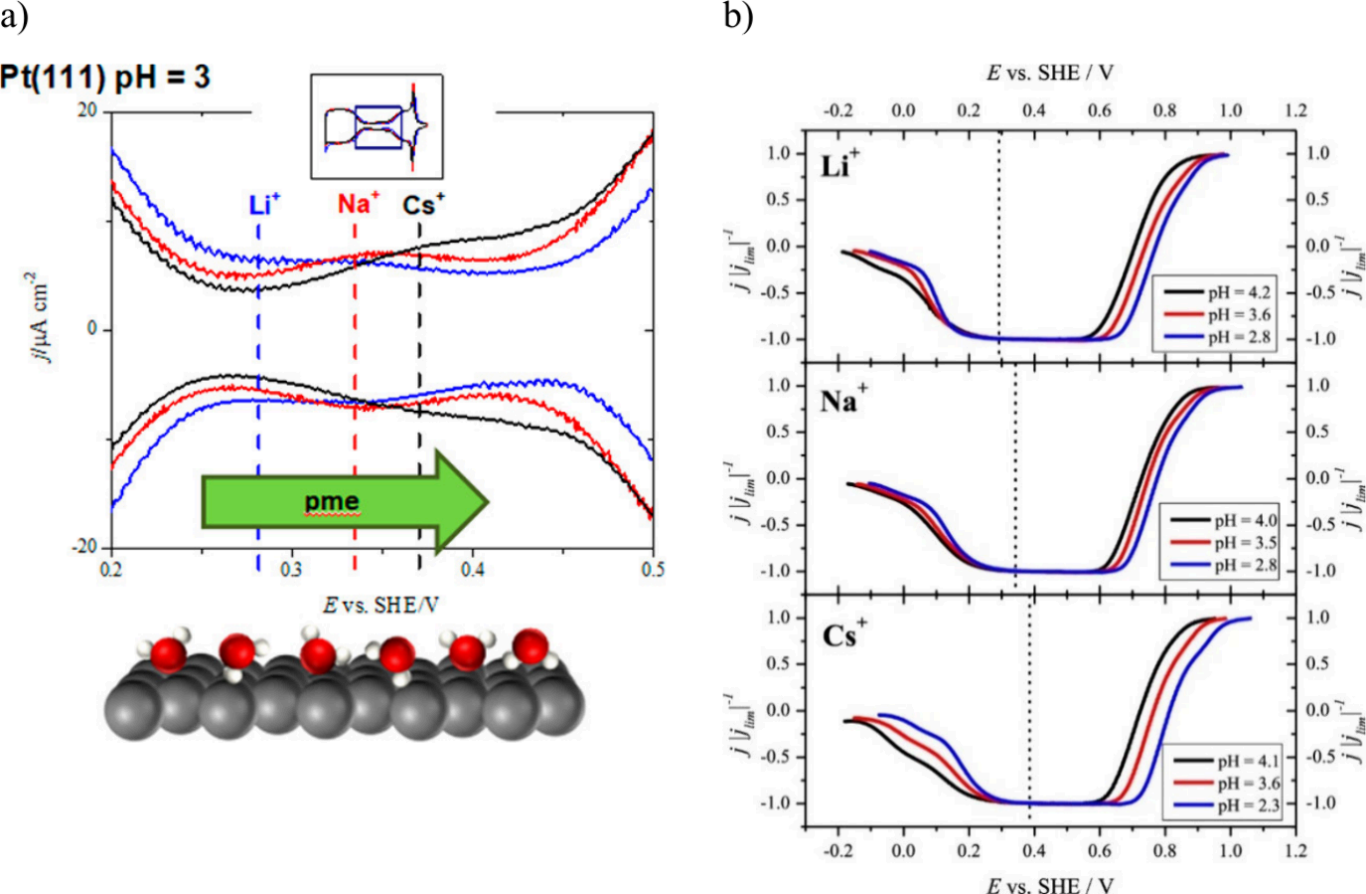
(a) The interface between the Pt(111) electrode surface and various MeF/HClO_4_ (Me^+^ = Li^+^, Na^+^, or Cs^+^) solutions are explored. The PME values increase with cations: PME(Li^+^) < PME(Na^+^) < PME(Cs^+^). (b) The polarization curves for the HPRR and HPOR on Pt(111) in Ar-saturated MeF/HClO_4_ mixtures (Me^+^ = Li^+^, Na^+^, Cs^+^). Measurements were conducted with 1.7 mM H_2_O_2_ at various pH levels in the range from 2.3 to 4.1 using the rotation rate 2500 rpm and the scan rate 50 mV s^–1^. The presence of vertical dashed lines denotes *E*_inhibition_ for each cation. Reprinted from ref (471). CC BY 4.0.

Despite highlighting several studies focused on the dependency of *C*_dl_ on the nature of the cation, the question arises of how these trends can be correlated with the activity of an electrochemical reaction. Therefore, electrochemical testing must be performed for the respective single-crystal surfaces under identical experimental conditions compared to the EDL research conducted. On the other hand, for the PME research, correlating the nature of cations in an electrolyte with the electrocatalytic activity of an electrochemical reaction seems to be way more common. For instance, Briega-Martos et al.^471^ explored the effect of the alkali metal cation on the PME of the Pt(111) surface using MeF/HClO_4_ (Me^+^ = Li^+^, Na^+^, or Cs^+^) aqueous electrolytes across a pH range of 1–6. Choosing the electrolyte composition allowed investigations without specific adsorption of anions. The following PME trend was demonstrated: PME(Li^+^) < PME(Na^+^) < PME(Cs^+^) (Figure 29a). In addition, activity measurements were performed toward the hydrogen peroxide reduction reaction (HPRR), particularly focusing on the potential at which the inhibition of the HPRR takes place on Pt(111) (*E*_inhibition_) (Figure 29b). The study demonstrated that variations in *E*_inhibition_ depend on the nature of the alkali metal cations in the solution, following the sequence *E*_inhibition_(Cs^+^) > *E*_inhibition_(Na^+^) > *E*_inhibition_(Li^+^). Interestingly, this order aligns with the trend of the PME observed for different cations in the respective electrolyte.

**Figure 30:**
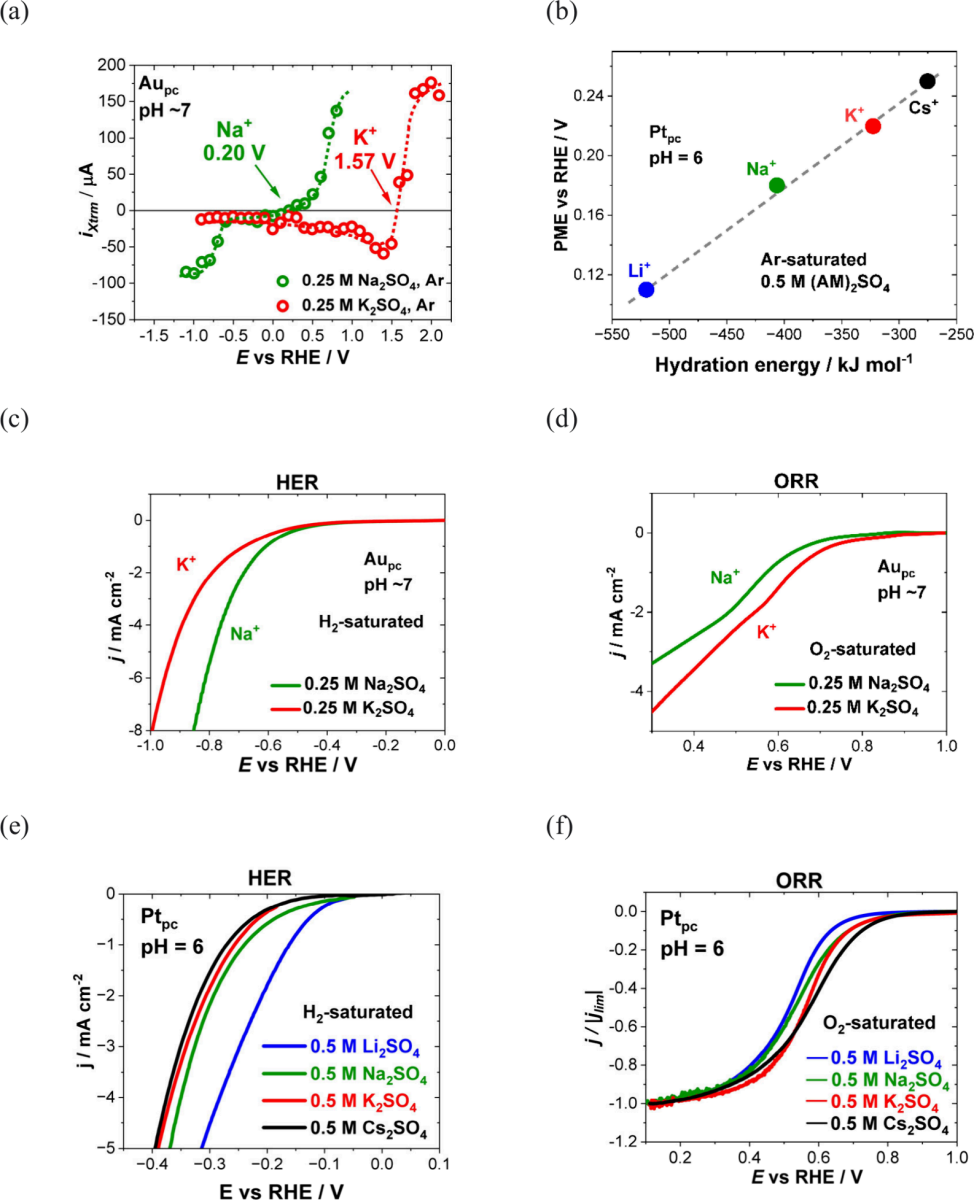
(a) LICT measurements of Au_pc_ in 0.25 M (AM)_2_SO_4_ (AM^+^ = Na^+^, K^+^) at pH ∼7, utilizing Ar-saturated electrolytes. (b) PME as a function of the hydration energies of the cations employed in the 0.5 M (AM)_2_SO_4_ (AM^+^ = Li^+^, Na^+^, K^+^, Cs^+^) electrolyte for Pt_pc_ at pH 6. (c, e) HER and (d, f) ORR activity measurements of Au_pc_ and Pt_pc_ conducted with the previously utilized electrolytes in the LICT measurements, respectively. Adapted from ref (128). CC BY 4.0.

Since no comprehensive theory yet would correlate the PME with the activity of an electrocatalytic reaction, the relationship between the PME and the activity discovered by Briega-Martos et al.^471^ could be a coincidence. However, Ding et al.,^128^ in the same year, reported similar results. They employed the LICT technique to study the influence of alkali metal cations on the PME of the polycrystalline (pc) Pt and Au electrodes measured in 0.5 M Ar-saturated (AM)_2_SO_4_ (AM^+^ = Li^+^, Na^+^, K^+^, Cs^+^) electrolytes for Pt_pc_ at pH 6 and in neutral pH (pH ∼7) solutions of 0.25 M Na_2_SO_4_ and K_2_SO_4_ for Au_pc_. In agreement with Briega-Martos et al., the PME varies with the hydration energy of the alkali metal cation, as displayed in Figure 30, parts a and b for Au_pc_ and Pt_pc_, respectively. In addition, Ding et al. compared the PME trend obtained with the activity results for HER and ORR, conducted under identical conditions with H_2_-saturated and O_2_-saturated electrolytes (Figure 30c–f). These findings further confirm the trend that a PME closer to the thermodynamic equilibrium potential of an electrocatalytic reaction leads to increased reaction rates, as explained in section 2. Specifically, HER rates are increased in Li^+^-containing solutions, whereas the ORR activity is elevated in the presence of Cs^+^. Similar findings were observed for Au_pc_ at pH 7 in sodium and potassium salt solutions, with the lowest PME value detected in Na^+^-containing electrolyte and the highest HER activity compared to K^+^-containing electrolyte.

In summary, the role of the electrolyte appears to be highly complex due to interrelated parameters of the electrolyte, such as the nature of the ions. Alkali metal cations have a significant impact on EDL properties, highlighting the critical role of cation size and hydration energy in influencing reaction intermediates, bond characteristics, and *C*_dl_ at the electrode–electrolyte interfaces. While the literature that established a correlation between *C*_dl_ and electrocatalytic activity is scarce, focusing on this relationship in future research could significantly enhance our understanding of the EDL and determine if the *C*_dl_ can effectively predict electrocatalytic performance. Regarding the PME, a clear correlation between the PME and the electrocatalytic activity has been established depending on the utilized cation. Although this observation is quite promising, our current understanding of the EDL is far from explaining this correlation. Furthermore, it remains unclear whether this correlation with the employed cation characteristics persists when more complex materials are explored, such as metal chalcogenides, ternary compounds, and high entropy alloys.

### 7.2 The Influence of Alkali Metal Cation Concentration

**Figure 31:**
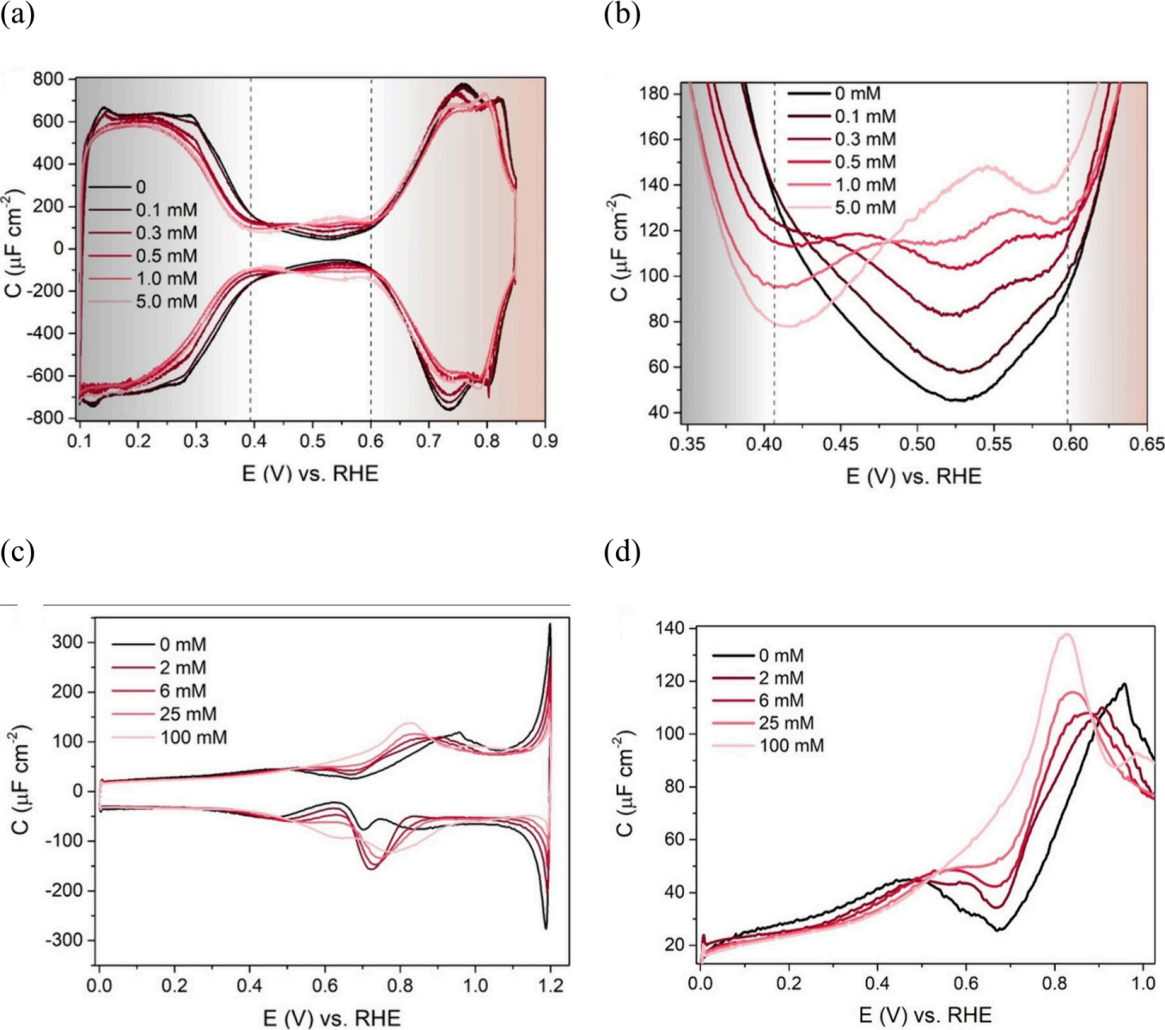
(a) The curves of Gouy–Chapman capacitance and (b) the magnified view for Pt(111) electrode surface in 0.1 mM HClO_4_ + *x* mM NaClO_4_. The capacitance minimum is located at 0.53 V (pzc). (c) The curves of Gouy–Chapman capacitance minimum and (d) magnified view for Au(111) electrode surface in 0.1 mM HClO_4_ + *x* mM NaClO_4_. The capacitance minimum is located at 0.69 V. Reprinted from ref (472). CC BY NC 4.0.

Beyond the influence of specific cations, the concentration of these cations in the electrolyte crucially affects *C*_dl_ properties. This dependency was underscored in a study by Ojha et al.^472^ in 2020, where the authors employed the CV method to determine the Gouy–Chapman capacitance minimum for Pt(111) in a nonspecifically adsorbed electrolyte (perchloric acid) with a low concentration (0.1 mM HClO_4_ + *x* mM NaClO_4_) in pH 4 at the potential of zero charge (pzc = 0.525 V_RHE_). Figure 31a,b illustrates the Gouy–Chapman capacitance minimum for the Pt(111) electrode surface across varying NaClO_4_ concentration. A clear trend emerges indicating that *C*_dl_ increases with increasing electrolyte ionic strength. The observed capacitance minimum aligns with data reported from other groups.^111,116^ Notably, at an ionic strength of 5 mM, the capacitance reaches a maximum, a feature also documented by Lynch et al.^473^ At ionic strengths of 0.5 and 1 mM, the observed pattern reveals a single minimum and two maxima. This behavior can be attributed to the interplay of a pronounced Helmholtz capacitance at a potential deviating from the pzc and a minimum Gouy–Chapman capacitance at the pzc. Additionally, the Gouy–Chapman capacitance minimum at the pzc (0.69 V_RHE_) for the Au(111) electrode surface was examined at more significant electrolyte concentrations (1 mM HClO_4_ + *x* mM NaClO_4_) under pH 3, as depicted in Figure 31c,d. The trend observed with the increasing ionic strength equals that of the Pt(111) electrode. Similar to the ionic strength of 5 mM for Pt(111), the GC capacitance minimum disappears in 100 mM electrolyte for Au(111).

Again, the observation of trends in the *C*_dl_ or pzc with changes in NaClO_4_ concentration raises fundamental questions about how these trends correlate with the activity of an electrochemical reaction. Making predictions about these correlations without specific activity experiments is vague and will not be of any help in improving our fundamental understanding of the interconnection between EDL properties and kinetic parameters.

**Figure 32:**
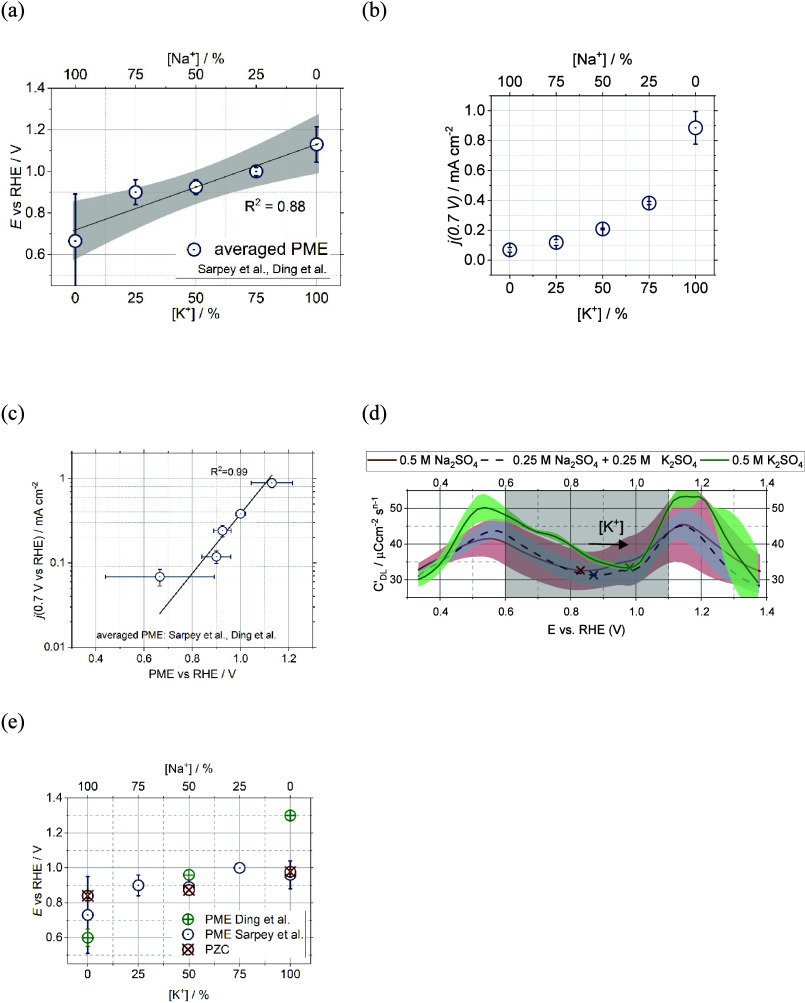
(a) Averaged PME of Au(pc) from Sarpey et al. and Ding et al. depending on the molar ratios of the employed Na_2_SO_4_ and K_2_SO_4_ electrolytes. (b) Specific current densities extracted from ORR polarization curves at 0.7 V vs RHE for varying molar ratios of cations. (c) Dependency of the averaged PME from (a) and the specific current densities from (b). (d) *C*_dl_ as a function of the electrode potential for different concentrations of 0.5 M Na_2_SO_4_, 0.25 M Na_2_SO_4_ + 0.25 M K_2_SO_4_, and 0.5 M K_2_SO_4_. (e) Dependency of the nonaveraged PME values from Sarpey et al. and Ding et al., as well as for the pzc in correlation with the molar ratios of the employed cations. Adapted from ref (474). CC BY 4.0.

An exemplary study that delves into the influence of the cation nature and cation concentration on EDL parameters and the subsequent correlation toward electrocatalytic activity was published by Sarpey et al.^474^ In this study, both the nature and the concentration of the ions were changed by mixing 0.5 M Na_2_SO_4_ and 0.5 M K_2_SO_4_ (pH 8) electrolytes in different ratios. The study is a follow-up to the previously published work by Ding et al.,^127^ who investigated the pH effect and influence of the alkali metal ions on the PME for Au(pc). For this reason, Sarpey et al. averaged their determined PME values at pH 8 with the previous PME results from Ding et al. However, significant differences in the PME values comparing both studies arise, which highlights once more the difficulties in determining EDL properties reproducibly. Nevertheless, for the averaged PME values, a linear trend arises, with the lowest PME obtained at pure Na_2_SO_4_ and the largest at pure K_2_SO_4_ electrolyte, as can be seen in Figure 32a. Again, the understanding of the PME so far implies that a larger ORR activity is reached if the PME lies closer to the equilibrium potential of 1.23 V_RHE_, resulting in an enhanced activity with increasing K^+^ concentration in the performed investigation. ORR activity measurements validated this prediction, highlighted by Figure 32b, displaying the specific current densities determined at 0.7 V_RHE_ versus the electrolyte composition. Figure 32c shows the correlation between this current density and the averaged PME from both studies. EIS measurements were conducted employing an EEC, which consists of the classical R-CPE elements in combination with adsorption capacitance *C*_ad_ and resistance *R*_ad_, which considers specific adsorption. Figure 32d displays *C*_dl_ values determined for three different concentration ratios of Na_2_SO_4_ and K_2_SO_4_ in dependence on the electrode potential. The minimal *C*_dl_ values of these curves are comparable to each other, considering the error bars that were obtained, and therefore do not serve as a suitable descriptor for electrocatalytic activity in this case. Furthermore, the potential corresponding to those minimal *C*_dl_ values provides estimates of the pzc. The trend of the pzc with different concentration ratios is shown in Figure 32e, alongside the nonaveraged PME values, determined by Sarpey et al., and the PME values from the previous study by Ding et al. While the pzc values align reasonably well with the PME determined by Sarpey et al., discrepancies arise compared to the PME detected by Ding et al. Nevertheless, a tendency arises, indicating larger ORR activities for more positive pzc values.

In conclusion, the study shows that both PME and pzc seem to correlate directly with the activity of the electrochemical reaction, while the *C*_dl_ value seems irrelevant in terms of any predictions. One reason that the *C*_dl_ value cannot predict electrocatalytic activity could be attributed to the fact that it describes the EDL structure and characteristics that are present in the double-layer potential region and not in the Faradaic regime at which the electrocatalytic reaction occurs. Nevertheless, the study can be considered noticeable since it aims to establish correlations among all of the three critical EDL parameters, namely PME, pzc and *C*_dl_, with the activity of an electrochemical reaction. However, numerous additional questions remain unsolved. First is whether the observed trends apply to other noble metals or even extend to non-noble metals, metal oxides, ternary compounds, or high entropy alloys. Second, the influence originates from the different crystallographic orientations of the polycrystalline material employed by Sarpey et al. Third, it is important to determine whether the observed correlations also remain established for additional electrochemical reactions, eventually even providing insights into the selectivity of more complex reactions such as the CO_2_RR. Fourth, what are the underlying physical and chemical reasons for the observing correlation? Addressing these questions requires further fundamental research that could help us in developing a more comprehensive understanding of the interplay between EDL properties and electrocatalytic activity.

### 7.3 The Influence of Solution pH

**Figure 33:**
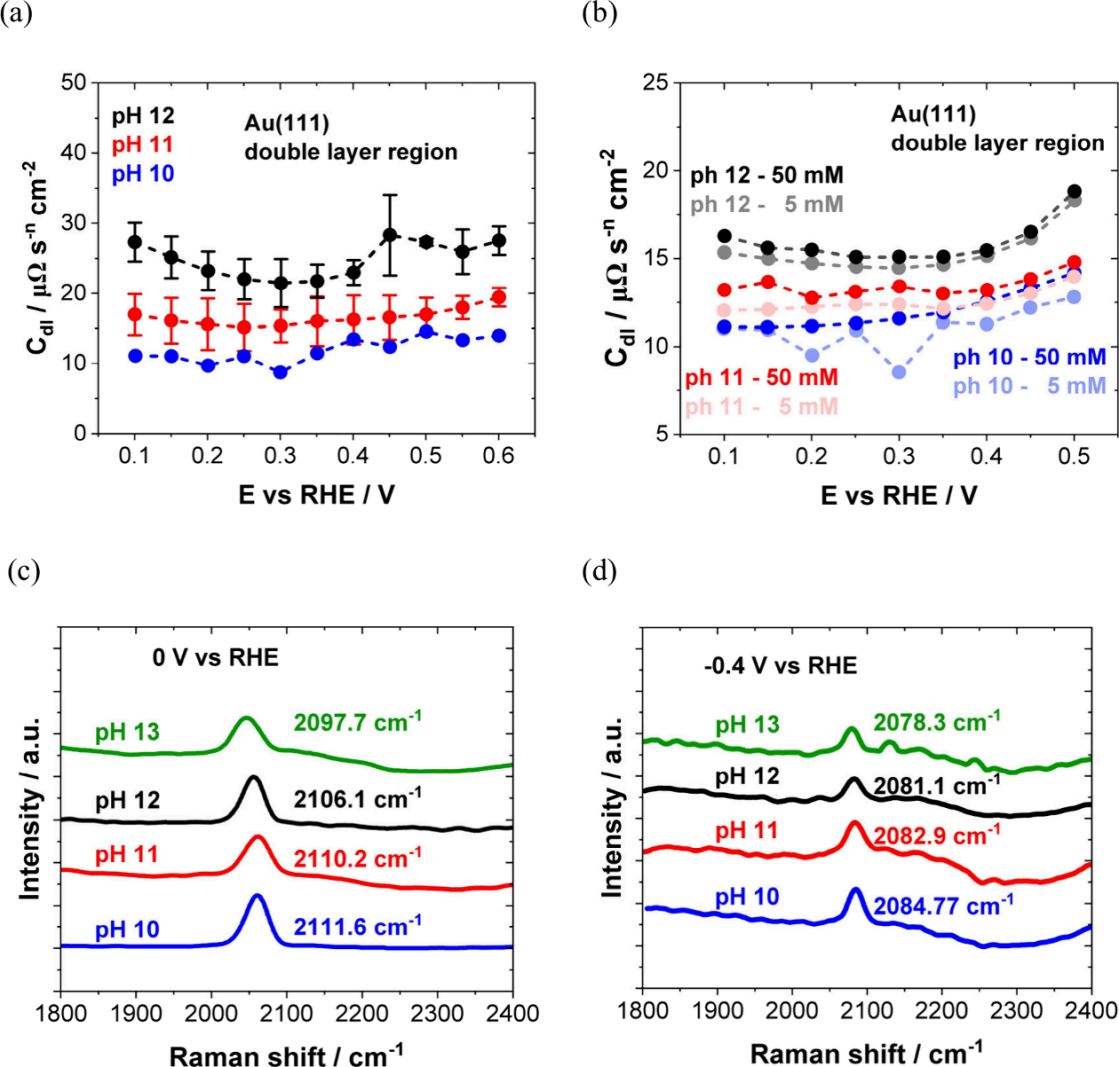
(a) Results of specific *C*_dl_ as a function of potential and pH for Au(111) layer. (b) Comparison of specific *C*_dl_’s with various electrolyte concentrations in the same pH. In situ SERS of hydrogen adsorption on Au with increasing pH from 10 to 13 at (c) 0 and (d) −0.4 V. Adapted from ref (475). CC BY 4.0.

Goyal et al.^475^ illuminated the complexities of deconvoluting the interconnected effects of pH and concentration variations on the interfacial region. Their research examined the consequences of both these factors on the capacitance at the Au(111) electrode–electrolyte interface. As an EEC, they employed a model that includes the adsorption resistance *R*_a_ and capacitance *C*_a_, alongside the conventional R-CPE model accounting for electrolyte resistance and *C*_dl_. The EIS measurements were conducted at potentials slightly negative relative to the *E*_pzc_ of Au(111) (1.064 V_RHE_). Furthermore, they used various concentration combinations of NaOH, NaClO_4_, and HClO_4_ to manipulate the bulk pH and overall ion concentration. The data depicted in Figure 33a reveal that the specific *C*_dl_ increases with increasing pH values, a trend that they ascribe to a rise in cation concentration at the interface, consequent to the increased pH. To validate this postulation, they probed the influence of cation concentration (*C*_Na^+^_ being either 5 or 50 mM in NaClO_4_) while maintaining a constant pH. As depicted in Figure 33b, the *C*_dl_ indeed increases with a rise in cation concentration, underscoring that the parameters of electrolyte pH and bulk cation concentration have similar effects on the interfacial capacitance. To derive deeper insights from the pH dependency, in situ SERS was employed to distinguish shifts in the Au–H vibrational bond across varied pH levels, as illustrated in Figure 33c,d. A decline in wavenumber with increasing pH was noted, aligning with findings from another research group,^196^ suggesting that the bond strength of adsorbed hydrogen during the HER phase also depends on the pH.

**Figure 34:**
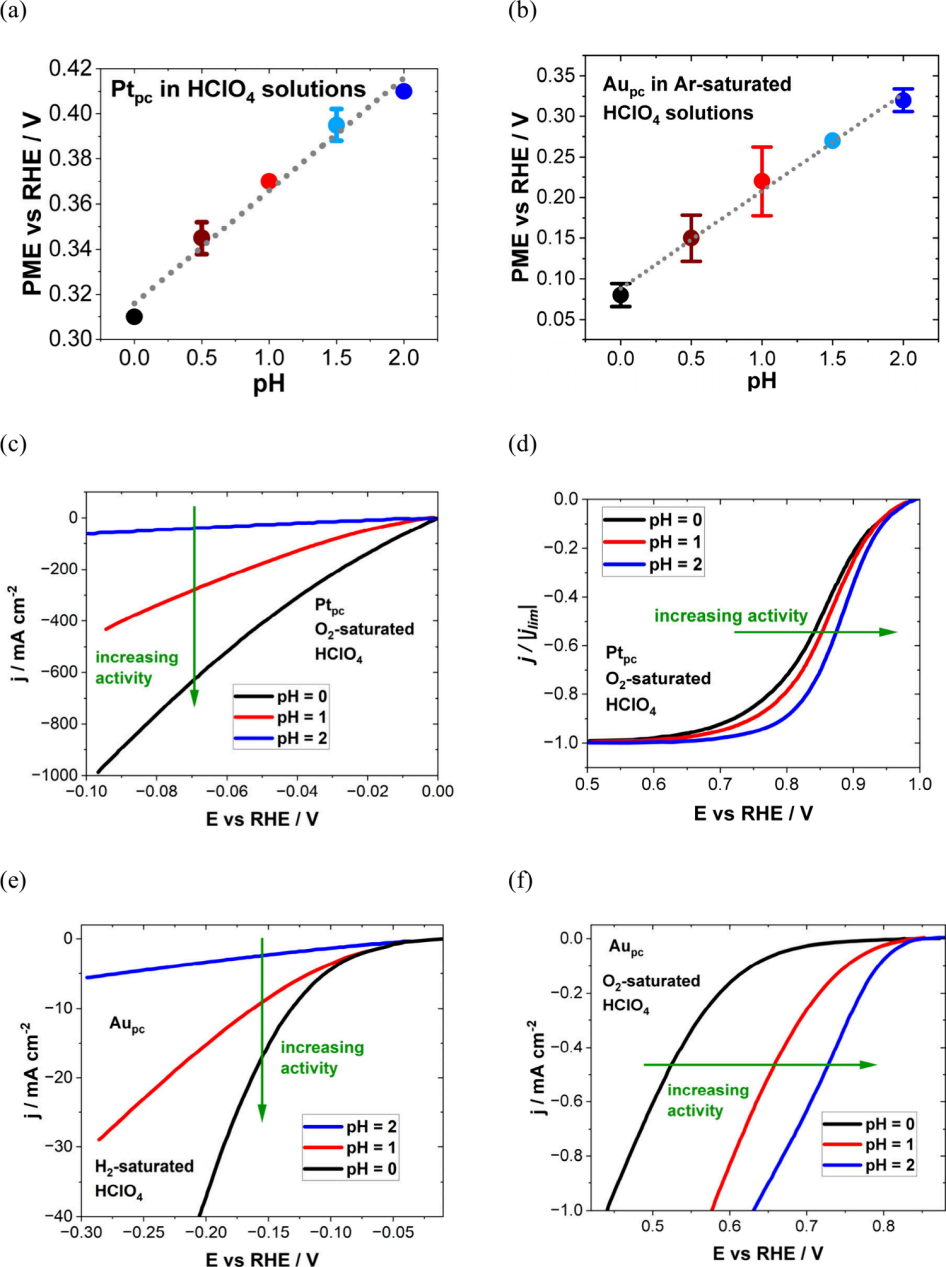
PME as a function of the pH in the case of (a) Pt_pc_ and (b) Au_pc_ for HClO_4_ electrolytes. (c, d) HER and (e, f) ORR activity measurements of Pt_pc_ and Au_pc_ within the same HClO_4_ electrolytes of varying pH. Adapted from ref (128). CC BY 4.0.

Similar correlations of EDL properties with electrocatalytic activity depending on the pH were conducted by Ding et al.,^128^ who explored the influence of pH on the PME for Pt_pc_ and Au_pc_ electrodes using varying HClO_4_ electrolytes of different concentrations, as shown in Figure 34a,b. This study revealed that, for both electrodes, PME values increased at higher pH. The following activity measurements for HER and ORR (Figure 34c–f), carried out under identical conditions, again showed a consistent relationship between PME and the activity of the electrocatalytic reaction, as previously discussed.^128^

**Figure 35:**
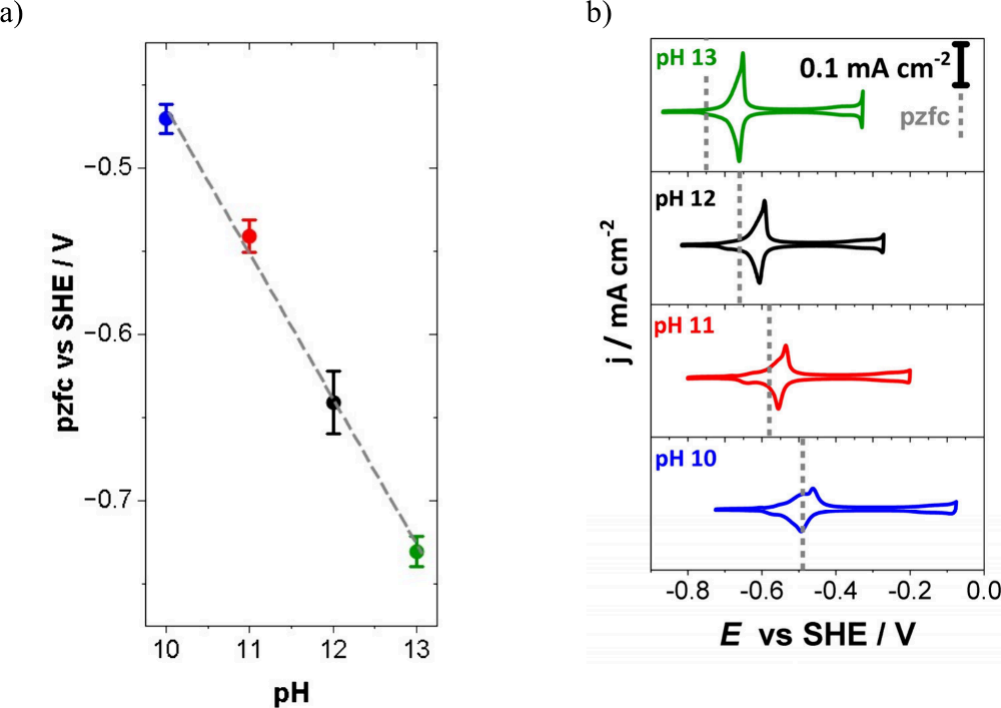
(a) The influence of pH on pzfc. (b) Cu(111) cyclic voltammograms in 0.1 M NaClO_4_ solution at various pH values displaying electrochemical behavior between 0.9 and 0.0 V_SHE_ at a scan rate of 50 mV s^–1^. The pzfc locations are indicated by the vertical lines. Adapted from ref (476). CC BY 4.0.

As described in section 2, the PME is usually estimated to be close to the pzc. As the effects of electrolyte composition and concentration on the PME were discussed above, the dependence of the electrolyte on the pzc is equally noteworthy. In a recent study in 2021 by Auer et al.^476^ the effect of pH on the pzfc was investigated in alkaline media for the Cu(111) electrode. Their findings indicated a linear relationship between the pzfc and the electrolyte pH, with the pzfc decreasing as the pH increased (as depicted in Figure 35a). Furthermore, at reduced pH levels, the pzfc shifted to potentials corresponding to the OH-adsorption region (Figure 35b). Complementing these electrochemical measurements in situ electrochemical scanning tunneling microscopy was utilized to examine the interfacial structure. At the pzfc, the Cu(111) surface exhibited initial signs of structural reconstruction at both pH 13 and pH 11, while at higher potentials full surface reconstruction was observed.

In summary, the complexity of the interface increases further due to the interconnection of the pH and the ionic concentration, especially since several studies indicate that the ionic concentration increases drastically at the solid–liquid interface compared to the bulk. Therefore, additional fundamental studies need to be performed in the future to deconvolute the pH and ionic concentration at the interface. We highlight that such investigations are essential to acquire a more fundamental and deeper understanding of how pH and ionic concentration individually alter EDL properties. As discussed in the sections above, it seems like EDL properties are directly related to the activity of electrochemical reactions, especially in the case of the PME. Similar to other EDL parameters, the presented work displays clear trends in how the PME is affected by the electrolyte. The relative position of the PME related to the equilibrium potential of an electrochemical reaction emerges as a parameter to quantify the activity. The smaller this parameter, the larger the activity of the electrochemical reaction. This trend has been reported by various groups, making it unlikely to be a coincidence. However, a fundamental theoretical framework to explain this correlation is still under current investigation. For its development, further research is mandatory, particularly involving studies with single crystals. Those model systems and the resulting data will provide essential insights for theoreticians to improve the theoretical models. A summary of selected EDL parameters discussed within this study is provided in Tables 5 and 6.

**Table 5:** Selected Representative Studies Exploring Variations in *C*_dl_ Values Based on the Employed Electrolyte^a^

electrode	*C*_dl_	potentials	methods	electrolyte	ref
HOPG	(4.1 ± 0.3) μF cm^–2^	0 V vs Ag/AgCl	EIS	0.5 M HCl	(466)
HOPG	(4.7 ± 0.4) μF cm^–2^	0 V vs Ag/AgCl	EIS	0.5 M LiCl	(466)
HOPG	(5.6 ± 0.4) μF cm^–2^	0 V vs Ag/AgCl	EIS	0.5 M NaCl	(466)
HOPG	(8.1 ± 0.2) μF cm^–2^	0 V vs Ag/AgCl	EIS	0.5 M KCl	(466)
HOPG	(8.9 ± 0.3) μF cm^–2^	0 V vs Ag/AgCl	EIS	0.5 M RbCl	(466)
HOPG	(9.4 ± 0.6) μF cm^–2^	0 V vs Ag/AgCl	EIS	0.5 M CsCl	(466)
Pt(111)	(26 ± 2) μF cm^–2^	pzc	EIS	0.05 M LiClO_4_	(393)
Pt(111)	(30 ± 0.3) μF cm^–2^	pzc	EIS	0.05 M NaClO_4_	(393)
Pt(111)	(36 ± 0.4) μF cm^–2^	pzc	EIS	0.05 M KClO_4_	(393)
Pt(111)	(39 ± 10) μF cm^–2^	pzc	EIS	0.05 M RbClO_4_	(393)
Pt(111)	(40 ± 7) μF cm^–2^	pzc	EIS	0.05 M CsClO_4_	(393)
Au(111)	14 μF cm^–2^	pzc	EIS	0.05 M LiClO_4_	(393)
Au(111)	(21 ± 4) μF cm^–2^	pzc	EIS	0.05 M NaClO_4_	(393)
Au(111)	(26 ± 3) μF cm^–2^	pzc	EIS	0.05 M KClO_4_	(393)
Au(111)	(27 ± 5) μF cm^–2^	pzc	EIS	0.05 M RbClO_4_	(393)
Au(111)	(31 ± 6) μF cm^–2^	pzc	EIS	0.05 M CsClO_4_	(393)
Pt(12 10 5)	(4 ± 1) μF cm^–2^	pzc	EIS	0.05 M LiClO_4_	(393)
Pt(12 10 5)	(7 ± 1) μF cm^–2^	pzc	EIS	0.05 M NaClO_4_	(393)
Pt(12 10 5)	(10 ± 5) μF cm^–2^	pzc	EIS	0.05 M KClO_4_	(393)
Pt(12 10 5)	(14 ± 7) μF cm^–2^	pzc	EIS	0.05 M RbClO_4_	(393)
Pt(12 10 5)	(19 ± 13) μF cm^–2^	pzc	EIS	0.05 M CsClO_4_	(393)
Pt(775)	(12 ± 1) μF cm^–2^	pzc	EIS	0.05 M LiClO_4_	(393)
Pt(775)	(16 ± 1) μF cm^–2^	pzc	EIS	0.05 M NaClO_4_	(393)
Pt(775)	(19 ± 1) μF cm^–2^	pzc	EIS	0.05 M KClO_4_	(393)
Pt(775)	(24 ± 5) μF cm^–2^	pzc	EIS	0.05 M RbClO_4_	(393)
Pt(775)	(26 ± 3) μF cm^–2^	pzc	EIS	0.05 M CsClO_4_	(393)
NiOOH	(216 ± 66) μF cm^–2^	1.6 V vs RHE	EIS	LiOH, pH 13	(468)
NiOOH	(291 ± 36) μF cm^–2^	1.6 V vs RHE	EIS	NaOH, pH 13	(468)
NiOOH	(278 ± 15) μF cm^–2^	1.6 V vs RHE	EIS	KOH, pH 13	(468)
NiOOH	(289 ± 12) μF cm^–2^	1.6 V vs RHE	EIS	CsOH, pH 13	(468)
Au(pc)	(32.5 ± 5.5) μF cm^–2^	pzc	EIS	0.5 M Na_2_SO_4_, pH 8	(474)
Au(pc)	(31.3 ± 3) μF cm^–2^	pzc	EIS		(474)
Au(pc)	(33.4 ± 0.5) μF cm^–2^	pzc	EIS	0.5 M K_2_SO_4_, pH 8	(474)

a The table columns list the electrode composition/structure, corresponding *C*_dl_ values, potentials at which *C*_dl_ values were determined, methods used, electrolytes employed, and references cited. It should be noted that several *C*_dl_ values are estimated from the original published data. This table serves as a comprehensive overview of the cited literature.

**Table 6:** Selected Representative Studies Exploring Variations in PME/pzc/pzfc Values Based on the Employed Electrolyte^a^

electrode	potential	parameter	method	electrolyte	ref
Au(pc)	0.2 V vs RHE	PME	LICT	0.25 M Na_2_SO_4_, pH 7	(128)
Au(pc)	1.57 V vs RHE	PME	LICT	0.25 M K_2_SO_4_, pH 7	(128)
Pt(pc)	0.11 V vs RHE	PME	LICT	0.5 M Li_2_SO_4_, pH 6	(128)
Pt(pc)	0.18 V vs RHE	PME	LICT	0.5 M Na_2_SO_4_, pH 6	(128)
Pt(pc)	0.22 V vs RHE	PME	LICT	0.5 M K_2_SO_4_, pH 6	(128)
Pt(pc)	0.25 V vs RHE	PME	LICT	0.5 M Cs_2_SO_4_, pH 6	(128)
Au(pc)	(0.73 ± 0.22) V vs RHE	PME	LICT	0.5 M Na_2_SO_4_, pH 8	(474)
Au(pc)	(0.9 ± 0.06) V vs RHE	PME	LICT		(474)
Au(pc)	(0.89 ± 0.02) V vs RHE	PME	LICT		(474)
Au(pc)	(1 ± 0.02) V vs RHE	PME	LICT		(474)
Au(pc)	(0.96 ± 0.08) V vs RHE	PME	LICT	0.5 M K_2_SO_4_, pH 8	(474)
Au(pc)	(0.6 ± 0.05) V vs RHE	PME	LICT	0.5 M Na_2_SO_4_, pH 8	(127)
Au(pc)	(0.96 ± 0.03) V vs RHE	PME	LICT		(127)
Au(pc)	(1.3 ± 0.03) V vs RHE	PME	LICT	0.5 M K_2_SO_4_, pH 8	(127)
Au(pc)	(0.67 ± 0.23) V vs RHE	PME	LICT	0.5 M Na_2_SO_4_, pH 8	(127, 474)
Au(pc)	(0.9 ± 0.06) V vs RHE	PME	LICT		(127, 474)
Au(pc)	(0.93 ± 0.04) V vs RHE	PME	LICT		(127, 474)
Au(pc)	(1 ± 0.02) V vs RHE	PME	LICT		(127, 474)
Au(pc)	(1.13 ± 0.09) V vs RHE	PME	LICT	0.5 M K_2_SO_4_, pH 8	(127, 474)
Au(pc)	(0.84 ± 0.01) V vs RHE	pzc	EIS	0.5 M Na_2_SO_4_, pH 8	(474)
Au(pc)	(0.87 ± 0.03) V vs RHE	pzc	EIS		(474)
Au(pc)	(0.98 ± 0.01) V vs RHE	pzc	EIS	0.5 M K_2_SO_4_, pH 8	(474)
Pt(pc)	0.31 V vs RHE	PME	LICT	HClO_4_, pH 0	(128)
Pt(pc)	(0.35 ± 0.01) V vs RHE	PME	LICT	HClO_4_, pH 0.5	(128)
Pt(pc)	0.37 V vs RHE	PME	LICT	HClO_4_, pH 1	(128)
Pt(pc)	(0.395 ± 0.01) V vs RHE	PME	LICT	HClO_4_, pH 1.5	(128)
Pt(pc)	0.41 V vs RHE	PME	LICT	HClO_4_, pH 2	(128)
Au(pc)	(0.08 ± 0.01) V vs RHE	PME	LICT	HClO_4_, pH 0	(128)
Au(pc)	(0.15 ± 0.03) V vs RHE	PME	LICT	HClO_4_, pH 0.5	(128)
Au(pc)	(0.22 ± 0.04) V vs RHE	PME	LICT	HClO_4_, pH 1	(128)
Au(pc)	0.27 V vs RHE	PME	LICT	HClO_4_, pH 1.5	(128)
Au(pc)	(0.32 ± 0.01) V vs RHE	PME	LICT	HClO_4_, pH 2	(128)
Cu(111)	(−0.47 ± 0.01) V vs SHE	pzfc	LICT	0.1 M NaClO_4_, pH 10	(476)
Cu(111)	(−0.54 ± 0.01) V vs SHE	pzfc	LICT	0.1 M NaClO_4_, pH 11	(476)
Cu(111)	(−0.64 ± 0.02) V vs SHE	pzfc	LICT	0.1 M NaClO_4_, pH 12	(476)
Cu(111)	(−0.73 ± 0.01) V vs SHE	pzfc	LICT	0.1 M NaClO_4_, pH 13	(476)

a The table columns list the electrode composition/structure, potential at which the respective parameters were determined, which parameter was explored (PME/pzc/pzfc), methods used, electrolytes employed, and references cited. It should be noted that several PME/pzc/pzfc values are estimated from the original published data. This table serves as a comprehensive overview of the cited literature.

### 7.4 DFT Simulations

A series of first-principles studies has focused on the role of electrolyte composition, including solution pH, in the basic properties of EDL.^84,92,477−481^ For example, in a recent DFT study, the effect of solution pH was examined for the Pt(111), Au(111), and Au(100) electrodes in the context of the four-electron ORR.^478^ By using the established CHE approach in combination with a microkinetic model, it was found that Au(111) and Au(100), for which the formation of *OOH is the rate-limiting step, should be more influenced by pH because of the strong electric field effects on the *OOH adsorbate. A much weaker field effect was detected on Pt(111), for which the ORR is limited by the removal of *OH from the surface. It was suggested that this might be a common phenomenon in all weak-binding ORR catalysts.

DFT finite electric field MD simulations were undertaken to explore the pH dependence of the Helmholtz capacitance at an electrified TiO_2_(110)/NaCl electrolyte interface.^480^ The asymmetric pH dependence with a higher Helmholtz capacitance at higher pH was observed in agreement with titration experiments for TiO_2_ and some other metal oxides such as ZnO. Microscopically, this was explained by the observation of stronger structural fluctuations of water molecules, i.e., a much wider distribution of water dipole moments at high pH (negatively charged surface) leading to a much larger capacitance. At low pH, however, proton transfer increases the capacitance value by reducing the charge separation distance in the EDL. This shows that the surface acidity of the metal oxide is another determining factor for the Helmholtz capacitance.

**Figure 36:**
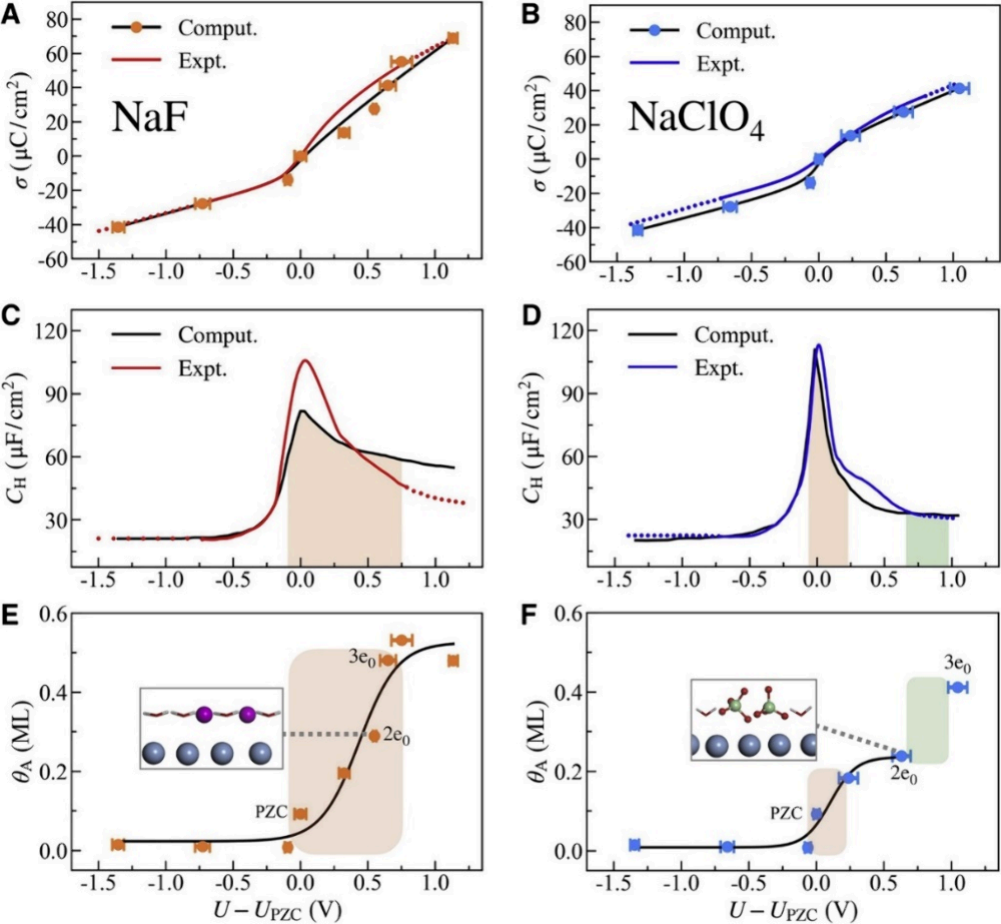
Plots of (A, B) surface charge densities (σ), (C, D) Helmholtz capacitance (*C*_H_), and (E, F) coverages of chemisorbed water (θ_A_) as a function of electrode potential (*U*) at the Ag(111)/water interfaces in NaF (A, C, and E) and NaClO_4_ (B, D, and F) aqueous solutions. The orange and blue dots with error bars were calculated with AIMD simulations, and the black curves are their corresponding trend lines. The red and blue curves are experimental data and used for comparison. The two insets show the representative snapshots of Ag(111)/water interfaces at surface charge 2*e*_0_ in the NaF and NaClO_4_ solutions. Ag, Cl, F, O, and H atoms are colored gray, green, purple, red, and white, respectively. Ag(111) and ions are highlighted with ball models, and water molecules are shown with stick models. Adapted from ref (482). CC BY NC-ND 4.0.

Many recent DFT investigations have focused on the role of electrolyte ions in the EDL properties and electrocatalytic processes.^22,482^ For example, in a recent AIMD investigation, the role of solvated ions within the EDL in the capacitive behavior of electrified Ag(111)/water interfaces was addressed.^482^ One important experimental feature prompting an important role of electrolyte anions is the observation that the capacitance curve is narrower for the NaClO_4_ than for the NaF solution on the positive potential side of the pzc. Figure 36 shows the comparison between simulated and experimental data for the Ag(111)/water system in NaF and NaClO_4_ solutions. Overall, simulations reveal that, unlike small F^–^ ions, the larger ClO_4_^–^ anions lead to an increased width of the compact Helmholtz layer and thereby reduce the water content in the EDL.

In general, there is a clear understanding that electrolyte ions can substantially modify the properties of interfacial water in the EDL, having pronounced effects on the EDL capacitance. However, it remains highly challenging to simulate explicit electrode/electrolyte interfaces in the presence of solvated ions under potential control and connect modeling results with experimental observations.

## 8 Adsorbates/Reaction Intermediates and Their Effect on Catalytic Activity

Extensive research is being conducted about interfacial water within the solid–liquid interface, as well as about electrolyte effects on the EDL, as elucidated in the previous sections. However, the interactions between interfacial water, electrolyte species, and reaction intermediates adsorbed to the electrode surface were examined to a lesser extent. Also, studies of the EDL and its properties are hardly linked to electrocatalytic reactions. This might appear surprising at first as the EDL is where electrocatalytic processes take place, surrounded by electrolyte species and water. However, the complexity of these systems requires the application of techniques and experimental setups that can lead to the detection, tracking, and differentiation of reaction intermediates interfacial water and electrolyte species during reaction. In the following, we will guide the reader through selected research works, starting from early studies on adsorbed species in the EDL to state-of-the-art experimental techniques, which strive to link observed EDL phenomena to electrocatalytic reaction parameters with the help of theoretical calculations and modeling.

Many studies in the past investigated adsorption phenomena of various anions and cations on model electrode surfaces, such as single crystals, to obtain detailed data about adsorption processes, EDL structures and changes, and the dynamic properties of surface adsorbates. Bromine adsorption on Ag(100)^51,483^ and Ag(001)^484^ single-crystal surfaces has been studied extensively early on, leading to findings such as a 4-fold hollow adsorption site of bromine with partially covalent bonding, as well as the formation of an ordered *c*(2 × 2) overlayer via SXS and in situ XAFS. This was also confirmed from a theoretical point by using dynamic Monte Carlo simulations of a lattice-gas model for the adsorption of bromine on Ag(100).^485^ Furthermore, the role of cations present in the electrolyte has been studied. For example, the structure of Cs^+^ cations in the EDL near adsorbed bromine species on Ag(100) is dependent on the coverage of the bromine adlayer, i.e., the applied electrode potential.^47−49^ Cs^+^ cations are located in the hollow sites of the bromine adlayer in the OHP via noncovalent interactions, separated by a single water layer from the adsorbed bromine in the IHP, as observed via in situ SXD and TRXD. Differences between the adsorption of bromine and chlorine species on Ag(100) single-crystal surfaces were shown by fitting adsorption isotherms obtained via equilibrium Monte Carlo simulations to experimental data.^486^ It was revealed that both adsorption processes are dependent on the applied electrode potential, but chlorine adsorption on Ag(100) is not coverage-independent in contrast to bromine, which further demonstrates the complexity of electrochemical systems.

**Figure 37:**
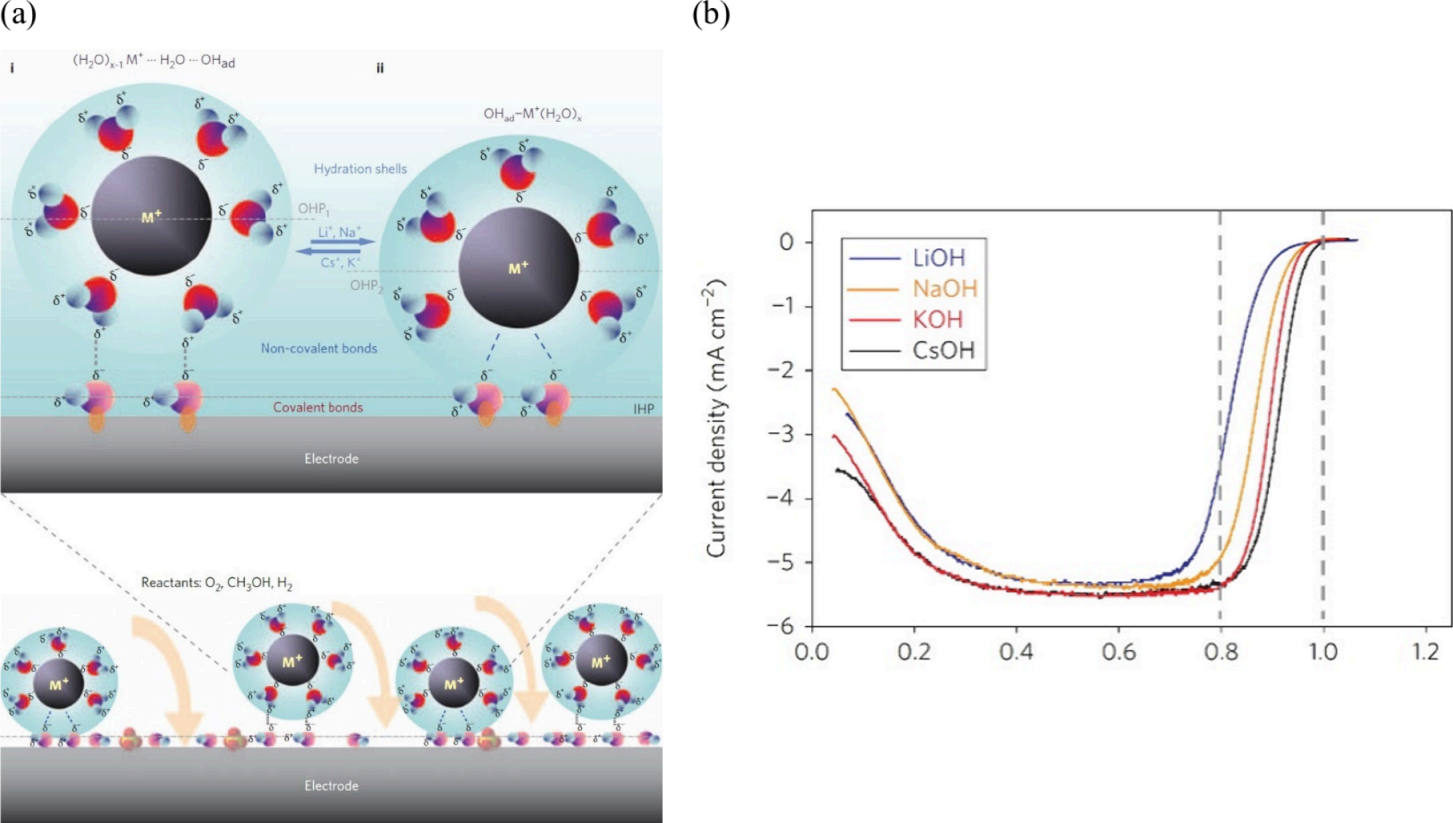
(a) Proposed models for noncovalent interactions between hydrated alkali metal cations and adsorbed OH in the EDL: (i) (H_2_O)_*x*−1_M^+^···H_2_O···OH_ad_ clusters and (ii) OH_ad_···M^+^(H_2_O)_*x*_ clusters. For simplicity, only the first hydration shell is shown in each model. OHP_1_ and OHP_2_ refer to the positions of fully and partially hydrated cations at the outer Helmholtz plane, respectively. The double layer structure with quasi-adsorbed clusters and available platinum sites for adsorption of the reactant species is shown (bottom). (b) ORR polarization curves in the presence of different cations. The vertical dashed lines at 0.8 and 1.0 V indicate regions with similar surface coverages of OH_ad_ where the ORR activity is controlled by the nature of cations. Reprinted with permission from ref (463). Copyright 2009 Springer Nature.

Experimental and theoretical investigations of numerous adsorbates with respect to diffusion dynamics, adsorption sites and structure, and influence on the EDL have also been carried on to other model systems in electrocatalysis, like Pt,^463,487,488^ Au,^36,489−491^ and Cu^171,492−501^ single-crystal surfaces. For example, Simeone et al. presented for the first time a detailed model of the EDL for the Au(111)|H_2_SO_4_ interface by combining distance tunneling spectroscopy and DFT calculations.^36^ They illustrated an absolute distance scale of the adsorption of sulfate ions to the Au(111) surface and showed the interaction between sulfate and coadsorbed hydronium ions from the electrolyte while ruling out specific adsorption of water and bisulfate species. This describes an important step toward a complete understanding of the EDL structure and species arrangement, essential for the design of new materials or electrochemical applications. Using in situ SXRD, Keller et al. investigated the EDL of another well-known electrocatalytic system, Cu(100) covered with chemisorbed Cl^–^ anions, and provided new insights by assessing the lateral ordering of interfacial water and coadsorption of hydronium cations.^171^ Here, interfacial water and hydronium cations arrange in the OHL, attracted by the chemisorbed Cl^–^ anions in the IHL. The significant difference from previous studies of the Cl^–^|water|K^+^ system is that the interfacial water in the Cl^–^|water|hydronium bilayer is shared between the cations in the OHL and the anions in the IHL, while the interfacial water in the Cl^–^|water|K^+^ system was assigned only to the cation solvation shell. This led to a new understanding of the EDL of bilayer systems and the resulting interfacial water and ion dynamics at the interface. Strmcnik et al.^463^ suggested an inverse proportionality between noncovalent interactions between hydrated alkali metal cations M^+^(H_2_O)_*x*_ and adsorbed hydroxides OH_ad_ and electrocatalytic activities of the ORR, HOR, and oxidation of methanol on different Pt electrodes (Figure 37). Unlike conventional models, focusing on covalent adsorbate–electrode surface and long-range electrostatic electrode–electrolyte interactions, they introduced the concept of noncovalent interactions between electrolyte cations and the OH_ad_ reaction intermediate using a combination of experimental and theoretical techniques. The formation of OH_ad_–M^+^(H_2_O)_*x*_ or (H_2_O)_*x*−1_M^+^···H_2_O···OH_ad_ clusters via noncovalent bonding in the EDL when the electrode is polarized is schematically depicted in Figure 37a. Interestingly, the cluster concentration increases with the increasing hydration energy of the alkali metal cations, Li^+^ > Na^+^ > K^+^ > Cs^+^, which is inverse to the observed activity trend of the HOR, ORR, and oxidation of methanol for Pt(111), Pt(pc), and Pt nanoparticles, as shown in Figure 37b. Consequently, the authors proposed that the formed clusters in the EDL are responsible for the movement and transport of reactants in dependence on the interaction strength between OH_ad_ and hydrated cation species. Their results suggest that the interplay of electrode surface, hydrated cation species in the electrolyte, and reaction intermediates is much more complex than described by classic models, which is why sophisticated studies of the EDL in reaction environments are crucial for acquiring a complete view of the complex phenomena at the electrode−electrolyte interface

**Figure 38:**
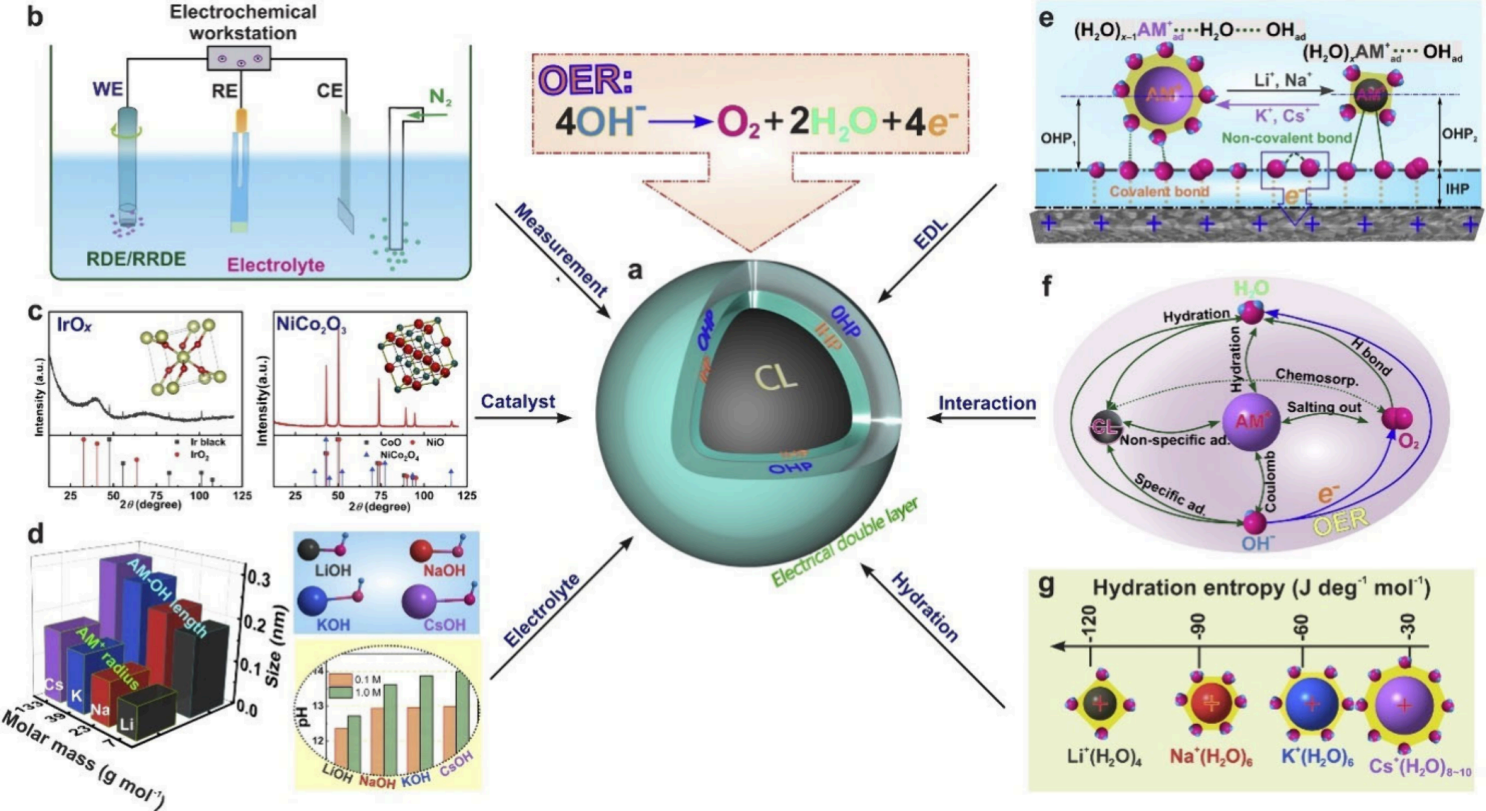
A research overview. (a) Simplified illustration of the EDL in alkaline media. (b) Electrochemical evaluation in a TF-RDE/RRDE configuration. (c) XRD patterns of the IrO_*x*_ and NiCo_2_O_3_ catalysts. (d) Properties of the studied AM–OH electrolytes. Left: AM^+^ radius and AM–OH bond length as a function of AM molar mass. 3D structure of AM–OH (upper right) and pH values of AM–OH (lower right) electrolyte solutions with a concentration of 0.1 or 1 M. (e) Schematic illustration of the EDL with the proposed interaction between OH_ad_ and AM^+^_ad_; OHP_1_ and OHP_2_ refer to the positions of fully and partially hydrated cations at the outer Helmholtz plane, respectively. (f) Possible interactions between interfacial species during OER. (g) Hydration entropies of various AM^+^ cations at 25 °C. Reprinted with permission from ref (504). Copyright 2021 Elsevier.

Furthermore, efforts were made to transfer these experimental and theoretical techniques to other systems, such as semiconductors,^280^ metal oxides,^502−506^ or other materials,^507,508^ to identify adsorption properties, arrangements of species in the EDL, or adsorbate distributions. For example, following the study of Strmcnik et al., del Rosario et al. analyzed the influence of alkali metal cations from the electrolyte solution on the OH_ad_ reaction intermediate during OER and established a comprehensive picture of the processes and interactions taking place in the EDL for IrO_*x*_ and NiCo_2_O_3_ (Figure 38).^504^ Combined XRD and RRDE experiments suggest that the OH_ad_ reaction intermediate during OER forms a (H_2_O)_*x*_AM^+^_ad_···OH_ad_ cluster for small AM cations and a (H_2_O)_*x*−1_AM^+^_ad_···H_2_O···OH_ad_ cluster for the large AM cations K^+^ and Cs^+^, where *x* refers to the hydration number. According to the authors, the latter keeps the adsorbed OH more accessible for OER due to the smaller interaction of the large K^+^ and Cs^+^cations with OH_ad_ due to higher ionization in contrast to the smaller Li^+^ and Na^+^ cations. In addition, the binding interaction with oxygen becomes weaker with increasing cation size, resulting in enhanced direct O_ad_–O_ad_ bonding in the EDL during OER, which is the more favorable OER mechanism compared to forming the OOH_ad_ reaction intermediate. As a result, del Rosario et al. argue that the noncovalent interactions between AM^+^ and OH_ad_ alter the OER mechanism and, therefore, the formation of intermediates and reaction kinetics, similarly as suggested by by Strmcnik et al.^463^ In fact, IrO_*x*_ and NiCo_2_O_3_ showed 4 and 8 times changes in OER activity depending on the AM^+^ cation, respectively, which demonstrates the importance of investigating and understanding the EDL in the presence of adsorbates and electrolyte species. Interestingly, the key findings from this EDL study were applied to synthesize an Ir–Co oxide catalyst in the presence of a K^+^-containing salt, which showed remarkable OER activity, proving that new insights into the EDL structure and processes are essential to boost the field of electrocatalysis.

Unfortunately, systems like semiconductors or metal oxides often demonstrate unexpected complexities with respect to the EDL due to their manifold structure, making it challenging to interpret the experimental findings unambiguously. However, as experimental and theoretical techniques continuously advance and knowledge about solid–liquid interfaces grows, EDL studies of these systems slowly attract scientific research interest. Nevertheless, most research in the field of electrocatalysis focuses on well-defined model systems, which we will focus on in the following sections by highlighting noteworthy recent publications for various electrocatalytic reactions and materials.

As a continuation of early adsorption studies on Ag and Cu, Rahn et al. analyzed the surface dynamics of sulfide adsorbates in the presence of bromine or chlorine adlayers on Ag(100) and Cu(100) using video-STM.^39,40^ They found an interesting phenomenon where the potential-dependent sulfide diffusion on Cu(100) becomes inverted upon changing from bromine to a chlorine adlayer. In contrast, no such radical change could be observed on Ag(100). The inversion of this potential dependence requires a revision of the current understanding of the traditional hopping mechanism of adsorbates. Furthermore, it suggests that simply the nature of a coadsorbed species in the EDL can affect the surface diffusion dynamics of an electrochemical system. DFT calculations suggest an exchange diffusion on the bromine-covered Cu surface, which may be linked to vacancies in the Cu(100) surface underneath the bromine adlayer. Moreover, they observed a variation in the adsorption sites of sulfide on bromine-covered Ag(100) via video-STM.^41^ Besides the classic hopping mechanism of sulfide diffusion, a transition of sulfide adsorbates to subsurface vacancies underneath the bromine adlayer was found, with a similar transport rate compared to the conventional mechanism. While this observation could hold great potential for a new understanding of interfacial dynamics and improvement of electrocatalytic systems, it has yet to be shown whether this is unique to sulfide adsorption on bromine-covered Ag(100) or a universal phenomenon.

In the last years, electrochemical CO_2_ reduction gathered great interest as an attractive strategy for sustainable energy storage and production of chemicals and fuels while reducing CO_2_ emissions. In order to get a fundamental understanding of the processes at the electrode–electrolyte interface, Huang-fu et al. probed the reaction intermediates during CO_2_R on Cu and Au via in situ electrochemical SFG.^509^ While the authors observed linearly adsorbed CO, a key reaction intermediate for CO_2_R, on Au, no such signal could be detected for Cu, suggesting an ordered structure of adsorbed CO on Au. By investigating C–H stretching, the authors detected two SFG bands on Cu, assigned to an adsorbed ethoxy group, a reaction intermediate in the ethanol pathway of CO_2_R. The authors concluded that CO adsorbs in a disordered manner on Cu, leading to the formation of C–C bonds and, therefore, ethoxy in the ethanol pathway, while the ordered adsorption on Au hinders the conversion of C2 products. Wallentine et al. further progressed in the investigation of the Au/electrolyte interface during CO_2_R by using in situ, plasmon-enhanced VSFG with an improved detection limit, circumventing mass transport limitations.^510,511^ These investigations provided new insights into the interfacial processes on Au by measuring the potential-dependent coverage of adsorbed CO. The observed saturation of the electric field in the Stern layer of the EDL suggests the formation of a dense cation layer at the electrode surface, blocking active sites for CO_2_ adsorption. Therefore, the authors provided evidence for a potential-dependent structure of the EDL, thus stating that the EDL structure affects the onset of the CO_2_R activity. The importance of the EDL for CO_2_R was further promoted by Ringe et al., who developed a multiscale approach by linking a microkinetic model to a continuum model containing a detailed description of the EDL structure, buffer equilibria, diffusion, and migration.^512^ This model is in good agreement with experimental data from polarization curves and ATR-SEIRAS measurements on Au.^513^ As a result, the authors were able to determine the conversion of adsorbed COOH to CO at low overpotentials as the rate-determining step, the adsorption of CO_2_ at most relevant overpotentials, and CO_2_ diffusion at high overpotentials. Interestingly, they claim that the electrostatic interaction between the CO_2_ dipole and the interfacial field of the EDL is responsible for the Tafel slope, stating that the EDL structure strongly influences the reaction kinetics by stabilizing the CO_2_ adsorption. Such results offer new implications for the rational design of electrocatalytic systems by tuning the surface charging of the EDL. However, more independent works are needed to compare different results and obtain their validation.

Another study emphasizing the impact of the EDL was recently reported by Rebstock et al., who studied the structure sensitivity of CO_2_R on Au via VSFG by distinguishing between CO adsorption at inactive surface sites and CO generation at active sites.^514^ They observed unchanged bulk solvation shells of cations at inactive sites but a modified cation-dependent solvation structure at undercoordinated active sites, which seemed to be reduced to a solvation shell of a single water layer. Even though these active sites only accounted for 4% of the total surface, the authors suggested that the altered EDL structure causes an ∼25-fold increase in CO_2_R activity due to the modified solvation of the cations at active sites. The authors provided various possible explanations for the observed activity increase, such as the enhanced coordination between cations and reaction intermediates due to the reduced solvation shell or increased electric field between the Au surface and the OHP due to the compressed Stern layer. As a result, they point out the importance of site-dependent solvation of double-layer species to modify the activity of electrocatalytic systems. Deng et al. indicated in a recent study that direct probing of interfacial species via VSFG could also gain information about the selectivity of electrochemical reactions, such as competing CO_2_R and HER on Au electrodes.^515^ They associated the vibrational OH^–^ peak in Ar-purged Na_2_SO_4_, which disappears in a CO_2_-saturated environment, and the CO_3_^2–^ peak in CO_2_-purged Na_2_SO_4_ to water reduction (OH^–^ product) and HCO_3_^–^ reduction (CO_3_^2–^ product), respectively. By monitoring the relative intensities of these vibrational peaks in NaHCO_3_ electrolyte, the authors claimed to be able to differentiate between HER (dominant at high overpotentials) and CO_2_R (dominant at low overpotentials), demonstrating the power of spectroscopic analysis of interfacial species in the EDL.

Another innovative route for enhancing the activity of O_2_, CO_2_, and N_2_ reduction reactions was proposed by Wang et al.,^267^ who investigated the dependence of the ORR on proton activity (or p*K*_a_) by using various ionic-liquid-modified Pt/C and Au/C catalysts. These ionic liquids contained different protic cations, leading to p*K*_a_ values between 7.1 and 23.3. Surprisingly, the ORR activity shows a volcano relationship with the p*K*_a_ of the ionic liquids for both Au and Pt. Potential-dependent in situ ATR-SEIRAS tracked the stretching frequency of hydrogen-bonded protic cations from the ionic liquids influenced by the ORR reaction intermediates. The observed red shift of the N–H^+^ peak could be correlated with hydrogen bond interaction with ORR intermediates. They found the strongest hydrogen bond interaction for the Au catalyst to be N–H^+^···OOH_ad_ for the [DEMA][NTf_2_] ionic liquid. Interestingly, the p*K*_a_ value of this protic cation (10.3) and that of the ORR product H_2_O_2_ on Au (11.6) are very similar. As the conversion of OOH_ad_ to H_2_O_2_ is the rate-limiting step for ORR on Au, the authors associated the maximum ORR activity on Au with the strongest N–H^+^···OOH_ad_ hydrogen bond interaction. Similarly, the maximum ORR activity on Pt in the presence of [MTBD][NTf_2_] was associated with the strongest N–H^+^···OH_ad_ hydrogen bond interaction, which is in good agreement with OH_ad_ being the main adsorbed ORR intermediate on Pt, as observed via in situ surface-enhanced Raman spectroscopy. Simulations of proton-coupled electron transfer kinetics revealed that the enhanced hydrogen bonding in the presence of ionic liquids increases the proton tunneling kinetics while the activation-free energies remain unchanged. This study provides fundamentally new insights and recommends catalyst design strategies beyond conventional techniques by examining noncovalent hydrogen bonding configurations and solvation environments in the EDL.

The hydrogen reactions (HER/HOR) are essential in the context of transitioning to a carbon-free, hydrogen-based future. Therefore, a lot of research is devoted to enhancing hydrogen catalysts and unraveling the nature of the hydrogen reaction mechanisms to boost HER and HOR activities. This is only achievable by using state-of-the-art techniques to identify and understand the key processes at the solid–liquid interface in the EDL. One extensively discussed issue of hydrogen electrocatalysis constitutes the pH effect, where the activity differs by orders of magnitude in alkaline and acidic reaction environments. Despite intensive research and major advancements, the situation around the origin of the pH effect is still not resolved. While several studies support the beneficial influence of specifically coadsorbed AM^+^ cations on the adsorption of H and OH inducing the pH effect via direct AM^+^ adsorption or via forming H_2_O–AM^+^–H_ad_/OH_ad_ clusters,^475,516−519^ many investigations associate H and OH adsorption and, therefore, pH-dependent hydrogen activity rather with the structure of the interfacial water in the EDL.^115,520−524^ In a recent study, Zhu et al., for the first time, successfully monitored changes in the hydrogen and water binding energies in the EDL on Pt surfaces at different pHs using ATR-SEIRAS, which led to the observation of weakened hydrogen binding energies as the pH increased.^525^ By taking structural changes in the interfacial water network, cation/anion effects, and changes in the Pt–H vibrational band into account, they concluded that the pH effect should strongly depend on the EDL structure as a synergistic consequence of the modified electric field, the coverage of H_ad_, H_ad_–H_2_O, and Pt–H_2_O interactions. However, Wang et al. further observed that introducing solvated cations (Na^+^ ions) into the water caused a dynamic change in the structure of interfacial water on Pd single crystals.^526^ A combination of in situ Raman spectroscopy and computational methods delivered evidence of potential- and Na^+^-induced rearrangement of interfacial water from a random disordered to an ordered structure, which was concluded to enhance the HER reaction rates. Therefore, the authors suggested that cations indeed affect HER kinetics when utilizing appropriate cation tuning of the EDL.

**Figure 39:**
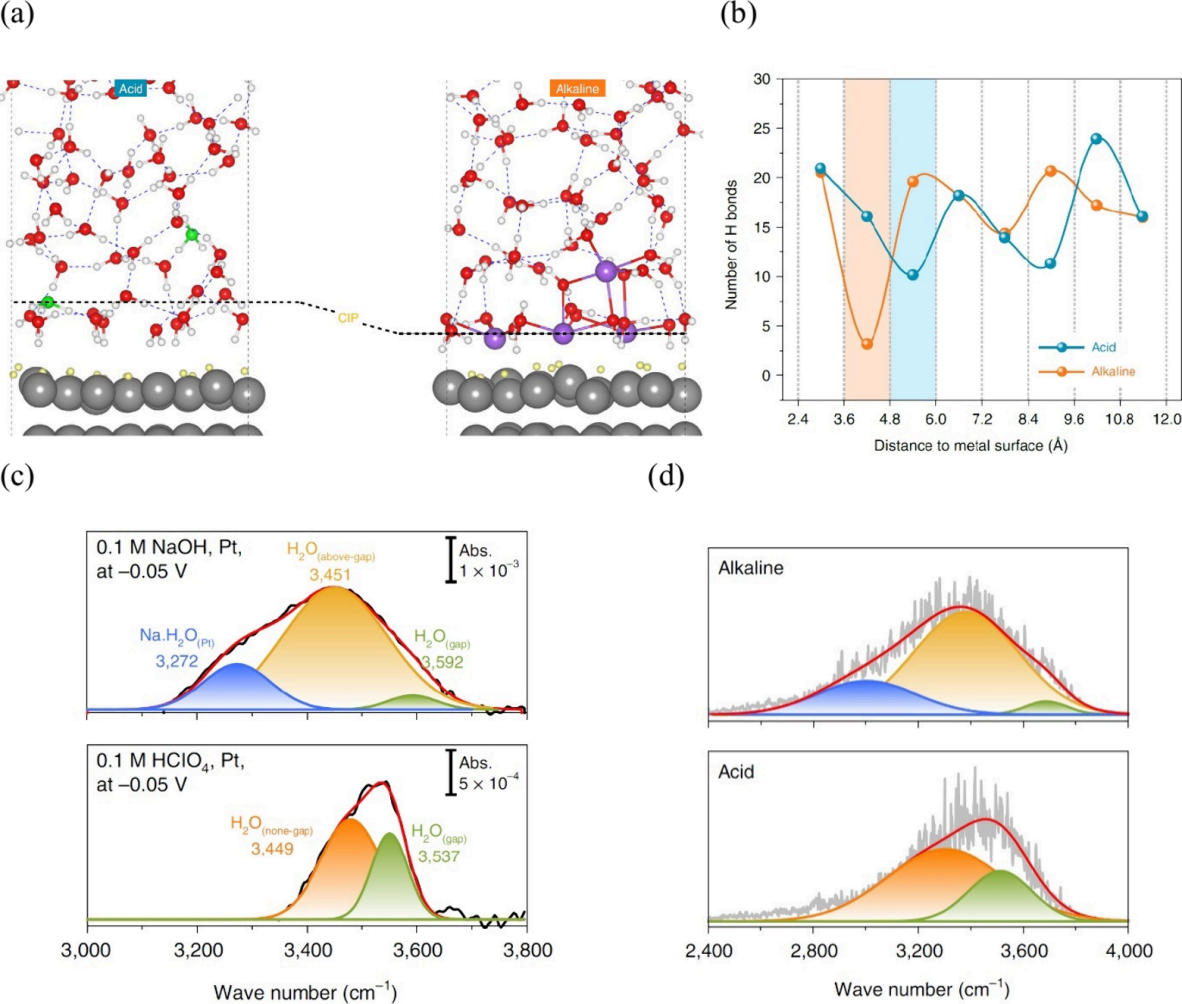
(a) AIMD results illustrating the EDL structure at the Pt(111)/liquid interface in acidic and alkaline electrolyte conditions. The term “CIP” denotes the closest ion planes, showing significant differences for the acidic (∼4.26 Å) and alkaline (∼2.92 Å) electrolyte conditions. (b) Quantitative analysis of the number of hydrogen bonds as a function of normal surface distance, highlighting gap zones of interfacial water. (c) O–H stretching vibration peaks determined from experimental SEIRAS at the Pt(111)/liquid interface, obtained in 0.1 M NaOH and HClO_4_ at −0.05 V. Gaussian fits emphasize the deconvolution of the O–H stretching vibration peaks into two or three components for acidic or alkaline electrolyte, respectively. (d) Calculated vibrational density of states (VDOS) for the O–H stretching mode, depicting the separation into two or three distinct components for acidic or alkaline environments, respectively. Reprinted with permission from ref (527). Copyright 2022 Springer Nature.

In a recent study, Li et al. attributed the large pH effect of the hydrogen kinetics to the different connectivity of hydrogen bond networks in the EDL.^527^ Using Pt(111) as a model catalyst system, they compared the EDLs of acid and alkaline interfaces from in situ ATR-SEIRAS experiments with those of AIMD simulations and the computed vibrational density of states of water molecules. The combination of theoretical calculations and experimental findings confirmed a reduced hydrogen bond connectivity in alkaline media due to a depletion of solvated water molecules near the interface as a result of the strong interaction between the cations (Na^+^ in their study) and their solvation water molecules (Figure 39a). This, in turn, is suggested to affect the hydrogen transfer processes essential for HER/HOR. While it has been argued that the difference in kinetics stems from the different hydrogen sources (H_3_O^+^ in acid, H_2_O in base), Li et al. could not entirely attribute the 2 orders of magnitude difference in activity to the change of the hydrogen source. Based on their results, they rather emphasized the hydrogen transfer from the electrolyte bulk to interfacial water molecules through the hydrogen bond network, which was also suggested by a recent theoretical work of Huang et al.^528^ The diminished number of hydrogen bonds in alkaline media near the interface (Figure 39b) is supposed to strongly decrease the available hydrogen transfer channels and leads to slower HER/HOR kinetics, as suggested by comparing the O–H stretching bonds of ATR-SEIRAS (Figure 39c) and DFT simulation results (Figure 39d). While the depletion of hydrogen bonding increases with decreasing electrode potential in alkaline environment, there is only negligible change with potential in an acidic environment.

To prove their hypothesis, Li et al.^527^ investigated Pt_3_Ru(111) as alloying with Ru was reported to enhance the HER/HOR activities due to the oxophilic character of Ru to adsorb more OH/H_2_O. When comparing Pt(111) with Pt_3_Ru(111) with and without OH_ad_, an obvious increase in water concentration and hydrogen bond connectivity was observed in the previously depleted region close to the interface of the Pt_3_Ru(111) electrode with adsorbed OH, while no change was observed for Pt_3_Ru(111) without OH_ad_. This supports the findings of the authors that introducing OH into the interface leads to a rearrangement of the solvation environment of the Na^+^ ions, which, in turn, gives more space to water molecules in the EDL to form hydrogen bond networks. By this, the authors argued that the EDL, more precisely the hydrogen bond network in the EDL, is the key parameter to elucidate the HER/HOR kinetics in alkaline and acidic media rather than adsorption energetics or water formation/dissociation barriers.

Furthermore, Chen et al. recently reported on the efficient HER catalysis of Ru catalysts by changing the valency in alkaline media via in situ Raman spectroscopy and DFT calculations.^529^ They tracked interfacial water, adsorbed hydrogen, and adsorbed OH and associated adsorption and interaction phenomena with the valence state of Ru surfaces, further emphasizing the importance of the EDL structure on HER activity. Further, they observed that OH_ad_ promotes interfacial water dissociation by acting as an electronically favorable acceptor and geometrically favorable proton donor. In contrast, they suggested a local cation-tuning effect of Na^+^ in the surrounding interfacial water structure together with increasing work functions for high-valence Ru sites to boost the interaction and facilitate water dissociation and, therefore, HER kinetics in alkaline media. Such an influence of AM^+^ cations on the interfacial water layer in the EDL, specifically on the adsorption properties of adsorbed OH, in the context of alkaline HER was also reported by Shah et al. by combining EIS data, a unique surface–adsorbate ETS approach, and DFT calculations.^55^ They suggest that cations are not directly bonded to the Pt catalyst surface or OH_ad_, but they are separated by a single layer of water molecules. Importantly, smaller cations (Li^+^) are expected to increase the surface coverage of OH_ad_ at the catalyst surface, where they function as electronically favorable acceptors or geometrically favorable proton donors. However, the influence of adsorbed OH at the solid–liquid interface of the EDL on the pH effect and reaction kinetics of HER/HOR has been critically debated in past studies.^530−535^ While certain studies suggest the OH_ad_ to be rate-controlling for HER and HOR due to its influence on water dissociation, other investigations either emphasize the structure of interfacial water and the EDL as the parameter to control hydrogen kinetics or neglect the importance of OH_ad_ and completely rule out the decisive role of OH_ad_ for HER/HOR activity. The difficulty lies in the complexity of the investigated problem itself. Various groups utilize different techniques with different methods to analyze various materials in a variety of environments. Consequently, it is not surprising that there is seemingly no clear consensus yet.

In a recent study, Su et al. investigated the HOR/HER kinetics of various precious metal catalysts (Pt, Rh, Rh_2_P, Ru, and Ru_2_P) in electrolytes with a wide range of pHs based on the discussion above.^263^ Interestingly, the authors did not observe a linear relationship between the kinetic HER current and pH, but they measured an inflection-point behavior for all investigated catalyst materials, suggesting that hydrogen binding energy is not the sole pH-universal descriptor of the HER/HOR activity. In fact, they revealed that this inflection point linearly scales with the OH binding energies of various electrocatalysts. Pt and Ru_2_P exhibited the highest and lowest inflection point pHs, the weakest and strongest OH binding energies, and the largest and smallest kinetic gaps between acidic and alkaline HER/HOR, respectively. Therefore, the authors proposed the OH binding energy as a suitable descriptor for the HER/HOR kinetics, supported by a triple-path microkinetic model. According to their modeling, the linear connection between the OH binding energy dependent inflection point pH and the HER/HOR kinetic gap implied that OH adsorption is an essential parameter for HER/HOR catalysis. They proposed an improved hydrogen bond network in the alkaline EDL for electrocatalysts with enhanced OH binding energy, as also suggested by Li et al.^527^ According to the authors, optimized OH binding energy, therefore, leads to stronger interaction of adsorbed OH with electrolyte cations, which, in turn, relieves surrounding water molecules and improves the hydrogen bond network in the EDL resulting in enhanced HER/HOR kinetics.

In summary, adsorption phenomena play an essential role in electrocatalysis. Reaction intermediates, spectator ions, coadsorption, and other factors can decisively alter reaction kinetics, which was shown exemplarily through some of the most relevant publications over the last years in the field of electrocatalysis for several relevant electrochemical systems (single-crystal and polycrystalline surfaces, nanoparticles, metal oxides) and reactions (CO_2_R, ORR, OER, HER/HOR). We highlighted the development from early studies on adsorption and reaction intermediates to the most recent examples of interfacial electrochemistry investigations, combining advanced experimental and computational evaluations. While a general correlation between universal double-layer properties and specific reaction processes seems unfeasible at this point, various works have been highlighted throughout this section that demonstrate new hypotheses, mechanisms, and efforts to establish a better understanding and find such an interrelation. In this context, we put emphasis on the importance of the electrical double layer structure and phenomena to get more profound insights into current electrocatalytic systems and to give the reader an orientation toward innovative design of next-generation electrochemical materials.

## 9 The Double Layer at Solid/Solid Interfaces

Solid oxide fuel cells (SOFCs) are excellent examples of applications involving solid–solid interfaces in the field of electrocatalysis. Since oxygen-ion-conducting solid electrolytes tend to exhibit poor conductivity at lower temperatures, these cells typically require operation temperatures above 400 °C. A typical SOFC is composed of a porous oxide-based cathode, a dense ceramic electrolyte, and a porous ceramic–metal composite anode.^536,537^ The formation of the EDL at the interface between the ion-conducting and electron-conducting phases in the nanocomposite electrode is believed to significantly influence oxygen transport mechanisms within the electrode.^538,539^ For this reason, understanding how the EDL affects ion transport and reactivity at these interfaces can lead to improved design, enabling lower operational temperatures and increased efficiency.

**Figure 40:**
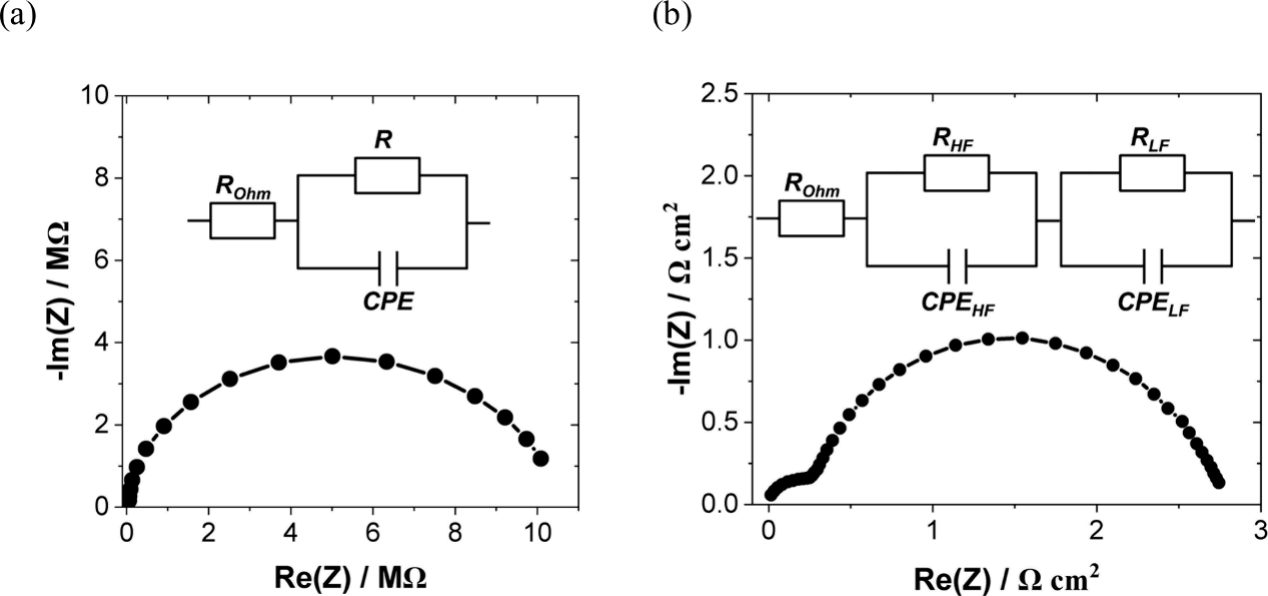
(a) Nyquist plot of the EIS data obtained from a dense Ni/YSZ electrode at 800 °C under open circuit with one depressed semicircle.^548^ (b) Nyquist plot of the EIS data obtained from a porous Ni/YSZ electrode at 700 °C under open circuit with two depressed semicircles.^543^ The equivalent circuits commonly used to fit the impedance response of SOFC anodes are displayed in the corresponding top right corners. (a) Adapted from ref (548). CC BY 3.0. (b) Adapted with permission from ref (543). Copyright 2013 Elsevier.

The research primarily focuses on the double layers between metal anodes and yttria-stabilized zirconia (YSZ) electrolytes. While some early studies investigated the capacitance of electrode–electrolyte interfaces analyzing potential step chronoamperometry,^540^ electrochemical impedance spectroscopy proved to provide more reliable results. Depending on the supplied atmosphere and the applied potential one^541,542^ or two^543,544^ depressed semicircles were observed in Nyquist plots, as displayed in Figure 40. The impedance response of the anode is commonly fitted using an equivalent circuit model (ECM) consisting of one or two parallel connections of resistance and a constant phase element in series with an ohmic resistance and sometimes an additional inductive contribution. The high-frequency arc is attributed to the electrode–electrolyte interface, combining the charging of the double layer and the Faradaic reaction. The low-frequency arc is associated with the gas–electrode boundary, combining gas diffusion and adsorption.^545,546^ The double-layer capacitance can be calculated from the CPE impedance  using the correlation .^547^ Here, ω is the angular frequency, *n* is the CPE exponent, *B* is the CPE prefactor, *C*_dl_ is the double-layer capacity, and *R*_Ω_ is the ohmic resistance.

An overview of some measured double-layer capacitances at open-circuit voltage from the literature is given in Table 7. The reported values for the most common anode materials, Ni and polycrystalline YSZ, range between 1 and 232 μF cm^–2^. However, the capacitance is usually only normalized to the geometric electrode surface area. Therefore, measurements on different electrode compositions and preparation methods are hardly comparable. Yashiro et al.^549^ and Takeda et al.^550^ investigated the relationship between the electrode capacitance and the double-phase-boundary area between Ni and YSZ. Fitting an equivalent circuit to impedance spectra recorded for model electrodes with different areas, they calculated an area-specific capacitance of 130–210 μF cm^–2^. The relation between double-phase boundary and capacitance was further validated in porous Ni/YSZ electrodes. Comparing the area-specific capacitance with theoretical values for different phenomena, they concluded that the capacitance mainly stems from oxide ions at the Ni/YSZ interface.

**Table 7:** Overview of Double Layer Capacitance Values from the Literature for the Interface between Common SOFC Anode Materials and YSZ

electrode	electrolyte	method	atmosphere	*T* [°C]	*C* [μF cm^–2^]	ref
Pt (porous)	YSZ	EIS	O_2_	856–1017	10–300	(541)
Au (porous)	YSZ	EIS	O_2_	1041	30–300	(541)
Pd (porous)	YSZ	EIS	O_2_	900–1050	∼0.1	(541)
Ni (dense)	YSZ	EIS	H_2_/H_2_O	650–750	20–150	(545)
Pt (dense)	YSZ	EIS	H_2_/H_2_O	600–800	90–170	(545)
Au (dense)	YSZ	EIS	H_2_/H_2_O	700–800	140–200	(545)
Ni (dense)	YSZ, polycrystalline	EIS	H_2_/H_2_O	673–823	225–232	(542)
Ni (dense)	YSZ, ⟨100⟩	EIS	H_2_/H_2_O	723–823	162–186	(542)
Ni (dense)	YSZ, ⟨111⟩	EIS	H_2_/H_2_O	723–823	252–263	(542)
Ni (dense)	YSZ, ⟨110⟩	EIS	H_2_/H_2_O	723–823	264–307	(542)
Ni–YSZ (porous)	YSZ	EIS	H_2_/H_2_O	700–800	22–46	(543)
Ni–YSZ (porous)	YSZ	EIS	H_2_/H_2_O	800	1–30	(552)
Ni–YSZ (porous)	YSZ	EIS	H_2_/H_2_O	650–800	4.8–20.3	(546)

**Figure 41:**
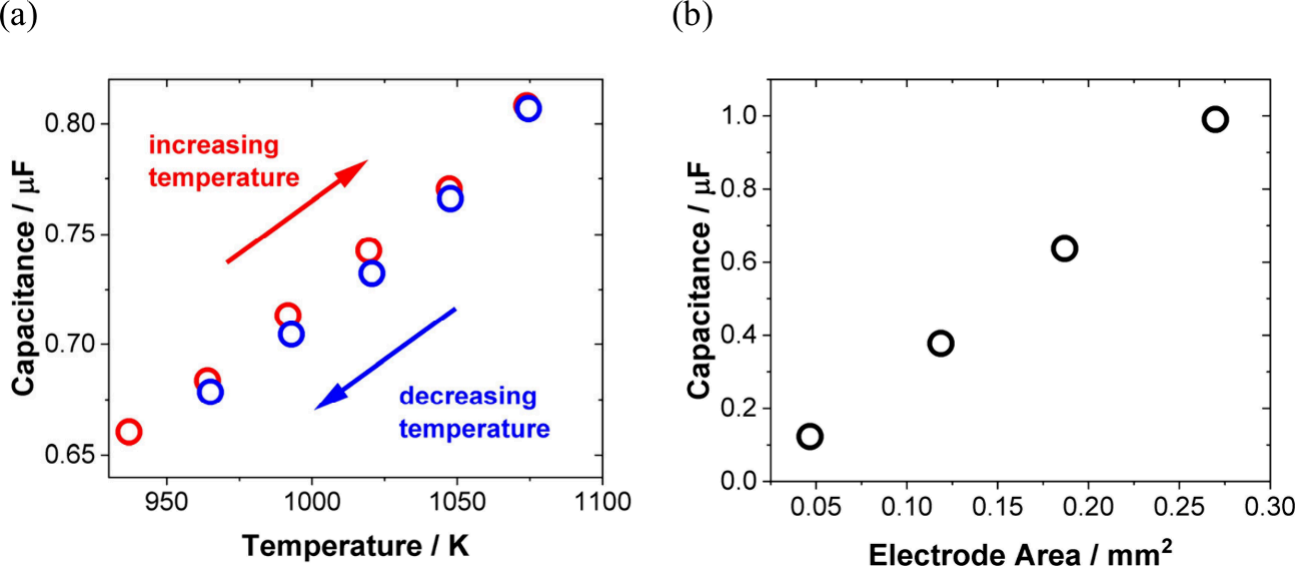
(a) Temperature dependence of the electrode capacitance. The measurement was performed on an electrode with an area of 0.280 mm^2^ by first decreasing and then increasing the temperature. Conditions: 2.5% H_2_/0.15% H_2_O/balance Ar, Ni needle. (b) Electrode capacitance values of circular electrodes with varying diameters plotted as a function of the electrode area. Adapted from ref (551). CC BY 4.0.

Doppler et al.^551^ conducted similar experiments and found an area-specific capacitance of 301 ± 3 μF cm^–2^ (see Figure 41). They concluded that the capacitance is too high to be explained by a Helmholtz-type double layer. Instead, they associated two separate phenomena with the high capacitance values that are dominant depending on the oxygen partial pressure. In the regime above 7 × 10^–24^ bar, a mechanism involving the oxidation of Ni and the formation of an interfacial Ni_1–*x*_O layer was proposed. For smaller oxygen partial pressures, protons stored at the interlayer between Ni and YSZ may explain the electrode capacitance.

To conclude, we briefly summarize the similarities and dissimilarities between solid/solid interfaces and solid/liquid interfaces and give corresponding suggestions on how models should be improved.

The double layer at solid/liquid interfaces is commonly described by the Gouy–Chapman–Stern model, separating the charge profile in the electrolyte into a small inner layer and a large diffuse layer. Similar regions are also observed in solid electrolytes. However, the mathematical description of the layers differs significantly. We discussed two main differences: First, the ion concentration in a solid electrolyte is much higher than in dilute liquid electrolytes, and therefore, the Debye–Hückel theory does not hold anymore. Second, the defect-rich crystal lattice in solid electrolytes is not only the origin of the ion conduction but also limits the ion concentration in the space charge layer.

As the Debye–Hückel model is not applicable to describe the ion interactions in solid electrolytes, the width of the SCLs may be determined by the quasi-Fermi electrochemical potential, suggesting that the SCL is mainly influenced by bulk properties.^553,554^ However, the continuum models used to predict SCL properties are limited to length scales above a few interatomic distances.^148,555^ Further model improvements should focus on the first few atomic layers of the solid/solid interface, as a single layer of large vacancy concentrations may form a compact electrical double layer.^153,556,557^ Further, efforts should be made to extend simulations to polycrystalline materials.^558^

## 10 Summary and Outlook

In this review, we attempted to present an overview of recent progress in both experimental and theoretical understanding of basic EDL properties with a primary emphasis on metal/liquid interfaces. Such interfaces play a central role in many electrochemical energy storage and conversion applications, most prominently in electrocatalysis. This explains a tremendous practical interest in studying such interfaces during the past years. Unfortunately, relatively few investigations have so far focused on the fundamental science of the EDL, limiting our progress in the practical application of electrochemical systems. In this contribution, we have particularly discussed the roles of electrolyte chemistry, electrode composition, and structure in the basic properties of the EDL, such as the *C*_dl_, pzc, pzfc, and PME. We have outlined a series of experimental and theoretical approaches used to assess and predict these key EDL quantities and have further discussed how they are manifested in electrocatalysis. Based on our discussion, we want to stress several crucial points.

One important theme in designing efficient electrochemical energy systems in general and in electrocatalysis, in particular, has been the quest for activity descriptors. Such descriptors should be simple enough to avoid complicated and time-consuming calculations or experiments, serving as a useful guide when predicting material properties. In the context of electrocatalysis, several descriptors were used. One of the most widely used is the binding energy of reaction intermediates, which is related to the Sabatier principle suggesting that their adsorption should be neither too strong nor too weak for ideal reactivity. This enabled very efficient computational studies aimed at evaluating adsorption energies to be displayed in the form of the so-called volcano plots with the peak of the volcano corresponding to the most active catalyst. Such investigations were especially useful in predicting and rationalizing activity trends.

However, we have learned that other key factors could be considered as reactivity determinants. For example, the energetics of water reorganization to accommodate interfacial charge transfer was identified as an important parameter that can control HER activity under certain reaction conditions.^113−115^ This descriptor is closely related to the PME concept. This is, however, in contrast to other investigations suggesting that greatly weakened water adsorption at high pH is the major cause of pH-dependent hydrogen binding on a noble metal like Pt.

To explain two orders of magnitude differences in HER activity between acidic and alkaline conditions, other descriptors were invoked such as the H-bonding network connectivity.^527^ Specifically, it was demonstrated that the EDLs of alkaline solutions are characterized by significantly diminished connectivity of H-bonding networks as compared with acidic solutions. In addition, it was found that the adsorption of OH species can indirectly affect the catalyst’s reactivity by increasing the H-bonding network connectivity in the EDL. In a subsequent study,^559^ it was thus proposed that the HER/HOR kinetics of Pt in acid and base is governed by diffusion of protons and hydroxyls, respectively, through the H-bonding network of interfacial water via the Grotthuss mechanism.

Overall, these EDL-based descriptors highlight the critical role played by the EDL in determining electrocatalytic activity beyond the traditionally analyzed binding energy of reaction intermediates. It should be noted, however, that we are still far from a comprehensive understanding of how EDL properties define electrocatalytic activity as a function of reaction conditions and how the discussed descriptors could be interrelated.

Another important aspect necessary to propel forward the basic science of electrochemical metal/water interfaces is the need for more methodological developments. We believe that such methodological activities are often underappreciated by the research community. From a theoretical perspective, more methodological advancements are necessary, for example, to efficiently perform all-atom simulations under constant-potential conditions.

Next, the high complexity of the EDL requires more synergistic investigations combining experiment and theory. We note that such joint theory–experiment studies may also benefit from the incorporation of advanced machine learning (ML) methods. For example, ML approaches such as neural-network-based potentials can help overcome spatiotemporal limitations of ab initio molecular dynamics methods. We also want to underline the need to perform highly accurate experiments on model systems under well-controlled conditions (e.g., working with single crystals, high-purity solutions). Such studies should also aim to correlate key parameters describing the EDL, whether microscopically or macroscopically, with the electrocatalytic activity under comparable conditions.

Finally, we would like to stress the importance of a critical assessment of experimental and computational errors and limitations since electrochemical quantities such as the double-layer capacitance are often highly sensitive to experimental conditions and details of computational methodology.

## Abbreviations

EDL: electric double layer
*C*_dl_: double-layer capacitance
pzc: potential of zero charge
PME: potential of maximum entropy
STM: scanning tunneling microscopy
XAS: X-ray absorption spectroscopy
EC-IR: electrochemical infrared spectroscopy
ETS: electrical transport spectroscopy
SFG: sum frequency generation
CV: cyclic voltammetry
LSV: linear sweep voltammetry
GCD: galvanostatic charge/discharge
EIS: electrochemical impedance spectroscopy
LICT: laser-induced current transient
PB: Poisson–Boltzmann
cDFT: classical density functional theory
DFT: density functional theory
OER: oxygen evolution reaction
CHE: computational hydrogen electrode
AM: alkali metal
IHP: inner Helmholtz plane
OHP: outer Helmholtz plane
pzfc: potential of zero free charge
pztc: potential of zero total charge
IL: ionic liquid
SSE: solid state electrolyte
SCL: space charge layer
XRR: X-ray reflectometry
SXS: surface X-ray scattering
XSW: X-ray standing wave
SXRD: surface X-ray diffraction
TSXRD: transmission surface X-ray diffraction
SERS: surface-enhanced Raman spectroscopy
XAS: X-ray absorption spectroscopy
EXAFS: extended X-ray absorption fine structure
XANES: X-ray absorption near edge structure
NEXAFS: near edge X-ray absorption fine structure
XES: X-ray emission spectroscopy
XPS: X-ray photoelectron spectroscopy
EC-IR: electrochemical infrared spectroscopy
FTIR: Fourier transform infrared spectroscopy
ATR: attenuated total reflection
ETS: electrical transport spectroscopy
VSFG: vibrational sum frequency generation
EC-STM: electrochemical scanning tunneling microscopy
EC-cell: electrochemical cell
EEC: electrical equivalent circuit
CPE: constant phase element
dEIS: dynamic electrochemical impedance spectroscopy
SEPM: scanning electrochemical probe microscopy
SECM: scanning electrochemical microscopy
SECCM: scanning electrochemical cell microscopy
SICM: scanning ion conductance microscopy
LEIS: localized electrochemical impedance spectroscopy
LITJ: laser-induced temperature jump
AIMD: ab initio molecular dynamics
ML: machine learning
NNP: neural network potentials
RMSE: root-mean-square error
TFY: total fluorescence yield
TEY: total electron yield
ND: nondonor
DD: double donor
SD: single donor
SD^∥^: single donor (parallel orientation)
SD^⊥^: single donor (perpendicular orientation)
VDOS: velocity density of states
*N*_donor_: hydrogen bond donors
HR: high resolution
QRCE: quasi-reference counter electrode
ML: monolayer
HEA: high entropy alloys
WF: work function
EDX: energy dispersive X-ray
HER: hydrogen evolution reaction
ORR: oxygen reduction reaction
CO_2_RR: carbon dioxide reduction reaction
HOR: hydrogen oxidation reaction
GCN: generalized coordination numbers
EBSD: electron backscatter diffraction
IPF: inverse pole figure
HPRR: hydrogen peroxide reduction reaction
SOFC: solid oxide fuel cells
ECM: equivalent circuit model
pc: polycrystalline
